# Supplementary material for: Sibship size, birth order and risk of asthma and allergy: protocol for a systematic review and meta-analysis
Source: BMJ Open. 2021 Aug 23;11(8):e045795. doi: 10.1136/bmjopen-2020-045795 (PMC8383851; doi:10.1136/bmjopen-2020-045795)
Supplement: Supplementary data [file bmjopen-2020-045795supp002.pdf]

Appendix to

Sibship Size, Birth Order and Risk of Asthma and Allergy: Protocol for a Systematic Review and Meta-Analysis

Daniil Lisik, Athina Ioannidou, Gregorio Paolo Milani, Sungkutu Nyassi, Saliha Selin Özuygur Ermis, Giulia Spolidoro, Emma Goksör, Göran Wennergren, Bright I Nwaru

Appendix 2: Search Strategies

Colorization

Red: controlled vocabulary/thesaurus

Blue: free-text

Green: referral to search query component (table row; #)

AMED

#	**Search term(s)**
1	(birth order* or birth rank* or multiple birth* or parity).mp.
2	exp Family Characteristics/ or (family characteristic* or family size* or family structure* or family demograph* or family composition or household size* or household demograph* or household composition).mp.
3	exp Sibling Relations/ or (sibling* or sister* or brother* or sibship size* or sibship*).mp.
4	or/1-3
5	exp Asthma/ or (bronchial asthma* or exercise-induced asthma* or exercise-induced bronchospasm* or asthma* or respiratory hypersensitivit* or airway hyper responsiveness or airway hyper-responsiveness or respiratory hyper responsiveness or respiratory hyper-responsiveness or wheez*).mp.
6	exp Hypersensitivity/ or exp Hypersensitivity Immediate/ or exp Hypersensitivity Delayed/ or (immediate hypersensitivit* or delayed hypersensitivit* or IgE-mediated hypersensitivit* or type I hypersensitivit* or type IV hypersensitivit* or atopic sensitization or atop* or allergic sensitization or allerg*).mp.
7	exp Dermatitis/ or exp Anaphylaxis/ or (atopic dermatitis or dermatitis or neurodermatiti* or besniers prurigo or besnier prurigo or atopic eczema or eczema or urticari* or anaphyla* or quinckes edema or quincke edema or angioneurotic edema or angioedema or hives).mp.
8	exp Food Hypersensitivity/ or (food hypersensitivit* or food allerg* or egg hypersensitivit* or egg allerg* or milk hypersensitivit* or milk allerg* or shellfish hypersensitivit* or shellfish allerg* or wheat hypersensitivit* or wheat allerg* or nut hypersensitivit* or nut allerg* or peanut hypersensitivit* or peanut allerg* or groundnut hypersensitivit* or groundnut allerg*).mp.

Lisik D, *et al. BMJ Open* 2022; 11:e045795. doi: 10.1136/bmjopen-2020-045795

9	exp Rhinitis/ or exp Conjunctivitis/ or (allergic rhinoconjunctiviti* or rhinoconjunctiviti* or allergic rhiniti* or seasonal allergic rhiniti* or perennial allergic rhiniti* or rhiniti* or allergic conjunctiviti* or vernal keratoconjunctiviti* or vernal conjunctiviti* or giant papillary conjunctiviti* or hay fever or hayfever or pollinosis or nasal catarrh*).mp.
10	or/5-9
11	4 and 10
Full query<br>((birth order* or birth rank* or multiple birth* or parity).mp. or exp Family Characteristics/ or (family characteristic* or family size* or family structure* or family demograph* or family composition or household size* or household demograph* or household composition).mp. or exp Sibling Relations/ or (sibling* or sister* or brother* or sibship size* or sibship*).mp.) and (exp Asthma/ or (bronchial asthma* or exercise-induced asthma* or exercise-induced bronchospasm* or asthma* or respiratory hypersensitivit* or airway hyper responsiveness or airway hyper-responsiveness or respiratory hyper responsiveness or respiratory hyper-responsiveness or wheez*).mp. or exp Hypersensitivity/ or exp Hypersensitivity Immediate/ or exp Hypersensitivity Delayed/ or (immediate hypersensitivit* or delayed hypersensitivit* or IgE-mediated hypersensitivit* or type I hypersensitivit* or type IV hypersensitivit* or atopic sensitization or atop* or allergic sensitization or allerg*).mp. or exp Dermatitis/ or exp Anaphylaxis/ or (atopic dermatitis or dermatitis or neurodermatiti* or besniers prurigo or besnier prurigo or atopic eczema or eczema or urticari* or anaphyla* or quinckes edema or quincke edema or angioneurotic edema or angioedema or hives).mp. or exp Food Hypersensitivity/ or (food hypersensitivit* or food allerg* or egg hypersensitivit* or egg allerg* or milk hypersensitivit* or milk allerg* or shellfish hypersensitivit* or shellfish allerg* or wheat hypersensitivit* or wheat allerg* or nut hypersensitivit* or nut allerg* or peanut hypersensitivit* or peanut allerg* or groundnut hypersensitivit* or groundnut allerg*).mp. or exp Rhinitis/ or exp Conjunctivitis/ or (allergic rhinoconjunctiviti* or rhinoconjunctiviti* or allergic rhiniti* or seasonal allergic rhiniti* or perennial allergic rhiniti* or rhiniti* or allergic conjunctiviti* or vernal keratoconjunctiviti* or vernal conjunctiviti* or giant papillary conjunctiviti* or hay fever or hayfever or pollinosis or nasal catarrh*).mp.)	

exp = include all narrower subject headings; mp= abstract, heading words, title

CABI; OAIster; Open Access Theses and Dissertations; Open Grey; ProQuest Dissertations & Theses Global; SciELO; WHO Global Index Medicus

#	Search term(s)
1	"birth order" OR "multiple births" OR "birth rank" OR "parity"
2	"family characteristics" OR "family size" OR "family structure" OR "family demography" OR "family composition" OR "household size" OR "household demography" OR "household composition"
3	"siblings" OR "sibling relations" OR "sister" OR "brother" OR "sibship"
4	"exercise-induced bronchospasm" OR "asthma" OR "airway hyper-responsiveness" OR "respiratory hyper-responsiveness" OR "wheeze" OR "wheezing"
5	”hypersensitivity" OR "atopic sensitization" OR "atopy" OR "allergic sensitization" OR "allergic disease" OR "allergic condition" OR "allergy" OR "allergies"

Lisik D, *et al. BMJ Open* 2022; 11:e045795. doi: 10.1136/bmjopen-2020-045795

6	"dermatitis" OR "eczema" OR "neurodermatitis" OR "besnier's prurigo" OR "urticaria" OR "anaphylaxis" OR "anaphylactic shock" OR "quincke's edema" OR "angionuerotic edema" OR "angioedema" OR "hives"
8	"rhinoconjunctivitis" OR "rhinitis" OR "allergic conjunctivitis" OR "vernal keratoconjunctivitis" OR "vernal conjunctivitis" OR "giant papillary conjunctivitis" OR "hay fever" OR "pollinosis" OR "pollenosis" OR "nasal catarrh"
9	1 OR 2 OR 3
10	4 OR 5 OR 6 OR 7 OR 8
11	9 AND 10
Full query	("birth order" OR "multiple births" OR "birth rank" OR "parity" OR "family characteristics" OR "family size" OR "family structure" OR "family demography" OR "family composition" OR "household size" OR "household demography" OR "household composition" OR "siblings" OR "sibling relations" OR "sister" OR "brother" OR "sibship") AND ("exercise-induced bronchospasm" OR "asthma" OR "airway hyper-responsiveness" OR "respiratory hyper-responsiveness" OR "wheeze" OR "wheezing" OR "hypersensitivity" OR "atopic sensitization" OR "atopy" OR "allergic sensitization" OR "allergic disease" OR "allergic condition" OR "allergy" OR "allergies" OR "dermatitis" OR "eczema" OR "neurodermatitis" OR "besnier's prurigo" OR "urticaria" OR "anaphylaxis" OR "anaphylactic shock" OR "quincke's edema" OR "angionuerotic edema" OR "angioedema" OR "hives" OR "rhinoconjunctivitis" OR "rhinitis" OR "allergic conjunctivitis" OR "vernal keratoconjunctivitis" OR "vernal conjunctivitis" OR "giant papillary conjunctivitis" OR "hay fever" OR "pollinosis" OR "pollenosis" OR "nasal catarrh")

CINAHL

#	Search term(s)
1	(MH 'Birth Order+') OR (MH 'Parity+') OR 'birth order*' OR 'birth rank*' OR 'multiple birth*' OR 'parity'
2	(MH 'Family Characteristics+') OR (MH 'Family Health+') OR 'family charactersitic*' OR 'family size*' OR 'family structure*' OR 'family demograph*' OR 'family composition' OR 'household size*' OR 'household demograph*' OR 'household composition'
3	(MH 'Siblings+') OR (MH 'Sibling Relations+') OR 'sibling*' OR 'sister*' OR 'brother*' OR 'sibship size*' OR 'sibship*'
4	(MH 'Asthma+') OR (MH 'Respiratory Hypersensitivity+') OR 'bronchial asthma*' OR 'exercise-induced asthma' OR 'asthma*' OR 'exercise-induced bronchospasm' OR 'respiratory hypersensitivit*' OR 'airway hyper responsiveness' OR 'airway hyper-responsiveness' OR 'respiratory hyper responsiveness' OR 'respiratory hyper-responsiveness' OR 'wheez*'
5	(MH 'Hypersensitivity+') OR (MH 'Hypersensitivity, Immediate+') OR (MH 'Hypersensitivity, Delayed+') OR (MH 'Allergy and Immunology+') OR 'immedate hypersensitivit*' OR 'delayed hypersensitivit*' OR 'IgE-mediated hypersensitivit*' OR 'type I hypersensitivit*' OR 'type IV hypersensitivit*' OR 'hypersensitivit*' OR 'atopic sensitiziation' OR 'atop*' OR 'allergic sensitization' OR 'allergic disease* OR 'allerg*'

Lisik D, et al. BMJ Open 2022; 11:e045795. doi: 10.1136/bmjopen-2020-045795

6	(MH ’Dermatitis, Atopic+’) OR (MH ’Eczema+’) OR (MH ‘Angioedema+’) OR (MH ‘Anaphylaxis+’) OR (MH ’Urticaria+’) OR ’atopic dermatitis’ OR ’dermatitis’ OR ’atopic eczema’ OR ’eczema’ OR ’nerudoarmatiti*’ OR “besnier’s prurigo” OR ‘besniers prurigo’ OR ‘besnier prurigo’ OR ’urticari*’ OR ’hives’ OR ’anaphyla*’ OR ”quincke’s edema” OR ’quinckes edema’ OR ’quincke edema’ OR ’angioneurotic edema’ OR ’angioedema’
7	(MH ’Food Hypersensitivity+’) OR ’food hypersensitivit*’ OR ’food allerg*’ OR ’egg hypersensitivit*’ OR ’egg allerg*’ OR ’milk hypersensitivit*’ OR ’milk allerg*’ OR ’shellfish hypersensitivit*’ OR ’shellfish allerg*’ OR ’wheat hypersensitivit*’ OR ’wheat allerg*’ OR ’nut hypersensitivit*’ OR ’nut allerg*’ OR ’peanut hypersensitivit*’ OR ’peanut allerg*’ OR ’groundnut hypersensitivit*’ OR ’groundnut allerg*’
8	(MH ’Rhinitis, Allergic+’) OR (MH ’Rhinitis, Allergic, Seasonal+’) OR (MH ’Rhinitis, Allergic, Perennial+’) OR (MH ’Rhinitis+’) OR (MH ’Conjunctivitis, Allergic+’) OR (MH ’Conjunctivitis+’) OR ’allergic rhinoconjunctiviti*’ OR ’rhinoconjunctiviti*’ OR ’allergic rhiniti*’ OR ’seasonal allergic rhiniti*’ OR ’perennial allergic rhiniti*’ OR ’rhiniti*’ OR ’allergic conjunctiviti*’ OR ’vernal keratoconjunctiviti*’ OR ’vernal conjunctiviti*’ OR ’giant papillary conjunctiviti*’ OR ’hay fever’ OR ’hayfever’ OR ’pollinosis’ OR ’pollenosis’ OR ’nasal catarrh’
9	1 OR 2 OR 3
10	4 OR 5 OR 6 OR 7 OR 8
11	9 AND 10

Full query

((MH "Birth Order+") OR (MH "Parity+") OR "birth order*" OR "birth rank*" OR "multiple birth*" OR "parity" OR (MH "Family Characteristics+") OR (MH "Family Health+") OR "family charactersitic*" OR "family size*" OR "family structure*" OR "family demograph*" OR "family composition" OR "household size*" OR "household demograph*" OR "household composition" OR (MH "Siblings+") OR (MH "Sibling Relations+") OR "sibling*" OR "sister*" OR "brother*" OR "sibship size*" OR "sibship*") AND ((MH "Asthma+") OR (MH "Respiratory Hypersensitivity+") OR "bronchial asthma*" OR "exercise-induced asthma" OR "asthma*" OR "exercise-induced bronchospasm" OR "respiratory hypersensitivit*" OR ‘airway hyper responsiveness" OR "airway hyper-responsiveness" OR "respiratory hyper responsiveness" OR "respiratory hyper-responsiveness" OR "wheez*" OR (MH "Hypersensitivity+") OR (MH "Hypersensitivity, Immediate+") OR (MH "Hypersensitivity, Delayed+") OR (MH "Allergy and Immunology+") OR "immedate hypersensitivit*" OR "delayed hypersensitivit*" OR "IgE-mediated hypersensitivit*" OR "type I hypersensitivit*" OR "type IV hypersensitivit*" OR "hypersensitivit*" OR "atopic sensitiziation" OR "atop*" OR "allergic sensitization" OR "allergic disease* OR "allerg*" OR (MH "Dermatitis, Atopic+") OR (MH "Eczema+") OR (MH ‘Angioedema+") OR (MH ‘Anaphylaxis+") OR (MH "Urticaria+") OR "atopic dermatitis" OR "dermatitis" OR "atopic eczema" OR "eczema" OR "nerudoarmatiti*" OR "besnier's prurigo" OR "besniers prurigo" OR "besnier prurigo" OR "urticari*" OR "hives" OR "anaphyla*" OR "quincke's edema" OR "quinckes edema" OR "quincke edema" OR "angioneurotic edema" OR "angioedema" OR (MH "Food Hypersensitivity+") OR "food hypersensitivit*" OR "food allerg*" OR "egg hypersensitivit*" OR "egg allerg*" OR "milk hypersensitivit*" OR "milk allerg*" OR "shellfish hypersensitivit*" OR "shellfish allerg*" OR "wheat hypersensitivit*" OR "wheat allerg*" OR "nut hypersensitivit*" OR "nut allerg*" OR "peanut hypersensitivit*" OR "peanut allerg*" OR "groundnut hypersensitivit*" OR "groundnut allerg*" OR (MH "Rhinitis, Allergic+") OR (MH "Rhinitis, Allergic, Seasonal+") OR (MH "Rhinitis, Allergic, Perennial+") OR (MH "Rhinitis+") OR (MH "Conjunctivitis, Allergic+") OR (MH "Conjunctivitis+") OR "allergic rhinoconjunctiviti*" OR "rhinoconjunctiviti*" OR

Lisik D, *et al. BMJ Open* 2022; 11:e045795. doi: 10.1136/bmjopen-2020-045795

"allergic rhiniti*" OR "seasonal allergic rhiniti*" OR "perennial allergic rhiniti*" OR "rhiniti*" OR "allergic conjunctiviti*" OR "vernal keratoconjunctiviti*" OR "vernal conjunctiviti*" OR "giant papillary conjunctiviti*" OR "hay fever" OR "hayfever" OR "pollinosis" OR "pollenosis" OR "nasal catarrh"))

MH = subject heading

Embase

#	**Search term(s)**
1	sibship.mp.
2	birth order.mp. or exp birth order/
3	birth rank.mp.
4	multiple birth.mp.
5	parity.mp. or exp parity/
6	family characteristic.mp. or exp family size/
7	family structure.mp.
8	family demograph.mp.
9	family demograph*.mp.
10	family composition.mp.
11	household size.mp.
12	household demograph*.mp.
13	household composition.mp.
14	exp sibling relation/ or sibling.mp. or sibling/
15	exp sister/ or sister.mp.
16	siblings.mp.
17	sisters.mp.
18	brother.mp. or exp brother/
19	brothers.mp.
20	or/1-19
21	exp Asthma/ or (bronchial asthma* or exercise-induced asthma* or exercise-induced bronchospasm* or asthma* or respiratory hypersensitivit* or airway hyper responsiveness or airway hyper-responsiveness or respiratory hyper responsiveness or respiratory hyper-responsiveness or wheez*).mp.
22	exp Hypersensitivity/ or exp Hypersensitivity Immediate/ or exp Hypersensitivity Delayed/ or (immediate hypersensitivit* or delayed hypersensitivit* or IgE-mediated hypersensitivit* or type I hypersensitivit* or type IV hypersensitivit* or atopic sensitization or atop* or allergic sensitization or allerg*).mp.
23	exp Dermatitis/ or exp Anaphylaxis/ or (atopic dermatitis or dermatitis or neurodermatiti* or besnier prurigo or besniers prurigo or besnier prurigo or atopic eczema or eczema or urticari* or anaphyla* or quincke edema or quinckes edema or quincke edema or angioneurotic edema or angioedema or hives).mp.

Lisik D, *et al. BMJ Open* 2022; 11:e045795. doi: 10.1136/bmjopen-2020-045795

24	exp Food Hypersensitivity/ or (food hypersensitivit* or food allerg* or egg hypersensitivit* or egg allerg* or milk hypersensitivit* or milk allerg* or shellfish hypersensitivit* or shellfish allerg* or wheat hypersensitivit* or wheat allerg* or nut hypersensitivit* or nut allerg* or peanut hypersensitivit* or peanut allerg* or groundnut hypersensitivit* or groundnut allerg*).mp.
25	exp Rhinitis/ or exp Conjunctivitis/ or (allergic rhinoconjunctiviti* or rhinoconjunctiviti* or allergi rhiniti* or seasonal allergic rhiniti* or perennial allergic rhiniti* or rhiniti* or allergic conjunctiviti* or vernal keratoconjunctiviti* or vernal conjunctiviti* or giant papillary conjunctiviti* or hay fever or hayfever or pollinosis or pollinosis or nasal catarrh*).mp.
26	or/21-25
27	20 and 26

Google Scholar

#	**Search term(s)**
1	"family size" OR "family structure" OR "household size"
2	"sibling" OR "sibship"
3	"asthma" OR "wheezing"
4	"atopy" OR "allergy"
5	"eczema" OR "urticaria" OR "angioedema" OR "anaphylaxis"
6	"rhinitis" OR "allergic conjunctivitis" OR "hay fever"
7	1 OR 2
8	3 OR 4 OR 5 OR 6
9	7 AND 8
Full query ("family size" OR "family structure" OR "household size" OR "sibling" OR "sibship") AND ("asthma" OR "wheezing" OR "atopy" OR "allergy" OR "anaphylaxis" OR "eczema" OR "urticaria" OR "angioedema" OR "rhinitis" OR "allergic conjunctivitis" OR "hay fever")	

PsycINFO

#	**Search term(s)**
1	SU.EXACT.EXPLODE("Birth Order") OR TI,AB(”birth order*”) OR TI,AB(”multiple birth*”) OR TI,AB(”birth rank*”) OR TI,AB(”parity”)
2	SU.EXACT.EXPLODE("Family Structure") OR SU.EXACT.EXPLODE("Family Size") OR TI,AB(”family characteristic*”) OR TI,AB(”family size*”) OR TI,AB(”family structure*”) OR TI,AB(”family demograph*”) OR TI,AB(”family composition”) OR TI,AB(”household size*”) OR TI,AB(”household demograph*”) OR TI,AB(”household composition”)
3	SU.EXACT.EXPLODE("Siblings") OR SU.EXACT.EXPLODE("Sibling Relations") OR TI,AB(”sibling*’”) OR TI,AB(”sister*”) OR TI,AB(”brother*”) OR TI,AB(”sibship size*”) OR TI,AB(”sibship size*”) OR TI,AB(”sibship*”)

4	SU.EXACT.EXPLODE("Asthma") OR TI,AB(”bronchial asthma*”) OR TI,AB(”exercise-induced asthma*”) OR TI,AB(”asthma*”) OR TI,AB(”exercise-induced bronchospasm*”) OR TI,AB(”respiratory hypersensitivit*”) OR TI,AB(”airway hyper responsiveness”) OR TI,AB(”airway hyper-responsiveness”) OR TI,AB(”respiratory hyper responsiveness”) OR TI,AB(”respiratory hyper-responsiveness”) OR TI,AB(”wheez*”)
5	SU.EXACT.EXPLODE("Allergic Disorders") OR TI,AB(”immediate hypersensitivit*”) OR TI,AB(”delayed hypersensitivit*”) OR TI,AB(”hypersensitivit*”) OR TI,AB(”IgE-mediated hypersensitivit*”) OR TI,AB(”type I hypersensitivit*”) OR TI,AB(”type IV hypersensitivit*”) OR TI,AB(”atopic sensitization”) OR TI,AB(”atop*”) OR TI,AB(”allergic sensitization”) OR TI,AB(”allergic disease*”) OR TI,AB(”allerg*”)
6	SU.EXACT.EXPLODE("Allergic Skin Disorders") OR SU.EXACT.EXPLODE("Neurodermatitis") OR SU.EXACT.EXPLODE("Dermatitis") OR SU.EXACT.EXPLODE("Eczema") OR SU.EXACT.EXPLODE("Anaphylactic Shock") OR TI,AB(”atopic dermatitis”) OR TI,AB(”dermatitis”) OR TI,AB(”atopic eczema”) OR TI,AB(”eczema”) OR TI,AB(”neurodermatiti*”) OR TI,AB(”besnier’s prurigo”) OR TI,AB(”besniers prurigo”) OR TI,AB(”besnier prurigo”) OR TI,AB(”urticari*”) OR TI,AB(”hives”) OR TI,AB(”anaphyla*”) OR TI,AB(”quincke’s edema”) OR TI,AB(”quinckes edema”) OR TI,AB(”quincke edema”) OR TI,AB(”angioneurotic edema”) OR TI,AB(”angioedema”)
7	SU.EXACT.EXPLODE("Food Allergies") OR TI,AB(”food hypersensitivit*”) OR TI,AB(”food allerg*”) OR TI,AB(”egg hypersensitivit*”) OR TI,AB(”egg allerg*”) OR TI,AB(”milk hypersensitivit*”) OR TI,AB(”milk allerg*”) OR TI,AB(”shellfish hypersensitivit*”) OR TI,AB(”shellfish allerg*”) OR TI,AB(”wheat hypersensitivit*”) OR TI,AB(”wheat allerg*”) OR TI,AB(”nut hypersensitivit*”) OR TI,AB(”nut allerg*”) OR TI,AB(”peanut hypersensitivit*”) OR TI,AB(”peanut allerg*”) OR TI,AB(”groundnut hypersensitivit*”) OR TI,AB(”groundnut allerg*”)
8	TI,AB(”allergic rhinoconjunctiviti*”) OR TI,AB(”rhinoconjunctiviti*”) OR TI,AB(”allergic rhiniti*”) OR TI,AB(”rhiniti*”) OR TI,AB(”seasonal allergic rhiniti*”) OR TI,AB(”perennial allergic rhiniti*”) OR TI,AB(”allergic conjunctiviti*”) OR TI,AB(”vernal keratoconjunctiviti*”) OR TI,AB(”vernal conjunctiviti*”) OR TI,AB(”giant papillary conjunctiviti*”) OR TI,AB(”hay fever”) OR TI,AB(”hayfever”) OR TI,AB(“pollinosis”) OR TI,AB(”pollenosis”) OR TI,AB(”nasal catarrh*”)
9	1 OR 2 OR 3
10	4 OR 5 OR 6 OR 7 OR 8
11	9 AND 10
Full query<br>(SU.EXACT.EXPLODE("Birth Order") OR TI,AB("birth order*") OR TI,AB("multiple birth*") OR TI,AB("birth rank*") OR TI,AB("parity") OR SU.EXACT.EXPLODE("Family Structure") OR SU.EXACT.EXPLODE("Family Size") OR TI,AB("family characteristic*") OR TI,AB("family size*") OR TI,AB("family structure*") OR TI,AB("family demograph*") OR TI,AB("family composition") OR TI,AB("household size*") OR TI,AB("household demograph*") OR TI,AB("household composition") OR SU.EXACT.EXPLODE("Siblings") OR SU.EXACT.EXPLODE("Sibling Relations") OR TI,AB("sibling*") OR TI,AB("sister*") OR TI,AB("brother*") OR	

TI,AB("sibship size*") OR TI,AB("sibship size*") OR TI,AB("sibship*")) AND (SU.EXACT.EXPLODE("Asthma") OR TI,AB("bronchial asthma*") OR TI,AB("exercise-induced asthma*") OR TI,AB("asthma*") OR TI,AB("exercise-induced bronchospasm*") OR TI,AB("respiratory hypersensitivit*") OR TI,AB("airway hyper responsiveness") OR TI,AB("airway hyper-responsiveness") OR TI,AB("respiratory hyper responsiveness") OR TI,AB("respiratory hyper-responsiveness") OR TI,AB("wheez*") OR SU.EXACT.EXPLODE("Allergic Disorders") OR TI,AB("immediate hypersensitivit*") OR TI,AB("delayed hypersensitivit*") OR TI,AB("hypersensitivit*") OR TI,AB("IgE-mediated hypersensitivit*") OR TI,AB("type I hypersensitivit*") OR TI,AB("type IV hypersensitivit") OR TI,AB("atopic sensitization") OR TI,AB("atop*") OR TI,AB("allergic sensitization") OR TI,AB("allergic disease*") OR TI,AB("allerg*") OR SU.EXACT.EXPLODE("Allergic Skin Disorders") OR SU.EXACT.EXPLODE("Neurodermatitis") OR SU.EXACT.EXPLODE("Dermatitis") OR SU.EXACT.EXPLODE("Eczema") OR SU.EXACT.EXPLODE("Anaphylactic Shock") OR TI,AB("atopic dermatitis") OR TI,AB("dermatitis") OR TI,AB("atopic eczema") OR TI,AB("eczema") OR TI,AB("neurodermatiti*") OR TI,AB("besnier’s prurigo") OR TI,AB("besniers prurigo") OR TI,AB("besnier prurigo") OR TI,AB("urticari*") OR TI,AB("hives") OR TI,AB("anaphyla*") OR TI,AB("quincke’s edema") OR TI,AB("quinckes edema") OR TI,AB("quincke edema") OR TI,AB("angioneurotic edema") OR TI,AB("angioedema") OR SU.EXACT.EXPLODE("Food Allergies") OR TI,AB("food hypersensitivit*") OR TI,AB("food allerg*") OR TI,AB("egg hypersensitivit*") OR TI,AB("egg allerg*") OR TI,AB("milk hypersensitivit*") OR TI,AB("milk allerg*") OR TI,AB("shellfish hypersensitivit*") OR TI,AB("shellfish allerg*") OR TI,AB("wheat hypersensitivit*") OR TI,AB("wheat allerg*") OR TI,AB("nut hypersensitivit*") OR TI,AB("nut allerg*") OR TI,AB("peanut hypersensitivit*") OR TI,AB("peanut allerg*") OR TI,AB("groundnut hypersensitivit*") OR TI,AB("groundnut allerg*") OR TI,AB("allergic rhinoconjunctiviti*") OR TI,AB("rhinoconjunctiviti*") OR TI,AB("allergic rhiniti*") OR TI,AB("rhiniti*") OR TI,AB("seasonal allergic rhiniti*") OR TI,AB("perennial allergic rhiniti*") OR TI,AB("allergic conjunctiviti*") OR TI,AB("vernal keratoconjunctiviti*") OR TI,AB("vernal conjunctiviti*") OR TI,AB("giant papillary conjunctiviti*") OR TI,AB("hay fever") OR TI,AB("hayfever") OR TI,AB(“pollinosis") OR TI,AB("pollenosis") OR TI,AB("nasal catarrh*"))

SU = all subjects and indexing; TI,AB = title, abstract

PubMed

#	**Search term(s)**
1	Birth Order[mh] OR Parity[mh] OR birth order*[tiab] OR multiple birth*[tiab] OR birth rank*[tiab] OR parity[tiab]
2	Family Characteristics[mh] OR Family Health[mh] OR family characteristic*[tiab] OR family size*[tiab] OR family structure*[tiab] OR family demograph*[tiab] OR family composition[tiab] OR household size*[tiab] OR household demograph*[tiab] OR household composition[tiab]
3	Siblings[mh] OR Sibling Relations[mh] OR sibling*[tiab] OR sister*[tiab] OR brother*[tiab] OR sibship size*[tiab] OR sibship*[tiab]
4	Asthma[mh] OR Asthma, Exercise-Induced[mh] OR Respiratory Hypersensitivity[mh] OR bronchial asthma*[tiab] OR exercise-induced asthma*[tiab] OR exercise-induced bronchospasm*[tiab] OR asthma*[tiab] OR respiratory hypersensitivit*[tiab] OR airway hyper responsiveness[tiab] OR airway hyper-responsiveness[tiab] OR respiratory hyper responsiveness[tiab] OR respiratory hyper-responsiveness[tiab] OR wheez*[tiab]

Lisik D, *et al. BMJ Open* 2022; 11:e045795. doi: 10.1136/bmjopen-2020-045795

5	Hypersensitivity[mh] OR Hypersensitivity, Immediate[mh] OR Hypersensitivity, Delayed[mh] OR Allergy and Immunology[mh] OR Allergens / Immunology[mh] OR immediate hypersensitivit*[tiab] OR delayed hypersensitivit*[tiab] OR hypersensitivit*[tiab] OR IgE-mediated hypersensitivit*[tiab] OR type I hypersensitivit*[tiab] OR type IV hypersensitivit*[tiab] OR atopic sensitization[tiab] OR atop*[tiab] OR allergic sensitization[tiab] OR allergic disease*[tiab] OR allerg*[tiab]
6	Dermatitis, Atopic[mh] OR Eczema[mh] OR Angioedema[mh] OR Anaphylaxis[mh] OR Urticaria[mh] OR atopic dermatitis[tiab] OR dermatitis[tiab] OR atopic eczema[tiab] OR eczema[tiab] OR neurodermatiti*[tiab] OR besnier’s prurigo[tiab] OR besniers prurigo[tiab] OR besnier prurigo[tiab] OR urticari*[tiab] OR anaphyla*[tiab] OR quincke edema[tiab] OR quinckes edema[tiab] OR quincke’s edema[tiab] OR angioneurotic edema[tiab] OR angioedema[tiab] OR hives[tiab]
7	Food Hypersensitivity[mh] OR food hypersensitivit*[tiab] OR food allerg*[tiab] OR egg allerg*[tiab] OR egg hypersensitivit*[tiab] OR milk allerg*[tiab] OR milk hypersensitivit*[tiab] OR shellfish allerg*[tiab] OR shellfish hypersensitivit*[tiab] OR wheat allerg*[tiab] OR wheat hypersensitivit*[tiab] OR nut allerg*[tiab] OR nut hypersensitivit*[tiab] OR peanut allerg*[tiab] OR peanut hypersensitivit*[tiab] OR groundnut hypersensitivit*[tiab]
8	Pollen / Immunology[mh] OR Rhinitis, Allergic[mh] OR Rhinitis, Allergic, Seasonal[mh] OR Rhinitis, Allergic, Perennial[mh] OR Rhinitis[mh] OR Conjunctivitis, Allergic[mh] OR Conjunctivitis / Immunology[mh] OR Conjunctivitis / Epidemiology[mh] OR Conjunctivitis / Etiology[mh] OR allergic rhinoconjunctiviti*[tiab] OR rhinoconjunctiviti*[tiab] OR allergic rhiniti*[tiab] OR rhiniti*[tiab] OR seasonal allergic rhiniti*[tiab] OR perennial allergic rhiniti*[tiab] OR allergic conjunctiviti*[tiab] OR vernal keratoconjunctiviti*[tiab] OR vernal conjunctiviti*[tiab] OR giant papillary conjunctiviti*[tiab] OR hay fever[tiab] OR hayfever[tiab] OR pollinosis[tiab] OR pollenosis[tiab] OR nasal catarrh*[tiab]
9	1 OR 2 OR 3
10	4 OR 5 OR 6 OR 7 OR 8
11	9 AND 10

Full query

(Birth Order[mh] OR Parity[mh] OR birth order*[tiab] OR multiple birth*[tiab] OR birth rank*[tiab] OR parity[tiab] OR Family Characteristics[mh] OR Family Health[mh] OR family characteristic*[tiab] OR family size*[tiab] OR family structure*[tiab] OR family demograph*[tiab] OR family composition[tiab] OR household size*[tiab] OR household demograph*[tiab] OR household composition[tiab] OR Siblings[mh] OR Sibling Relations[mh] OR sibling*[tiab] OR sister*[tiab] OR brother*[tiab] OR sibship size*[tiab] OR sibship*[tiab]) AND (Asthma[mh] OR Asthma, Exercise-Induced[mh] OR Respiratory Hypersensitivity[mh] OR bronchial asthma*[tiab] OR exercise-induced asthma*[tiab] OR exercise-induced bronchospasm*[tiab] OR asthma*[tiab] OR respiratory hypersensitivit*[tiab] OR airway hyper responsiveness[tiab] OR airway hyper-responsiveness[tiab] OR respiratory hyper responsiveness[tiab] OR respiratory hyper-responsiveness[tiab] OR wheez*[tiab] OR Hypersensitivity[mh] OR Hypersensitivity, Immediate[mh] OR Hypersensitivity, Delayed[mh] OR Allergy and Immunology[mh] OR Allergens / Immunology[mh] OR immediate hypersensitivit*[tiab] OR delayed hypersensitivit*[tiab] OR hypersensitivit*[tiab] OR IgE-mediated hypersensitivit*[tiab] OR type I hypersensitivit*[tiab] OR type IV hypersensitivit*[tiab] OR atopic sensitization[tiab] OR atop*[tiab] OR allergic sensitization[tiab] OR allergic disease*[tiab] OR allerg*[tiab] OR Dermatitis, Atopic[mh] OR Eczema[mh] OR Angioedema[mh] OR Anaphylaxis[mh] OR Urticaria[mh] OR atopic dermatitis[tiab] OR dermatitis[tiab] OR atopic eczema[tiab] OR eczema[tiab] OR neurodermatiti*[tiab]OR besnier’s prurigo[tiab] OR besniers prurigo[tiab] OR besnier prurigo[tiab] OR urticari*[tiab] OR anaphyla*[tiab] OR quincke edema[tiab] OR quinckes edema[tiab] OR quincke’s edema[tiab] OR angioneurotic edema[tiab] OR angioedema[tiab] OR hives[tiab]

Lisik D, *et al. BMJ Open* 2022; 11:e045795. doi: 10.1136/bmjopen-2020-045795

OR Food Hypersensitivity[mh] OR food hypersensitivit*[tiab] OR food allerg*[tiab] OR egg allerg*[tiab] OR egg hypersensitivit*[tiab] OR milk allerg*[tiab] OR milk hypersensitivit*[tiab] OR shellfish allerg*[tiab] OR shellfish hypersensitivit*[tiab] OR wheat allerg*[tiab] OR wheat hypersensitivit*[tiab] OR nut allerg*[tiab] OR nut hypersensitivit*[tiab] OR peanut allerg*[tiab] OR peanut hypersensitivit*[tiab] OR groundnut hypersensitivit*[tiab] OR Pollen / Immunology[mh] OR Rhinitis, Allergic[mh] OR Rhinitis, Allergic, Seasonal[mh] OR Rhinitis, Allergic, Perennial[mh] OR Rhinitis[mh] OR Conjunctivitis, Allergic[mh] OR Conjunctivitis / Immunology[mh] OR Conjunctivitis / Epidemiology[mh] OR Conjunctivitis / Etiology[mh] OR allergic rhinoconjunctiviti*[tiab] OR rhinoconjunctiviti*[tiab] OR allergic rhiniti*[tiab] OR rhiniti*[tiab] OR seasonal allergic rhiniti*[tiab] OR perennial allergic rhiniti*[tiab] OR allergic conjunctiviti*[tiab] OR vernal keratoconjunctivitis*[tiab] OR vernal conjunctiviti*[tiab] OR giant papillary conjunctiviti*[tiab] OR hay fever[tiab] OR hayfever[tiab] OR pollinosis[tiab] OR pollenosis[tiab] OR nasal catarrh*[tiab])

mh = MeSH; tiab = title, abstract

Scopus

#	Search term(s)
1	TITLE-ABS-KEY(”birth order*”) OR TITLE-ABS-KEY(”multiple birth*”) OR TITLE-ABS-KEY(”birth rank*”) OR TITLE-ABS-KEY(”parity”)
2	TITLE-ABS-KEY(”family characteristic*”) OR TITLE-ABS-KEY(”family size*”) OR TITLE-ABS-KEY(”family structure*”) OR TITLE-ABS-KEY(”family demograph*”) OR TITLE-ABS-KEY(”family composition”) OR TITLE-ABS-KEY(”household size*”) OR TITLE-ABS-KEY(”household demograph*”) OR TITLE-ABS-KEY(”household composition*”)
3	TITLE-ABS-KEY(”sibling relation*”) OR TITLE-ABS-KEY(”sibling*”) OR TITLE-ABS-KEY(”brother*”) OR TITLE-ABS-KEY(”sister*”) OR TITLE-ABS-KEY(”sibship size*”) OR TITLE-ABS-KEY(”sibship*”)
4	TITLE-ABS-KEY(”bronchial asthma*”) OR TITLE-ABS-KEY(”exercise-induced asthma*”) OR TITLE-ABS-KEY(”asthma*”) OR TITLE-ABS-KEY(”exercise-induced bronchospasm*”) OR TITLE-ABS-KEY(”respiratory hypersensitivit*”) OR TITLE-ABS-KEY(”airway hyper responsiveness”) OR TITLE-ABS-KEY(”airway hyper-responsiveness”) OR TITLE-ABS-KEY(”respiratory hyper responsiveness”) OR TITLE-ABS-KEY(”respiratory hyper-responsiveness”) OR TITLE-ABS-KEY(”wheez*”)
5	TITLE-ABS-KEY(”immediate hypersensitivit*”) OR TITLE-ABS-KEY(”delayed hypersensitivit*”) OR TITLE-ABS-KEY(”IgE-mediated hypersensitivit*”) OR TITLE-ABS-KEY(”type I hypersensitivit*”) OR TITLE-ABS-KEY(”type IV hypersensitivit*”) OR TITLE-ABS-KEY(”atopic sensitization”) OR TITLE-ABS-KEY(”atop*”) OR TITLE-ABS-KEY(”allergic sensitization”) OR TITLE-ABS-KEY(”allergic disease”) OR TITLE-ABS-KEY(”allerg*”)
6	TITLE-ABS-KEY(”atopic dermatitis”) OR TITLE-ABS-KEY(”dermatitis”) OR TITLE-ABS-KEY(”atopic eczema”) OR TITLE-ABS-KEY(”eczema”) OR TITLE-ABS-KEY(”neurodermatitis*”) OR TITLE-ABS-KEY(”besnier’s prurigo”) OR TITLE-ABS-KEY(”besniers prurigo”) OR TITLE-ABS-KEY(”besnier prurigo”) OR TITLE-ABS-KEY(”quincke’s edema”) OR TITLE-ABS-KEY(”quinckes edema”) OR TITLE-ABS-KEY(”quincke edema”) OR TITLE-ABS-KEY(”angioneurotic edema”) OR TITLE-ABS-KEY(”hives”) OR TITLE-ABS-KEY(”anaphyla*”) OR TITLE-ABS-KEY(”urticari*”)

7	TITLE-ABS-KEY("food hypersensitivit*") OR TITLE-ABS-KEY("food allerg*") OR TITLE-ABS-KEY("egg allerg*") OR TITLE-ABS-KEY("egg hypersensitivit*") OR TITLE-ABS-KEY("milk allerg*") OR TITLE-ABS-KEY("milk hypersensitivit*") OR TITLE-ABS-KEY("shellfish allerg*") OR TITLE-ABS-KEY("shellfish hypersensitivit*") OR TITLE-ABS-KEY("wheat allerg*") OR TITLE-ABS-KEY("wheat hypersensitivit*") OR TITLE-ABS-KEY("nut allerg*") OR TITLE-ABS-KEY("nut hypersensitivit*") OR TITLE-ABS-KEY("peanut allerg*") OR TITLE-ABS-KEY("peanut hypersensitivit*") OR TITLE-ABS-KEY("groundnut allerg*") OR TITLE-ABS-KEY("groundnut hypersensitivit*")
8	TITLE-ABS-KEY("allergic rhinoconjunctiviti*") OR TITLE-ABS-KEY("rhinoconjunctiviti*") OR TITLE-ABS-KEY("seasonal allergic rhiniti*") OR TITLE-ABS-KEY("perennial allergic rhiniti*") OR TITLE-ABS-KEY("allergic rhiniti*") OR TITLE-ABS-KEY("rhiniti*") OR TITLE-ABS-KEY("allergic conjunctiviti*") OR TITLE-ABS-KEY("vernal keratoconjunctiviti*") OR TITLE-ABS-KEY("vernal conjunctiviti*") OR TITLE-ABS-KEY("giant papillary conjunctiviti*") OR TITLE-ABS-KEY("hay fever") OR TITLE-ABS-KEY("hayfever") OR TITLE-ABS-KEY("pollinosis") OR TITLE-ABS-KEY("pollenosis") OR TITLE-ABS-KEY("nasal catarrh*")
9	1 OR 2 OR 3
10	4 OR 5 OR 6 OR 7 OR 8
11	9 AND 10

Full query

(TITLE-ABS-KEY("birth order*") OR TITLE-ABS-KEY("multiple birth*") OR TITLE-ABS-KEY("birth rank*") OR TITLE-ABS-KEY("parity") OR TITLE-ABS-KEY("family characteristic*") OR TITLE-ABS-KEY("family size*") OR TITLE-ABS-KEY("family structure*") OR TITLE-ABS-KEY("family demograph*") OR TITLE-ABS-KEY("family composition") OR TITLE-ABS-KEY("household size*") OR TITLE-ABS-KEY("household demograph*") OR TITLE-ABS-KEY("household composition*") OR TITLE-ABS-KEY("sibling relation*") OR TITLE-ABS-KEY("sibling*") OR TITLE-ABS-KEY("brother*") OR TITLE-ABS-KEY("sister*") OR TITLE-ABS-KEY("sibship size*") OR TITLE-ABS-KEY("sibship*")) AND (TITLE-ABS-KEY("bronchial asthma*") OR TITLE-ABS-KEY("exercise-induced asthma*") OR TITLE-ABS-KEY("asthma*") OR TITLE-ABS-KEY("exercise-induced bronchospasm*") OR TITLE-ABS-KEY("respiratory hypersensitivit*") OR TITLE-ABS-KEY("airway hyper responsiveness") OR TITLE-ABS-KEY("airway hyper-responsiveness") OR TITLE-ABS-KEY("respiratory hyper responsiveness") OR TITLE-ABS-KEY("respiratory hyper-responsiveness") OR TITLE-ABS-KEY("wheez*") OR TITLE-ABS-KEY("immediate hypersensitivit*") OR TITLE-ABS-KEY("delayed hypersensitivit*") OR TITLE-ABS-KEY("IgE-mediated hypersensitivit*") OR TITLE-ABS-KEY("type I hypersensitivit*") OR TITLE-ABS-KEY("type IV hypersensitivit*") OR TITLE-ABS-KEY("atopic sensitization") OR TITLE-ABS-KEY("atop*") OR TITLE-ABS-KEY("allergic sensitization") OR TITLE-ABS-KEY("allergic disease") OR TITLE-ABS-KEY("allerg*") OR TITLE-ABS-KEY("atopic dermatitis") OR TITLE-ABS-KEY("dermatitis") OR TITLE-ABS-KEY("atopic eczema") OR TITLE-ABS-KEY("eczema") OR TITLE-ABS-KEY("neurodermatitis*") OR TITLE-ABS-KEY("besnier's prurigo") OR TITLE-ABS-KEY("besniers prurigo") OR TITLE-ABS-KEY("besnier prurigo") OR TITLE-ABS-KEY("quincke's edema") OR TITLE-ABS-KEY("quinckes edema") OR TITLE-ABS-KEY("quincke edema") OR TITLE-ABS-KEY("angioneurotic edema") OR TITLE-ABS-KEY("hives") OR TITLE-ABS-KEY("anaphyla*") OR TITLE-ABS-KEY("urticari*") OR TITLE-ABS-KEY("food hypersensitivit*") OR TITLE-ABS-KEY("food allerg*") OR TITLE-ABS-KEY("egg allerg*") OR TITLE-ABS-KEY("egg hypersensitivit*") OR TITLE-ABS-KEY("milk allerg*") OR TITLE-ABS-KEY("milk hypersensitivit*") OR TITLE-ABS-KEY("shellfish allerg*") OR TITLE-ABS-KEY("shellfish

hypersensitivit*") OR TITLE-ABS-KEY("wheat allerg*") OR TITLE-ABS-KEY("wheat hypersensitivit*") OR TITLE-ABS-KEY("nut allerg*") OR TITLE-ABS-KEY("nut hypersensitivit*") OR TITLE-ABS-KEY("peanut allerg*") OR TITLE-ABS-KEY("peanut hypersensitivit*") OR TITLE-ABS-KEY("groundnut allerg*") OR TITLE-ABS-KEY("groundnut hypersensitivit*") OR TITLE-ABS-KEY("allergic rhinoconjunctiviti*") OR TITLE-ABS-KEY("rhinoconjunctiviti*") OR TITLE-ABS-KEY("seasonal allergic rhiniti*") OR TITLE-ABS-KEY("perennial allergic rhiniti*") OR TITLE-ABS-KEY("allergic rhiniti*") OR TITLE-ABS-KEY("rhiniti*") OR TITLE-ABS-KEY("allergic conjunctiviti*") OR TITLE-ABS-KEY("vernal keratoconjunctiviti*") OR TITLE-ABS-KEY("vernal conjunctiviti*") OR TITLE-ABS-KEY("giant papillary conjunctiviti*") OR TITLE-ABS-KEY("hay fever") OR TITLE-ABS-KEY("hayfever") OR TITLE-ABS-KEY("pollinosis") OR TITLE-ABS-KEY("pollenosis") OR TITLE-ABS-KEY("nasal catarrh*"))

TITLE-ABS-KEY = title, abstract, keywords

Web of Science

#	**Search term(s)**
1	TS="birth order*" OR TS="multiple birth*" OR TS="birth rank*" OR TS="parity"
2	TS="family characteristic*" OR TS="family size*" OR TS="family structure*" OR TS="family demograph*" OR TS="family composition" OR TS="household size*" OR TS="household demograph*" OR TS="household composition"
3	TS="sibling*" OR TS="sister*" OR TS="brother*" OR TS="sibship size*" OR TS="sibship*"
4	TS="bronchial asthma*" OR TS="exercise-induced asthma*" OR TS="exercise-induced bronchospasm*" OR TS="asthma*" OR TS="respiratory hypersensitivit*" OR TS="respiratory hyper-responsiveness*" OR TS="airway hyper responsiveness*" OR TS="airway hyper-responsiveness*" OR TS=wheez*
5	TS="immediate hypersensitivit*" OR TS="delayed hypersensitivit*" OR TS="IgE-mediated hypersensitivit*" OR TS="type I hypersensitivit*" OR TS="type IV hypersensitivit*" OR TS="hypersensitivit*" OR TS="atopic sensitization" OR TS="atop*" OR TS="allergic sensitization" OR TS="allergic disease*" OR TS="allerg*"
6	TS="atopic dermatitis" OR TS="dermatitis" OR TS="atopic eczema" OR TS="eczema" OR TS="neurodermatiti*" OR TS="besnier's prurigo" OR TS="besniers prurigo" OR TS="besnier prurigo" OR TS="urticari*" OR TS="anaphyla*" OR TS="quincke's edema" OR TS="quinckes edema" OR TS="quincke edema" OR TS="angioneurotic edema" OR TS="angioedema" OR TS="hives"
7	TS="food hypersensitivit*" OR TS="food allerg*" OR TS="egg allerg*" OR TS="egg hypersensitivit*" OR TS="milk allerg*" OR TS="milk hypersensitivit*" OR TS="shellfish allerg*" OR TS="shellfish hypersensitivit*" OR TS="wheat allerg*" OR TS="wheat hypersensitivit*" OR TS="nut allerg*" OR TS="nut hypersensitivit*" OR TS="peanut allerg*" OR TS="peanut hypersensitivit*" OR TS="groundnut allerg*" OR TS="groundnut hypersensitivit*"
8	TS="pollen allerg*" OR TS="allergic rhinoconjunctiviti*" OR TS="rhinoconjunctiviti*" OR TS="seasonal allergic rhiniti*" OR TS="perennial allergic rhiniti*" OR TS="allergic rhiniti*" OR TS="rhiniti*" OR TS="allergic conjunctiviti*" OR TS="vernal keratoconjunctiviti*" OR

	TS=”vernal conjunctiviti*” OR TS=”giant papillary conjunctiviti*” OR TS=”hay fever” OR TS=”hayfever” OR TS=”pollinosis” OR TS=”pollenosis” OR TS=”nasal catarrh*”
9	1 OR 2 OR 3
10	4 OR 5 OR 6 OR 7 OR 8
11	9 AND 10

Full query

(TS="birth order*" OR TS="multiple birth*" OR TS="birth rank*" OR TS="parity" OR TS="family characteristic*" OR TS="family size*" OR TS="family structure*" OR TS="family demograph*" OR TS="family composition" OR TS="household size*" OR TS="household demograph*" OR TS="household composition" OR TS="sibling*" OR TS="sister*" OR TS="brother*" OR TS="sibship size*" OR TS="sibship*") AND (TS="bronchial asthma*" OR TS="exercise-induced asthma*" OR TS="exercise-induced bronchospasm*" OR TS="asthma*" OR TS=“respiratory hypersensitivit*" OR TS=“respiratory hyper-responsiveness*" OR TS=“airway hyper responsiveness*" OR TS=“airway hyper-responsiveness*" OR TS=wheez* OR TS="immediate hypersensitivit*" OR TS="delayed hypersensitivit*" OR TS="IgE-mediated hypersensitivit*" OR TS="type I hypersensitivit*" OR TS="type IV hypersensitivit*" OR TS="hypersensitivit*" OR TS="atopic sensitization" OR TS="atop*" OR TS="allergic sensitization" OR TS="allergic disease*" OR TS="allerg*" OR TS="atopic dermatitis" OR TS="dermatitis" OR TS="atopic eczema" OR TS="eczema" OR TS="neurodermatiti*" OR TS="besnier’s prurigo" OR TS="besniers prurigo" OR TS="besnier prurigo" OR TS="urticari*" OR TS="anaphyla*" OR TS="quincke’s edema" OR TS="quinckes edema" OR TS="quincke edema" OR TS="angioneurotic edema" OR TS="angioedema" OR TS="hives" OR TS="food hypersensitivit*" OR TS="food allerg*" OR TS="egg allerg*" OR TS="egg hypersensitivit*" OR TS="milk allerg*" OR TS="milk hypersensitivit*" OR TS="shellfish allerg*" OR TS="shellfish hypersensitivit*" OR TS="wheat allerg*" OR TS="wheat hypersensitivit*" OR TS="nut allerg*" OR TS="nut hypersensitivit*" OR TS="peanut allerg*" OR TS="peanut hypersensitivit*" OR TS="groundnut allerg*" OR TS="groundnut hypersensitivit*" OR TS="pollen allerg*" OR TS="allergic rhinoconjunctiviti*" OR TS="rhinoconjunctiviti*" OR TS="seasonal allergic rhiniti*" OR TS="perennial allergic rhiniti*" OR TS="allergic rhiniti*" OR TS="rhiniti*" OR TS="allergic conjunctiviti*" OR TS="vernal keratoconjunctiviti*" OR TS="vernal conjunctiviti*" OR TS="giant papillary conjunctiviti*" OR TS="hay fever" OR TS="hayfever" OR TS="pollinosis" OR TS="pollenosis" OR TS="nasal catarrh*")

TS = title, abstract, author keywords, Keywords Plus
