# Supplementary material for: Sibship size, birth order and risk of asthma and allergy: protocol for a systematic review and meta-analysis
Source: BMJ Open. 2021 Aug 23;11(8):e045795. doi: 10.1136/bmjopen-2020-045795 (PMC8383851; doi:10.1136/bmjopen-2020-045795)
Supplement: Supplementary data [file bmjopen-2020-045795supp003.pdf]

Appendix to

Sibship Size, Birth Order and Risk of Asthma and Allergy: Protocol for a Systematic Review and Meta-Analysis

Daniil Lisik, Athina Ioannidou, Gregorio Paolo Milani, Sungkutu Nyassi, Saliha Selin Özuygur Ermis, Giulia Spolidoro, Emma Goksör, Göran Wennergren, Bright I Nwaru

Appendix 3: Data Extraction Form

Reviewer (initials)	
Date of data extraction (yyyy-mm-dd)	
**General information**	
Author (for first author: surname, given name(s))	
Title of article	
Year of publication	
Country of origin of study	
Contact information to author(s)	
**Study characteristics**	
Study design	
Study aims/objectives	
Exposure(s) (for each exposure: 1) method(s) of assessment; 2) objectivity of assessment (objective/subjective[1]); 3) validity (yes[2]/no); 4) reliability (yes[3]/no))	
Outcome(s) (for each outcome: 1) method(s) of assessment; 2) objectivity (objective/subjective[4]); 3) validity (yes[2]/no); 4) reliability (yes[3]/no))	
Follow-up (method; length)	Method:<br>Length:<br><br>Or: ☐ not applicable
Study was conducted during (year(s))	
**Participant selection**	
Inclusion criteria	
Exclusion criteria	
Source(s) of subjects	
**Population characteristics**	
Participants recruited (n; details)	n:<br>Details:

Lisik D, *et al. BMJ Open* 2022; 11:e045795. doi: 10.1136/bmjopen-2020-045795

Participants eligible (n; % of "Participants recruited"; eligibility criteria)	n:<br>% of "Participants recruited":<br>Eligibility criteria:
Participants included (n; % of "Participants eligible")	n:<br>% of "Participants eligible":<br><br>Or:
Participants completing follow-up (n; % of "Participants included")	n:<br>% of "Participants included":<br><br>Or:
Participants lost (n; % of "Participants included"; details; how it was dealt with)	n:<br>% of "Participants included":<br>Details:<br>How it was dealt with:<br><br>Or:
Data lost (n; % of "Participants included"; details; how it was dealt with)	n:<br>% of "Participants included":<br>Details:<br>How it was dealt with:
Participants characteristics (for each group: 1) n_{total}; 2) age (mean (SD)); 3) gender distribution (n_{males} (% of n_{total})); 4) ethnicity; 5) country; 6) economic classification of country by the World Bank; 7) setting; 8) co-morbidity)	
**Results**	
Outcomes (for each outcome for each exposure, stratified by group[5] if applicable: 1) n (% of n_{group}); 2) effect size (measure of effect) 95% CI; 3) p-value)	
**Analysis**	
Statistical analysis method	
Confounders (what confounders were identified; how they were controlled for; % of confounders controlled for)	Confounders identified:<br>How they were controlled for:<br><br>% of confounders controlled for:
Free-text interpretation of findings/conclusion	
Generalizability (is it likely that individuals selected for this study to be representative of the target population?)	
**Miscellaneous**	
Other comments/notes	

**Quality assessment (based on the Effective Public Health Practice Project (EPHPP) quality assessment tool[6])**	
**(A) Selection bias**	
(Q1) Is it likely that individuals selected for this study to be representative of the target population?	
(Q2) How many of eligible individuals agreed to participate in the study? (%)	
Section rating	
**(B) Study design**	
Indicate the design of the study	
Was the study setting randomized? If "No", go to (C)	
If "Yes", was the randomization method described?	
If "Yes", was the method referred to above appropriate?	
Section rating<br>Rate longitudinal studies as "moderate", and cross-sectional studies as "weak"	
**(C) Confounders**	
(Q1) Did the groups have significant differences in relation to each other prior to the intervention?	
(Q2) If "Yes", indicate how many relevant confounders that were controlled for in any way (e.g. in study design through matching, stratification, or in analysis) (%)	
Section rating<br>Rate studies without a control group as weak	
**(D) Blinding**	
(Q1) Did the outcome assessor(s) know about the exposure status of the participants?	
(Q2) Did the participants of the study know about the research question?	
Section rating<br>Rate as "weak" if Q1 is 1 and Q2 is 3	
**(E) Data collection methods**	
(Q1) Were the tools used for data collection shown to be valid?	
(Q2) Were the tools used for data collection shown to be reliable?	

Lisik D, *et al. BMJ Open* 2022; 11:e045795. doi: 10.1136/bmjopen-2020-045795

Section rating	
**(F) Withdrawals and drop-outs**	
(Q1) Did numbers and/or reasons for withdrawals and drop-outs per group get documented?	
(Q2) How many participants completed the study (if the value is different between groups, state the lowest)? (%)	
Section rating	
**Global rating**	
Did the two reviewers give different section ratings for A-F?	
If "Yes", what is the reason for the difference(s)?	
**Final rating of both reviewers**	

[1] Objective: medical records/official statistics. Subjective: self-report, observation.
[2] Yes: The assessment gives usable, meaningful information for the research question.
[3] Yes: Results from assessment type are consistent and stable.
[4] Objective: ICD code, verified diagnosis based on medical examination. Subjective: otherwise observed or self-reported symptoms/disease.
[5] Applicable if specific stratification has been made in the analysis, e.g. significant differences and/or calculations based on gender of participants.
[6] Modified version of EPHPP [1]. Original tool is available at: https://www.ephpp.ca/quality-assessment-tool-for-quantitative-studies/. The questions and answer alternatives are modified in phrasing for readability and the nature of relevant studies. Modifications of rating are clarified in green text above, based on the modifications of EPHPP done in a systematic review by Smith et al. in 2017 [2].

References

1. Armijo-Olivo S, Stiles CR, Hagen NA, Biondo PD, Cummings GG. Assessment of study quality for systematic reviews: a comparison of the Cochrane Collaboration Risk of Bias Tool and the Effective Public Health Practice Project Quality Assessment Tool: methodological research. J Eval Clin Pract. 2012;18(1):12-18.
2. Smith M, Hosking J, Woodward A, Witten K, MacMillan A, Field A, et al. Systematic literature review of built environment effects on physical activity and active transport – an update and new findings on health equity. International Journal of Behavioral Nutrition and Physical Activity. 2017;14(1):158.

Lisik D, *et al. BMJ Open* 2022; 11:e045795. doi: 10.1136/bmjopen-2020-045795
