# Supplementary material for: Sibship size, birth order and risk of asthma and allergy: protocol for a systematic review and meta-analysis
Source: BMJ Open. 2021 Aug 23;11(8):e045795. doi: 10.1136/bmjopen-2020-045795 (PMC8383851; doi:10.1136/bmjopen-2020-045795)
Supplement: Author's manuscript [file bmjopen-2020-045795.draft_revisions.pdf]

BMJ Open is committed to open peer review. As part of this commitment we make the peer review history of every article we publish publicly available.

When an article is published we post the peer reviewers' comments and the authors' responses online. We also post the versions of the paper that were used during peer review. These are the versions that the peer review comments apply to.

The versions of the paper that follow are the versions that were submitted during the peer review process. They are not the versions of record or the final published versions. They should not be cited or distributed as the published version of this manuscript.

BMJ Open is an open access journal and the full, final, typeset and author-corrected version of record of the manuscript is available on our site with no access controls, subscription charges or pay-per-view fees (http://bmjopen.bmj.com).

If you have any questions on BMJ Open's open peer review process please email info.bmjopen@bmj.com

BMJ Open

Sibship Size, Birth Order and Risk of Asthma and Allergy: Protocol for a Systematic Review and Meta-Analysis

Journal:	*BMJ Open*
Manuscript ID	bmjopen-2020-045795
Article Type:	Protocol
Date Submitted by the Author:	12-Oct-2020
Complete List of Authors:	Lisik, Daniil; Sahlgrenska Academy, Medicine<br>Özuygur Ermis, Saliha Selin; Dokuz Eylül University, Department of Pulmonology<br>Goksör, Emma; Univ Gothenburg<br>Wennergren, Göran; University of Gothenburg Sahlgrenska Academy, Krefting Research Centre; University of Gothenburg Sahlgrenska Academy, Dept of Paediatrics<br>Nwaru, Bright; University of Gothenburg, Krefting Research Centre
Keywords:	Asthma < THORACIC MEDICINE, Allergy < THORACIC MEDICINE, Eczema < DERMATOLOGY, IMMUNOLOGY, Paediatric infectious disease & immunisation < PAEDIATRICS, Paediatric dermatology < PAEDIATRICS

SCHOLARONE™
Manuscripts

BMJ

I, the Submitting Author has the right to grant and does grant on behalf of all authors of the Work (as defined in the below author licence), an exclusive licence and/or a non-exclusive licence for contributions from authors who are: i) UK Crown employees; ii) where BMJ has agreed a CC-BY licence shall apply, and/or iii) in accordance with the terms applicable for US Federal Government officers or employees acting as part of their official duties; on a worldwide, perpetual, irrevocable, royalty-free basis to BMJ Publishing Group Ltd ("BMJ") its licensees and where the relevant Journal is co-owned by BMJ to the co-owners of the Journal, to publish the Work in this journal and any other BMJ products and to exploit all rights, as set out in our licence.

The Submitting Author accepts and understands that any supply made under these terms is made by BMJ to the Submitting Author unless you are acting as an employee on behalf of your employer or a postgraduate student of an affiliated institution which is paying any applicable article publishing charge ("APC") for Open Access articles. Where the Submitting Author wishes to make the Work available on an Open Access basis (and intends to pay the relevant APC), the terms of reuse of such Open Access shall be governed by a Creative Commons licence – details of these licences and which Creative Commons licence will apply to this Work are set out in our licence referred to above.

Other than as permitted in any relevant BMJ Author's Self Archiving Policies, I confirm this Work has not been accepted for publication elsewhere, is not being considered for publication elsewhere and does not duplicate material already published. I confirm all authors consent to publication of this Work and authorise the granting of this licence.

For peer review only

Sibship Size, Birth Order and Risk of Asthma and Allergy: Protocol for a Systematic Review and Meta-Analysis

Daniil Lisik[1], Saliha Selin Özuygur Ermis[2], Emma Goksör[3], Göran Wennergren[3], Bright I Nwaru[4]

Author affiliations

[1] Institute of Medicine, Sahlgrenska Academy, University of Gothenburg, Sweden

[2] Department of Pulmonology, Dokuz Eylul University, Izmir, Turkey

[3] Department of Pediatrics, University of Gothenburg, Queen Silvia Children's Hospital, Gothenburg, Sweden.

[4] Krefting Research Centre, Institute of Medicine, University of Gothenburg, Sweden

Correspondence to

Daniil Lisik
Institute of Medicine
Sahlgrenska Academy
University of Gothenburg, Sweden
guslisda@student.gu.se

For peer review only

Abstract

Introduction: The hygiene hypothesis suggests that reduced exposure to microbes might have contributed to the increase in prevalence and incidence of asthma and allergy observed during the second half of the last century. Following this proposal, several studies have investigated the role of sibship size and birth order in the development of asthma and allergic diseases, but the underlying evidence is conflicting. A systematic review will provide a clearer appreciation of the strength, magnitude, and quality of the underlying evidence.

Objective: To identify, critically appraise and synthesize previous primary studies investigating the association of sibship size and birth order with the risk of asthma and allergic diseases.

For peer review only - http://bmjopen.bmj.com/site/about/guidelines.xhtml

Methods and analysis: The following databases will be searched: AMED, CABI, CINAHL, Embase, Google Scholar, OAIster, Open Access Theses and Dissertations, Open Grey, ProQuest Dissertations & Theses Global, PsycINFO, PubMed, SciELO, Scopus, Web of Science, and WHO Global Index Medicus. Studies published up until 31st December 2020 will be eligible. There will be no restrictions by language and geographical location. Risk of bias in the included studies will be assessed using the Effective Public Health Practice Project (EPHPP) quality assessment tool. The produced evidence will be synthesized narratively, and studies that present comparable numerical data will be included in meta-analyses using random-effects model.

Ethics and dissemination: Only data from the published literature will be included in this systematic review. Therefore, no ethical approval is required. The final review paper will be published in a peer-reviewed journal.

PROSPERO registration number: The protocol has been submitted in PROSPERO and awaits a registration number.

For peer review only

Strengths and limitations of this study

- This will be the first systematic review encompassing a comprehensive spectrum of the most common allergic and respiratory outcomes, in relation to sibship size and birth order.
- Inclusion of the leading databases, including search of the gray literature, enables a comprehensive identification of the relevant studies addressing the research question.
- The reproducibility of our work is enhanced through a priori outline of the review processes before the actual review starts.
- Self-reported diagnoses of the study outcomes are expected to make up a significant source of data from included studies, which gives the possibility of assessment bias.

Introduction

The incidence and prevalence of asthma, along with allergic diseases such as allergic rhinitis and atopic eczema, were observed to have increased during the second half of the last

For peer review only - http://bmjopen.bmj.com/site/about/guidelines.xhtml

century, in particular in the developed world [1, 2]. More recent trends remain unclear, as both increase [3] and leveling off [1, 4, 5] have been suggested. Around 300 million people have asthma globally [1]. For allergic diseases, evidence indicates that there is still a global increase in prevalence [5, 6]. Asthma and allergic diseases account for significant morbidity for individuals, as well as a substantial socio-economic burden on the society [2]. Asthma results in roughly 14 million missed school days each year in the United States alone, and the morbidity is even higher for adults [7]. Allergic rhinitis is also associated with significant loss in productivity [8]. Furthermore, the World Health Organization estimates that roughly 250,000 cases of death annually, worldwide, are due to asthma [7]. Identifying risk factors for asthma and allergy is therefore of great interest, in order to reduce the burden associated with these diseases.

Over the last five decades, numerous hypotheses have been proposed to explain the observed increase in the prevalence of these diseases, a substantial part of the studies focusing on the role of environmental factors. One of the main hypotheses is the hygiene hypothesis, which was first proposed by Strachan in 1989, and suggests that reduced microbial exposure during childhood increases the risk of developing asthma and allergy [9]. Connected to this hypothesis is the proposed sibling effect, which suggests that the number of siblings and/or the birth order of a child in a family may play a role in the development of asthma and allergy, as a result of varying degrees of microbial exposure during childhood, depending on the number of siblings in total and/or the number of older siblings [10].

While several studies have investigated the association of sibship (group of individuals sharing the same pair of parents) size and birth order (the sequence in which members of a sibship are born) with risk of asthma and allergic diseases, findings are conflicting [11]. So far, there are no systematic reviews synthesizing evidence from previous studies on the topic. A systematic synthesis of previous studies investigating the association of sibship size and birth order with risk of asthma and allergy will provide a clearer appreciation of the strength, magnitude, and quality of the underlying evidence.

Aim

For peer review only - http://bmjopen.bmj.com/site/about/guidelines.xhtml

For peer review only

To identify, critically appraise and synthesize previous primary studies investigating the association of sibship size and birth order with risk of asthma and allergic diseases.

Methods and analyses

This protocol is reported according to the recommendations of the Preferred Reporting Items for Systematic Reviews and Meta-Analysis Protocol (PRISMA-P) [12], which provides guidelines for a standardized, transparent and reproducible reporting of systematic review protocols. Updates to the protocol will be documented, and deviations from the protocol will be described in the final review paper. The protocol for this systematic review has been prospectively registered with the international prospective register of systematic reviews (PROSPERO, https://www.crd.york.ac.uk/PROSPERO) and the registration number is being awaited.

Study eligibility criteria

Study types and publication status

We will include observational epidemiological studies, including prospective and retrospective cohort studies, case-control studies, and cross-sectional studies. Randomized controlled studies, quasi-randomized controlled studies, controlled before-after studies, and controlled clinical trials will not be considered, as interventional studies are not relevant for this research question. Animal studies, reviews, case studies, case series, expert opinions will also be excluded. Studies of any publication status will be eligible, and data used from them if available.

Participants

Offspring of any age, gender, ethnic background and medical background, where the study context is that the participants are part of defined sibships.

Exposures

Sibship size and birth order in the studied sibships.

Outcome measures

For peer review only - http://bmjopen.bmj.com/site/about/guidelines.xhtml

Self-reported or objectively measured/diagnosed asthma and allergic disease in the sibships. For the purpose of encompassing all relevant literature on the topic, asthma and allergic disease will be defined broadly. Asthma will be defined as any type of asthma, including those based on symptom definition, such as wheezing, and those based on spirometry findings of variable expiratory airflow limitation [13]. Allergic disease will encompass any of the following: (a) allergic rhinitis/(rhino)conjunctivitis, food allergy, atopic eczema, urticaria, angioedema, anaphylaxis [14]; (b) indicators of hypersensitivity (and indirectly of allergic disease), which includes allergen-specific serum immunoglobulin E (IgE) test, skin prick test (SPT), provocation/challenge test. Conditions with primarily a genetic etiology, such as hereditary angioedema [15], will not be included in these definitions.

Search methods

The search queries were developed using the PEO model: population, exposure and outcome (PEO). PEO is a specific implementation of PICO, used as a framework to produce effective search queries from formulated research questions, especially befitting retrieval of interventional and observational studies [16]. Since the population (P) will be defined broadly, i.e., including both studies in children and adults, the actual search queries will be composed of two blocks; exposure (E) and outcome (O). A scoping search was performed in PubMed to identify previous studies on the topic and map relevant search terms. The search terms identified were: Medical Subject Headings (MeSH), and their corresponding alternatives in other databases; entry terms; free-text words and phrases. Subsequent scoping searches were made in PubMed with boolean operator "NOT" between various MeSH and free-text terms, alternately, in order to identify more synonyms and related search terms. The developed search terms have been piloted and refined before they will be used to identify relevant studies. The search queries have been modified for each database to be searched in regards to, inter alia, support for controlled vocabulary and syntax. Peer Review of Electronic Search Strategies (PRESS) has been used to identify potential weaknesses in the search strategy. Details of the search strategy are presented in Appendix 1.

Studies will be retrieved from the following databases: AMED (via Ovid), CABI, CINAHL (via EBSCO), Embase (via Ovid), Google Scholar, PsycINFO (via ProQuest), PubMed, SciELO, Scopus, Web of Science, and WHO Global Index Medicus. In addition, unpublished

For peer review only - http://bmjopen.bmj.com/site/about/guidelines.xhtml

articles and gray literature will be retrieved through searches of OAIster, Open Access Theses and Dissertations, Open Grey, and ProQuest Dissertations & Theses Global. Finally, studies will also be included from reference lists of the studies included in the review, as well as through contact with experts who have published in the field. All databases will be searched for articles published from inception of respective database up until 31st December 2020; an updated search will be performed at the completion of the review to ensure inclusion of studies published after the first search. There will be no language restrictions, and articles will be translated into English where possible. Articles that could not be translated will be reported in the final review paper. In Google Scholar, due to the fact that the amount of results is sometimes overwhelming, results will be retrieved from the first 300 hits [17]. Furthermore, the search query for Google Scholar has been significantly simplified, including only the most important terms in each block, due to an upper limit of 256 characters for search strings [18].

Data management

EndNote will be used for de-duplication, full-text retrieval, secondary screening, and for general management of retrieved studies. For primary screening, the articles will be imported to Rayyan QCRI.

Screening/selection process

The first stage of screening will be based on the title and/or abstract of the articles. Articles that are clearly not relevant to the research question or clearly meet any of the exclusion criteria will be excluded. Articles, where there is doubt about relevancy, will be included to the next step. In the second stage of screening, the full text of the articles will be retrieved and assessed for eligibility. The reason for each article not being included will be documented and presented in a PRISMA flow diagram in the final review paper [19]. The screening/selection will be independently performed by two reviewers. A third reviewer will arbitrate any disagreement.

Data extraction

A data extraction form (Appendix 2) has been developed to extract data from included studies in a standardized and reproducible fashion. The form will be piloted and revised

For peer review only - http://bmjopen.bmj.com/site/about/guidelines.xhtml

before being used in the review. If a study does not present needed data, authors of the study will be contacted. Extracted data will be presented in table form. The extraction will be independently conducted by two reviewers. A third reviewer will arbitrate any disagreement.

Data items

The following data items will be summarized from each study: author of publication; country of origin of study; publication year; type of study design; sample size of study; source from where study participants were recruited; definition and assessment of sibship and birth order; duration of follow-up; confounding factors adjusted included in studies; study outcomes and their assessment; analysis methods; and main results.

Quality assessment

Quality and risk of bias in the individual, included studies will be assessed using the Effective Public Health Practice Project Quality Assessment Tool (EPHPP) [20]. The EPHPP contains six domains of assessment for each study, including study design, selection bias, confounding, blinding, study collection, withdrawals and dropouts. Based on the grading of each of the six domains, a global quality grading will be derived for each study. Detailed results will be presented in a separate table in the final review paper. Appraisal of quality and risk of bias will be independently performed by two reviewers. A third reviewer will arbitrate any disagreement.

Data synthesis

Descriptive tables will be generated to present the key characteristics of the included studies. The produced evidence will be synthesized narratively. In addition, studies that present comparable numerical data will be synthesized quantitatively with meta-analyses in RevMan 5, to produce pooled effect size estimates. Random-effects model will be applied in the meta-analyses, because the included studies, solely based on published literature, are anticipated to not be similar in every aspect and thus do not estimate the same effect. This model is more conservative and provides a realistic scenario in the context of studies gathered solely from published literature [21]. Separate meta-analyses will be undertaken for each of the factors investigate (sibship size and birth order) in relation to each asthma and allergy outcome. The results of the meta-analyses will be presented in forest plots.

For peer review only - http://bmjopen.bmj.com/site/about/guidelines.xhtml

Risk ratio (RR) will be used as the outcome measure in the meta-analyses, because of its intuitive interpretative feature [22]. Data from studies presenting effect measures as odds ratio (OR), incidence rate ratio (IRR), or hazard ratio (HR) will be converted to estimates of RR before combining with other studies, using the following formulas:

(a) RR ≈ IRR;

(b) RR ≈ HR or OR (if outcome is < 15% by the end of follow-up);

(c) RR ≈ $\sqrt{OR}$ or $\frac{1 - 0.5^{\sqrt{HR}}}{1 - 0.5^{\sqrt{\frac{1}{HR}}}}$ (if outcome is ≥ 15% by the end of follow-up) [23]

Calculation of I^2 will quantify heterogeneity between the included studies [24]. Consideration will be taken, regarding that this statistic can be biased in meta-analyses with few studies [25]. Subgroup analysis will be performed to explore potential reasons for heterogeneity between studies with the following subgroup variables: (a) study design; (b) quality appraisal of studies; (c) classification of the study country into “high-income”, “upper-middle-income”, “lower-middle-income”, and “low-income” economy, as defined by the World Bank [26]; (d) time during which the study was conducted, grouped into <1990, 1990-1999, 2000-2009, and 2010-2020. Subgroup analysis will be performed if there will be at least 4 (arbitrarily chosen cutoff [27]) studies in at least 2 subgroups. In addition, if more than 10 included studies present comparable numerical data [28], meta-regression will be performed to explore the impact of explanatory variables (covariates) on the observed heterogeneity in estimates across studies.

To investigate whether the conclusions of the review are independent of arbitrary decisions, sensitivity-analysis will be performed by only including studies which (a) reach either “strong” or “moderate” global rating of quality in accordance to EPHPP; (b) have objectively verified diagnosis of asthma or allergic disease as outcome, with either ICD codes or verified medical examination as the basis for diagnosis. The sensitivity analysis will be reported in a summary table.

Publication bias

Publication bias will be assessed with Funnel plot, as well as Begg’s rank test and Egger’s regression test [29, 30]. In case of (significant) publication bias, the trim-and-fill method will be implemented to analyze its influence on the review results [31].

For peer review only - http://bmjopen.bmj.com/site/about/guidelines.xhtml

Ethics

Ethical approval will not be required due to the study being a systematic review of already published primary studies available in the public domain. Furthermore, patient consent will not be needed since data will be (or stay) aggregated [32, 33].

Patient and public involvement

No patients or participants were involved in the development of this protocol or the design of this study.

Discussion

The conclusions that will potentially be drawn from this systematic review, will be limited by the quality of the included studies. For this research question, the fact that all included studies will be observational limits the establishment of causality between sibship size, birth order, and risk of asthma and allergic diseases [34].

A strength of this study is the comprehensiveness of the search strategy, including 15 of the leading databases of formally published literature, as well as gray literature. There will be no restrictions in terms of language or geographical location. All these enable comprehensive identification of relevant studies for this research question. Furthermore, this systematic review will encompass a comprehensive spectrum of the most common asthma and allergic outcomes in relation to sibship size and birth order, thereby contributing to a broad overview of the existing evidence on the topic.

Asthma and allergic diseases pose a significant burden on both individuals and society. Whilst the role of sibship size and birth order in the development of these diseases have been investigated in several studies, albeit with conflicting evidence, a systematic review of existing studies is essential in providing a clearer appreciation of the underlying evidence. This protocol presents the methodology to perform a comprehensive systematic review and meta-analysis of existing literature on the topic.

Contributions

For peer review only - http://bmjopen.bmj.com/site/about/guidelines.xhtml

BIN conceived the study idea and research question. DL wrote the protocol, with review and general suggestions for improvement from SSÖE, EG, GW, and BIN. Sectional authors approved the last version being submitted.

Funding

There was no specific funding for this paper.

Competing interests

None declared.

References

1. Dharmage SC, Perret JL, Custovic A. Epidemiology of Asthma in Children and Adults. Front Pediatr. 2019;7:246.
2. Nunes C, Pereira AM, Morais-Almeida M. Asthma costs and social impact. Asthma Res Pract. 2017;3:1.
3. Lundbäck B, Backman H, Lötvall J, Rönmark E. Is asthma prevalence still increasing? Expert Rev Respir Med. 2016;10(1):39-51.
4. Martinez FD. Trends in asthma prevalence, admission rates, and asthma deaths. Respir Care. 2008;53(5):561-565; discussion 565-567.
5. Hicke-Roberts A, Åberg N, Wennergren G, Hesselmar B. Allergic rhinoconjunctivitis continued to increase in Swedish children up to 2007, but asthma and eczema levelled off from 1991. Acta Paediatr. 2017;106(1):75-80.
6. Doll RJ, Joseph NI, McGarry D, Jhaveri D, Sher T, Hostoffer R. Epidemiology of Allergic Diseases. Allergy and Asthma: The Basics to Best Practices. Cham: Springer International Publishing; 2019. p. 31-51.
7. Pawankar R. Allergic diseases and asthma: a global public health concern and a call to action. World Allergy Organ J. 2014;7(1):12.
8. Bousquet J, Khaltaev N, Cruz AA, Denburg J, Fokkens WJ, Togias A, et al. Allergic Rhinitis and its Impact on Asthma (ARIA) 2008 update (in collaboration with the World Health Organization, GA(2)LEN and AllerGen). Allergy. 2008;63 Suppl 86:8-160.
9. Strachan DP. Hay fever, hygiene, and household size. BMJ (Clinical research ed). 1989;299(6710):1259-1260.
10. Bloomfield SF, Rook GA, Scott EA, Shanahan F, Stanwell-Smith R, Turner P. Time to abandon the hygiene hypothesis: new perspectives on allergic disease, the human microbiome, infectious disease prevention and the role of targeted hygiene. Perspect Public Health. 2016;136(4):213-224.
11. Goldberg S, Israeli E, Schwartz S, Shochat T, Izbicki G, Toker-Maimon O, et al. Asthma prevalence, family size, and birth order. Chest. 2007;131(6):1747-1752.
12. Moher D, Shamseer L, Clarke M, Ghersi D, Liberati A, Petticrew M, et al. Preferred reporting items for systematic review and meta-analysis protocols (PRISMA-P) 2015 statement. Systematic Reviews. 2015;4(1):1.
13. Zeller TA. Respiratory Conditions Update: Asthma. FP Essent. 2016;448:11-19.
14. Pinart M, Albang R, Maier D, Duran-Tauleria E, Mena G, Gimeno-Santos E, et al. Systematic Review on the Definition of Allergic Diseases in Children: The MeDALL Study. International Archives of Allergy and Immunology. 2015;168(2):110-121.
15. Banerji A. Hereditary angioedema: classification, pathogenesis, and diagnosis. Allergy Asthma Proc. 2011;32(6):403-407.
16. Khan KS, Kunz R, Kleijnen J, Antes G. Five steps to conducting a systematic review. J R Soc Med. 2003;96(3):118-121.

For peer review only - http://bmjopen.bmj.com/site/about/guidelines.xhtml

17. Haddaway NR, Collins AM, Coughlin D, Kirk S. The Role of Google Scholar in Evidence Reviews and Its Applicability to Grey Literature Searching. PLOS ONE. 2015;10(9):e0138237.
18. Gusenbauer M, Haddaway NR. Which academic search systems are suitable for systematic reviews or meta-analyses? Evaluating retrieval qualities of Google Scholar, PubMed, and 26 other resources. Research Synthesis Methods. 2020;11(2):181-217.
19. Liberati A, Altman DG, Tetzlaff J, Mulrow C, Gøtzsche PC, Ioannidis JP, et al. The PRISMA statement for reporting systematic reviews and meta-analyses of studies that evaluate healthcare interventions: explanation and elaboration. Bmj. 2009;339:b2700.
20. Armijo-Olivo S, Stiles CR, Hagen NA, Biondo PD, Cummings GG. Assessment of study quality for systematic reviews: a comparison of the Cochrane Collaboration Risk of Bias Tool and the Effective Public Health Practice Project Quality Assessment Tool: methodological research. J Eval Clin Pract. 2012;18(1):12-18.
21. Borenstein M, Hedges LV, Higgins JP, Rothstein HR. A basic introduction to fixed-effect and random-effects models for meta-analysis. Res Synth Methods. 2010;1(2):97-111.
22. Davies HT, Crombie IK, Tavakoli M. When can odds ratios mislead? Bmj. 1998;316(7136):989-991.
23. VanderWeele TJ, Ding P. Sensitivity Analysis in Observational Research: Introducing the E-Value. Ann Intern Med. 2017;167(4):268-274.
24. Higgins JP, Thompson SG, Deeks JJ, Altman DG. Measuring inconsistency in meta-analyses. Bmj. 2003;327(7414):557-560.
25. von Hippel PT. The heterogeneity statistic I(2) can be biased in small meta-analyses. BMC Med Res Methodol. 2015;15:35.
26. World Bank. World Bank country and lending groups. Washington (DC): World Bank; 2020 [cited 2020 Sept 6]. Available from: https://datahelpdesk.worldbank.org/knowledgebase/articles/906519.
27. Fu R, Gartlehner G, Grant M, Shamliyan T, Sedrakyan A, Wilt TJ, et al. AHRQ Methods for Effective Health Care Conducting Quantitative Synthesis When Comparing Medical Interventions: AHRQ and the Effective Health Care Program. Methods Guide for Effectiveness and Comparative Effectiveness Reviews. Rockville (MD): Agency for Healthcare Research and Quality (US); 2008.
28. Schmid CH, Stark PC, Berlin JA, Landais P, Lau J. Meta-regression detected associations between heterogeneous treatment effects and study-level, but not patient-level, factors. Journal of Clinical Epidemiology. 2004;57(7):683-697.
29. Egger M, Davey Smith G, Schneider M, Minder C. Bias in meta-analysis detected by a simple, graphical test. Bmj. 1997;315(7109):629-634.
30. Lin L, Chu H. Quantifying publication bias in meta-analysis. Biometrics. 2018;74(3):785-794.
31. Shi L, Lin L. The trim-and-fill method for publication bias: practical guidelines and recommendations based on a large database of meta-analyses. Medicine (Baltimore). 2019;98(23):e15987.
32. Wormald R, Evans J. What Makes Systematic Reviews Systematic and Why are They the Highest Level of Evidence? Ophthalmic Epidemiology. 2018;25(1):27-30.
33. Suri H. Ethical Considerations of Conducting Systematic Reviews in Educational Research. In: Zawacki-Richter O, Kerres M, Bedenlier S, Bond M, Buntins K, editors. Systematic Reviews in Educational Research: Methodology, Perspectives and Application. Wiesbaden: Springer Fachmedien Wiesbaden; 2020. p. 41-54.
34. Petrisor B, Bhandari M. The hierarchy of evidence: Levels and grades of recommendation. Indian J Orthop. 2007;41(1):11-15.

For peer review only

For peer review only - http://bmjopen.bmj.com/site/about/guidelines.xhtml

Appendix to

Sibship Size, Birth Order and Risk of Asthma and Allergy: Protocol for a Systematic Review and Meta-Analysis

Daniil Lisik, Saliha Selin Özuygur Ermis, Emma Goksör, Göran Wennergren, Bright I Nwaru

Appendix 1: Search Strategies

Colorization

Red: controlled vocabulary/thesaurus

Blue: free-text

Green: referral to search query component (table row; #)

AMED

#	Search term(s)
1	(birth order* or birth rank* or multiple birth* or parity).mp.
2	exp Family Characteristics/ or (family characteristic* or family size* or family structure* or family demograph* or family composition or household size* or household demograph* or household composition).mp.
3	exp Sibling Relations/ or (sibling* or sister* or brother* or sibship size* or sibship*).mp.
4	or/1-3
5	exp Asthma/ or (bronchial asthma* or exercise-induced asthma* or exercise-induced bronchospasm* or asthma* or respiratory hypersensitivit* or airway hyper responsiveness or airway hyper-responsiveness or respiratory hyper responsiveness or respiratory hyper-responsiveness or wheez*).mp.
6	exp Hypersensitivity/ or exp Hypersensitivity Immediate/ or exp Hypersensitivity Delayed/ or (immediate hypersensitivit* or delayed hypersensitivit* or IgE-mediated hypersensitivit* or type I hypersensitivit* or type IV hypersensitivit* or atopic sensitization or atop* or allergic sensitization or allerg*).mp.
7	exp Dermatitis/ or exp Anaphylaxis/ or (atopic dermatitis or dermatitis or neurodermatiti* or besniers prurigo or besnier prurigo or atopic eczema or eczema or urticari* or anaphyla* or quinckes edema or quincke edema or angioneurotic edema or angioedema or hives).mp.
8	exp Food Hypersensitivity/ or (food hypersensitivit* or food allerg* or egg hypersensitivit* or egg allerg* or milk hypersensitivit* or milk allerg* or shellfish hypersensitivit* or shellfish allerg* or wheat hypersensitivit* or wheat allerg* or nut hypersensitivit* or nut allerg* or peanut hypersensitivit* or peanut allerg* or groundnut hypersensitivit* or groundnut allerg*).mp.

For peer review only

9	exp Rhinitis/ or exp Conjunctivitis/ or (allergic rhinoconjunctiviti* or rhinoconjunctiviti* or allergic rhiniti* or seasonal allergic rhiniti* or perennial allergic rhiniti* or rhiniti* or allergic conjunctiviti* or vernal keratoconjunctiviti* or vernal conjunctiviti* or giant papillary conjunctiviti* or hay fever or hayfever or pollinosis or nasal catarrh*).mp.
10	or/5-9
11	4 and 10
Full query<br>((birth order* or birth rank* or multiple birth* or parity).mp. or exp Family Characteristics/ or (family characteristic* or family size* or family structure* or family demograph* or family composition or household size* or household demograph* or household composition).mp. or exp Sibling Relations/ or (sibling* or sister* or brother* or sibship size* or sibship*).mp.) and (exp Asthma/ or (bronchial asthma* or exercise-induced asthma* or exercise-induced bronchospasm* or asthma* or respiratory hypersensitivit* or airway hyper responsiveness or airway hyper-responsiveness or respiratory hyper responsiveness or respiratory hyper-responsiveness or wheez*).mp. or exp Hypersensitivity/ or exp Hypersensitivity Immediate/ or exp Hypersensitivity Delayed/ or (immediate hypersensitivit* or delayed hypersensitivit* or IgE-mediated hypersensitivit* or type I hypersensitivit* or type IV hypersensitivit* or atopic sensitization or atop* or allergic sensitization or allerg*).mp. or exp Dermatitis/ or exp Anaphylaxis/ or (atopic dermatitis or dermatitis or neurodermatiti* or besniers prurigo or besnier prurigo or atopic eczema or eczema or urticari* or anaphyla* or quinckes edema or quincke edema or angioneurotic edema or angioedema or hives).mp. or exp Food Hypersensitivity/ or (food hypersensitivit* or food allerg* or egg hypersensitivit* or egg allerg* or milk hypersensitivit* or milk allerg* or shellfish hypersensitivit* or shellfish allerg* or wheat hypersensitivit* or wheat allerg* or nut hypersensitivit* or nut allerg* or peanut hypersensitivit* or peanut allerg* or groundnut hypersensitivit* or groundnut allerg*).mp. or exp Rhinitis/ or exp Conjunctivitis/ or (allergic rhinoconjunctiviti* or rhinoconjunctiviti* or allergic rhiniti* or seasonal allergic rhiniti* or perennial allergic rhiniti* or rhiniti* or allergic conjunctiviti* or vernal keratoconjunctiviti* or vernal conjunctiviti* or giant papillary conjunctiviti* or hay fever or hayfever or pollinosis or nasal catarrh*).mp.)	

exp = include all narrower subject headings; mp= abstract, heading words, title

CABI; OAIster; Open Access Theses and Dissertations; Open Grey; ProQuest Dissertations & Theses Global; SciELO; WHO Global Index Medicus

#	**Search term(s)**
1	"birth order" OR "multiple births" OR "birth rank" OR "parity"
2	"family characteristics" OR "family size" OR "family structure" OR "family demography" OR "family composition" OR "household size" OR "household demography" OR "household composition"
3	"siblings" OR "sibling relations" OR "sister" OR "brother" OR "sibship"
4	"exercise-induced bronchospasm" OR "asthma" OR "airway hyper-responsiveness" OR "respiratory hyper-responsiveness" OR "wheeze" OR "wheezing"
5	”hypersensitivity" OR "atopic sensitization" OR "atopy" OR "allergic sensitization" OR "allergic disease" OR "allergic condition" OR "allergy" OR "allergies"

For peer review only

6	"dermatitis" OR "eczema" OR "neurodermatitis" OR "besnier's prurigo" OR "urticaria" OR "anaphylaxis" OR "anaphylactic shock" OR "quincke's edema" OR "angionuerotic edema" OR "angioedema" OR "hives"
8	"rhinoconjunctivitis" OR "rhinitis" OR "allergic conjunctivitis" OR "vernal keratoconjunctivitis" OR "vernal conjunctivitis" OR "giant papillary conjunctivitis" OR "hay fever" OR "pollinosis" OR "pollenosis" OR "nasal catarrh"
9	1 OR 2 OR 3
10	4 OR 5 OR 6 OR 7 OR 8
11	9 AND 10
Full query	
("birth order" OR "multiple births" OR "birth rank" OR "parity" OR "family characteristics" OR "family size" OR "family structure" OR "family demography" OR "family composition" OR "household size" OR "household demography" OR "household composition" OR "siblings" OR "sibling relations" OR "sister" OR "brother" OR "sibship") AND ("exercise-induced bronchospasm" OR "asthma" OR "airway hyper-responsiveness" OR "respiratory hyper-responsiveness" OR "wheeze" OR "wheezing" OR "hypersensitivity" OR "atopic sensitization" OR "atopy" OR "allergic sensitization" OR "allergic disease" OR "allergic condition" OR "allergy" OR "allergies" OR "dermatitis" OR "eczema" OR "neurodermatitis" OR "besnier's prurigo" OR "urticaria" OR "anaphylaxis" OR "anaphylactic shock" OR "quincke's edema" OR "angionuerotic edema" OR "angioedema" OR "hives" OR "rhinoconjunctivitis" OR "rhinitis" OR "allergic conjunctivitis" OR "vernal keratoconjunctivitis" OR "vernal conjunctivitis" OR "giant papillary conjunctivitis" OR "hay fever" OR "pollinosis" OR "pollenosis" OR "nasal catarrh")	

CINAHL

#	Search term(s)
1	(MH 'Birth Order+') OR (MH 'Parity+') OR 'birth order*' OR 'birth rank*' OR 'multiple birth*' OR 'parity'
2	(MH 'Family Characteristics+') OR (MH 'Family Health+') OR 'family charactersitic*' OR 'family size*' OR 'family structure*' OR 'family demograph*' OR 'family composition' OR 'household size*' OR 'household demograph*' OR 'household composition'
3	(MH 'Siblings+') OR (MH 'Sibling Relations+') OR 'sibling*' OR 'sister*' OR 'brother*' OR 'sibship size*' OR 'sibship*'
4	(MH 'Asthma+') OR (MH 'Respiratory Hypersensitivity+') OR 'bronchial asthma*' OR 'exercise-induced asthma' OR 'asthma*' OR 'exercise-induced bronchospasm' OR 'respiratory hypersensitivit*' OR 'airway hyper responsiveness' OR 'airway hyper-responsiveness' OR 'respiratory hyper responsiveness' OR 'respiratory hyper-responsiveness' OR 'wheez*'
5	(MH 'Hypersensitivity+') OR (MH 'Hypersensitivity, Immediate+') OR (MH 'Hypersensitivity, Delayed+') OR (MH 'Allergy and Immunology+') OR 'immedate hypersensitivit*' OR 'delayed hypersensitivit*' OR 'IgE-mediated hypersensitivit*' OR 'type I hypersensitivit*' OR 'type IV hypersensitivit*' OR 'hypersensitivit*' OR 'atopic sensitiziation' OR 'atop*' OR 'allergic sensitization' OR 'allergic disease* OR 'allerg*'

6	(MH ’Dermatitis, Atopic+’) OR (MH ’Eczema+’) OR (MH ‘Angioedema+’) OR (MH ‘Anaphylaxis+’) OR (MH ’Urticaria+’) OR ’atopic dermatitis’ OR ’dermatitis’ OR ’atopic eczema’ OR ’eczema’ OR ’nerudoarmatiti*’ OR “besnier’s prurigo” OR ‘besniers prurigo’ OR ‘besnier prurigo’ OR ’urticari*’ OR ’hives’ OR ’anaphyla*’ OR ”quincke’s edema” OR ’quinckes edema’ OR ’quincke edema’ OR ’angioneurotic edema’ OR ’angioedema’
7	(MH ’Food Hypersensitivity+’) OR ’food hypersensitivit*’ OR ’food allerg*’ OR ’egg hypersensitivit*’ OR ’egg allerg*’ OR ’milk hypersensitivit*’ OR ’milk allerg*’ OR ’shellfish hypersensitivit*’ OR ’shellfish allerg*’ OR ’wheat hypersensitivit*’ OR ’wheat allerg*’ OR ’nut hypersensitivit*’ OR ’nut allerg*’ OR ’peanut hypersensitivit*’ OR ’peanut allerg*’ OR ’groundnut hypersensitivit*’ OR ’groundnut allerg*’
8	(MH ’Rhinitis, Allergic+’) OR (MH ’Rhinitis, Allergic, Seasonal+’) OR (MH ’Rhinitis, Allergic, Perennial+’) OR (MH ’Rhinitis+’) OR (MH ’Conjunctivitis, Allergic+’) OR (MH ’Conjunctivitis+’) OR ’allergic rhinoconjunctiviti*’ OR ’rhinoconjunctiviti*’ OR ’allergic rhiniti*’ OR ’seasonal allergic rhiniti*’ OR ’perennial allergic rhiniti*’ OR ’rhiniti*’ OR ’allergic conjunctiviti*’ OR ’vernal keratoconjunctiviti*’ OR ’vernal conjunctiviti*’ OR ’giant papillary conjunctiviti*’ OR ’hay fever’ OR ’hayfever’ OR ’pollinosis’ OR ’pollenosis’ OR ’nasal catarrh’
9	1 OR 2 OR 3
10	4 OR 5 OR 6 OR 7 OR 8
11	9 AND 10

Full query

((MH "Birth Order+") OR (MH "Parity+") OR "birth order*" OR "birth rank*" OR "multiple birth*" OR "parity" OR (MH "Family Characteristics+") OR (MH "Family Health+") OR "family charactersitic*" OR "family size*" OR "family structure*" OR "family demograph*" OR "family composition" OR "household size*" OR "household demograph*" OR "household composition" OR (MH "Siblings+") OR (MH "Sibling Relations+") OR "sibling*" OR "sister*" OR "brother*" OR "sibship size*" OR "sibship*") AND ((MH "Asthma+") OR (MH "Respiratory Hypersensitivity+") OR "bronchial asthma*" OR "exercise-induced asthma" OR "asthma*" OR "exercise-induced bronchospasm" OR "respiratory hypersensitivit*" OR ‘airway hyper responsiveness" OR "airway hyper-responsiveness" OR "respiratory hyper responsiveness" OR "respiratory hyper-responsiveness" OR "wheez*" OR (MH "Hypersensitivity+") OR (MH "Hypersensitivity, Immediate+") OR (MH "Hypersensitivity, Delayed+") OR (MH "Allergy and Immunology+") OR "immedate hypersensitivit*" OR "delayed hypersensitivit*" OR "IgE-mediated hypersensitivit*" OR "type I hypersensitivit*" OR "type IV hypersensitivit*" OR "hypersensitivit*" OR "atopic sensitiziation" OR "atop*" OR "allergic sensitization" OR "allergic disease* OR "allerg*" OR (MH "Dermatitis, Atopic+") OR (MH "Eczema+") OR (MH ‘Angioedema+") OR (MH ‘Anaphylaxis+") OR (MH "Urticaria+") OR "atopic dermatitis" OR "dermatitis" OR "atopic eczema" OR "eczema" OR "nerudoarmatiti*" OR "besnier's prurigo" OR "besniers prurigo" OR "besnier prurigo" OR "urticari*" OR "hives" OR "anaphyla*" OR "quincke's edema" OR "quinckes edema" OR "quincke edema" OR "angioneurotic edema" OR "angioedema" OR (MH "Food Hypersensitivity+") OR "food hypersensitivit*" OR "food allerg*" OR "egg hypersensitivit*" OR "egg allerg*" OR "milk hypersensitivit*" OR "milk allerg*" OR "shellfish hypersensitivit*" OR "shellfish allerg*" OR "wheat hypersensitivit*" OR "wheat allerg*" OR "nut hypersensitivit*" OR "nut allerg*" OR "peanut hypersensitivit*" OR "peanut allerg*" OR "groundnut hypersensitivit*" OR "groundnut allerg*" OR (MH "Rhinitis, Allergic+") OR (MH "Rhinitis, Allergic, Seasonal+") OR (MH "Rhinitis, Allergic, Perennial+") OR (MH "Rhinitis+") OR (MH "Conjunctivitis, Allergic+") OR (MH "Conjunctivitis+") OR "allergic rhinoconjunctiviti*" OR "rhinoconjunctiviti*" OR

For peer review only

"allergic rhiniti*" OR "seasonal allergic rhiniti*" OR "perennial allergic rhiniti*" OR "rhiniti*" OR "allergic conjunctiviti*" OR "vernal keratoconjunctiviti*" OR "vernal conjunctiviti*" OR "giant papillary conjunctiviti*" OR "hay fever" OR "hayfever" OR "pollinosis" OR "pollenosis" OR "nasal catarrh"))

MH = subject heading

Embase

#	**Search term(s)**
1	sibship.mp.
2	birth order.mp. or exp birth order/
3	birth rank.mp.
4	multiple birth.mp.
5	parity.mp. or exp parity/
6	family characteristic.mp. or exp family size/
7	family structure.mp.
8	family demograph.mp.
9	family demograph*.mp.
10	family composition.mp.
11	household size.mp.
12	household demograph*.mp.
13	household composition.mp.
14	exp sibling relation/ or sibling.mp. or sibling/
15	exp sister/ or sister.mp.
16	siblings.mp.
17	sisters.mp.
18	brother.mp. or exp brother/
19	brothers.mp.
20	or/1-19
21	exp Asthma/ or (bronchial asthma* or exercise-induced asthma* or exercise-induced bronchospasm* or asthma* or respiratory hypersensitivit* or airway hyper responsiveness or airway hyper-responsiveness or respiratory hyper responsiveness or respiratory hyper-responsiveness or wheez*).mp.
22	exp Hypersensitivity/ or exp Hypersensitivity Immediate/ or exp Hypersensitivity Delayed/ or (immediate hypersensitivit* or delayed hypersensitivit* or IgE-mediated hypersensitivit* or type I hypersensitivit* or type IV hypersensitivit* or atopic sensitization or atop* or allergic sensitization or allerg*).mp.
23	exp Dermatitis/ or exp Anaphylaxis/ or (atopic dermatitis or dermatitis or neurodermatiti* or besnier prurigo or besniers prurigo or besnier prurigo or atopic eczema or eczema or urticari* or anaphyla* or quincke edema or quinckes edema or quincke edema or angioneurotic edema or angioedema or hives).mp.

24	exp Food Hypersensitivity/ or (food hypersensitivit* or food allerg* or egg hypersensitivit* or egg allerg* or milk hypersensitivit* or milk allerg* or shellfish hypersensitivit* or shellfish allerg* or wheat hypersensitivit* or wheat allerg* or nut hypersensitivit* or nut allerg* or peanut hypersensitivit* or peanut allerg* or groundnut hypersensitivit* or groundnut allerg*).mp.
25	exp Rhinitis/ or exp Conjunctivitis/ or (allergic rhinoconjunctiviti* or rhinoconjunctiviti* or allergi rhiniti* or seasonal allergic rhiniti* or perennial allergic rhiniti* or rhiniti* or allergic conjunctiviti* or vernal keratoconjunctiviti* or vernal conjunctiviti* or giant papillary conjunctiviti* or hay fever or hayfever or pollinosis or pollinosis or nasal catarrh*).mp.
26	or/21-25
27	20 and 26

Google Scholar

#	**Search term(s)**
1	"family size" OR "family structure" OR "household size"
2	"sibling" OR "sibship"
3	"asthma" OR "wheezing"
4	"atopy" OR "allergy"
5	"eczema" OR "urticaria" OR "angioedema" OR "anaphylaxis"
6	"rhinitis" OR "allergic conjunctivitis" OR "hay fever"
7	1 OR 2
8	3 OR 4 OR 5 OR 6
9	7 AND 8
Full query ("family size" OR "family structure" OR "household size" OR "sibling" OR "sibship") AND ("asthma" OR "wheezing" OR "atopy" OR "allergy" OR "anaphylaxis" OR "eczema" OR "urticaria" OR "angioedema" OR "rhinitis" OR "allergic conjunctivitis" OR "hay fever")	

PsycINFO

#	**Search term(s)**
1	SU.EXACT.EXPLODE("Birth Order") OR TI,AB(”birth order*”) OR TI,AB(”multiple birth*”) OR TI,AB(”birth rank*”) OR TI,AB(”parity”)
2	SU.EXACT.EXPLODE("Family Structure") OR SU.EXACT.EXPLODE("Family Size") OR TI,AB(”family characteristic*”) OR TI,AB(”family size*”) OR TI,AB(”family structure*”) OR TI,AB(”family demograph*”) OR TI,AB(”family composition”) OR TI,AB(”household size*”) OR TI,AB(”household demograph*”) OR TI,AB(”household composition”)
3	SU.EXACT.EXPLODE("Siblings") OR SU.EXACT.EXPLODE("Sibling Relations") OR TI,AB(”sibling*”’) OR TI,AB(”sister*”) OR TI,AB(”brother*”) OR TI,AB(”sibship size*”) OR TI,AB(”sibship size*”) OR TI,AB(”sibship*”)

For peer review only

4	SU.EXACT.EXPLODE("Asthma") OR TI,AB(”bronchial asthma*”) OR TI,AB(”exercise-induced asthma*”) OR TI,AB(”asthma*”) OR TI,AB(”exercise-induced bronchospasm*”) OR TI,AB(”respiratory hypersensitivit*”) OR TI,AB(”airway hyper responsiveness”) OR TI,AB(”airway hyper-responsiveness”) OR TI,AB(”respiratory hyper responsiveness”) OR TI,AB(”respiratory hyper-responsiveness”) OR TI,AB(”wheez*”)
5	SU.EXACT.EXPLODE("Allergic Disorders") OR TI,AB(”immediate hypersensitivit*”) OR TI,AB(”delayed hypersensitivit*”) OR TI,AB(”hypersensitivit*”) OR TI,AB(”IgE-mediated hypersensitivit*”) OR TI,AB(”type I hypersensitivit*”) OR TI,AB(”type IV hypersensitivit*”) OR TI,AB(”atopic sensitization”) OR TI,AB(”atop*”) OR TI,AB(”allergic sensitization”) OR TI,AB(”allergic disease*”) OR TI,AB(”allerg*”)
6	SU.EXACT.EXPLODE("Allergic Skin Disorders") OR SU.EXACT.EXPLODE("Neurodermatitis") OR SU.EXACT.EXPLODE("Dermatitis") OR SU.EXACT.EXPLODE("Eczema") OR SU.EXACT.EXPLODE("Anaphylactic Shock") OR TI,AB(”atopic dermatitis”) OR TI,AB(”dermatitis”) OR TI,AB(”atopic eczema”) OR TI,AB(”eczema”) OR TI,AB(”neurodermatiti*”) OR TI,AB(”besnier’s prurigo”) OR TI,AB(”besniers prurigo”) OR TI,AB(”besnier prurigo”) OR TI,AB(”urticari*”) OR TI,AB(”hives”) OR TI,AB(”anaphyla*”) OR TI,AB(”quincke’s edema”) OR TI,AB(”quinckes edema”) OR TI,AB(”quincke edema”) OR TI,AB(”angioneurotic edema”) OR TI,AB(”angioedema”)
7	SU.EXACT.EXPLODE("Food Allergies") OR TI,AB(”food hypersensitivit*”) OR TI,AB(”food allerg*”) OR TI,AB(”egg hypersensitivit*”) OR TI,AB(”egg allerg*”) OR TI,AB(”milk hypersensitivit*”) OR TI,AB(”milk allerg*”) OR TI,AB(”shellfish hypersensitivit*”) OR TI,AB(”shellfish allerg*”) OR TI,AB(”wheat hypersensitivit*”) OR TI,AB(”wheat allerg*”) OR TI,AB(”nut hypersensitivit*”) OR TI,AB(”nut allerg*”) OR TI,AB(”peanut hypersensitivit*”) OR TI,AB(”peanut allerg*”) OR TI,AB(”groundnut hypersensitivit*”) OR TI,AB(”groundnut allerg*”)
8	TI,AB(”allergic rhinoconjunctiviti*”) OR TI,AB(”rhinoconjunctiviti*”) OR TI,AB(”allergic rhiniti*”) OR TI,AB(”rhiniti*”) OR TI,AB(”seasonal allergic rhiniti*”) OR TI,AB(”perennial allergic rhiniti*”) OR TI,AB(”allergic conjunctiviti*”) OR TI,AB(”vernal keratoconjunctiviti*”) OR TI,AB(”vernal conjunctiviti*”) OR TI,AB(”giant papillary conjunctiviti*”) OR TI,AB(”hay fever”) OR TI,AB(”hayfever”) OR TI,AB(“pollinosis”) OR TI,AB(”pollenosis”) OR TI,AB(”nasal catarrh*”)
9	1 OR 2 OR 3
10	4 OR 5 OR 6 OR 7 OR 8
11	9 AND 10
Full query<br>(SU.EXACT.EXPLODE("Birth Order") OR TI,AB("birth order*") OR TI,AB("multiple birth*") OR TI,AB("birth rank*") OR TI,AB("parity") OR SU.EXACT.EXPLODE("Family Structure") OR SU.EXACT.EXPLODE("Family Size") OR TI,AB("family characteristic*") OR TI,AB("family size*") OR TI,AB("family structure*") OR TI,AB("family demograph*") OR TI,AB("family composition") OR TI,AB("household size*") OR TI,AB("household demograph*") OR TI,AB("household composition") OR SU.EXACT.EXPLODE("Siblings") OR SU.EXACT.EXPLODE("Sibling Relations") OR TI,AB("sibling*") OR TI,AB("sister*") OR TI,AB("brother*") OR	

TI,AB("sibship size*") OR TI,AB("sibship size*") OR TI,AB("sibship*")) AND (SU.EXACT.EXPLODE("Asthma") OR TI,AB("bronchial asthma*") OR TI,AB("exercise-induced asthma*") OR TI,AB("asthma*") OR TI,AB("exercise-induced bronchospasm*") OR TI,AB("respiratory hypersensitivit*") OR TI,AB("airway hyper responsiveness") OR TI,AB("airway hyper-responsiveness") OR TI,AB("respiratory hyper responsiveness") OR TI,AB("respiratory hyper-responsiveness") OR TI,AB("wheez*") OR SU.EXACT.EXPLODE("Allergic Disorders") OR TI,AB("immediate hypersensitivit*") OR TI,AB("delayed hypersensitivit*") OR TI,AB("hypersensitivit*") OR TI,AB("IgE-mediated hypersensitivit*") OR TI,AB("type I hypersensitivit*") OR TI,AB("type IV hypersensitivit") OR TI,AB("atopic sensitization") OR TI,AB("atop*") OR TI,AB("allergic sensitization") OR TI,AB("allergic disease*") OR TI,AB("allerg*") OR SU.EXACT.EXPLODE("Allergic Skin Disorders") OR SU.EXACT.EXPLODE("Neurodermatitis") OR SU.EXACT.EXPLODE("Dermatitis") OR SU.EXACT.EXPLODE("Eczema") OR SU.EXACT.EXPLODE("Anaphylactic Shock") OR TI,AB("atopic dermatitis") OR TI,AB("dermatitis") OR TI,AB("atopic eczema") OR TI,AB("eczema") OR TI,AB("neurodermatiti*") OR TI,AB("besnier's prurigo") OR TI,AB("besniers prurigo") OR TI,AB("besnier prurigo") OR TI,AB("urticari*") OR TI,AB("hives") OR TI,AB("anaphyla*") OR TI,AB("quincke's edema") OR TI,AB("quinckes edema") OR TI,AB("quincke edema") OR TI,AB("angioneurotic edema") OR TI,AB("angioedema") OR SU.EXACT.EXPLODE("Food Allergies") OR TI,AB("food hypersensitivit*") OR TI,AB("food allerg*") OR TI,AB("egg hypersensitivit*") OR TI,AB("egg allerg*") OR TI,AB("milk hypersensitivit*") OR TI,AB("milk allerg*") OR TI,AB("shellfish hypersensitivit*") OR TI,AB("shellfish allerg*") OR TI,AB("wheat hypersensitivit*") OR TI,AB("wheat allerg*") OR TI,AB("nut hypersensitivit*") OR TI,AB("nut allerg*") OR TI,AB("peanut hypersensitivit*") OR TI,AB("peanut allerg*") OR TI,AB("groundnut hypersensitivit*") OR TI,AB("groundnut allerg*") OR TI,AB("allergic rhinoconjunctiviti*") OR TI,AB("rhinoconjunctiviti*") OR TI,AB("allergic rhiniti*") OR TI,AB("rhiniti*") OR TI,AB("seasonal allergic rhiniti*") OR TI,AB("perennial allergic rhiniti*") OR TI,AB("allergic conjunctiviti*") OR TI,AB("vernal keratoconjunctiviti*") OR TI,AB("vernal conjunctiviti*") OR TI,AB("giant papillary conjunctiviti*") OR TI,AB("hay fever") OR TI,AB("hayfever") OR TI,AB("pollinosis") OR TI,AB("pollenosis") OR TI,AB("nasal catarrh*"))

SU = all subjects and indexing; TI,AB = title, abstract

For peer review only

PubMed

#	Search term(s)
1	Birth Order[mh] OR Parity[mh] OR birth order*[tiab] OR multiple birth*[tiab] OR birth rank*[tiab] OR parity[tiab]
2	Family Characteristics[mh] OR Family Health[mh] OR family characteristic*[tiab] OR family size*[tiab] OR family structure*[tiab] OR family demograph*[tiab] OR family composition[tiab] OR household size*[tiab] OR household demograph*[tiab] OR household composition[tiab]
3	Siblings[mh] OR Sibling Relations[mh] OR sibling*[tiab] OR sister*[tiab] OR brother*[tiab] OR sibship size*[tiab] OR sibship*[tiab]
4	Asthma[mh] OR Asthma, Exercise-Induced[mh] OR Respiratory Hypersensitivity[mh] OR bronchial asthma*[tiab] OR exercise-induced asthma*[tiab] OR exercise-induced bronchospasm*[tiab] OR asthma*[tiab] OR respiratory hypersensitivit*[tiab] OR airway hyper responsiveness[tiab] OR airway hyper-responsiveness[tiab] OR respiratory hyper responsiveness[tiab] OR respiratory hyper-responsiveness[tiab] OR wheez*[tiab]

5	Hypersensitivity[mh] OR Hypersensitivity, Immediate[mh] OR Hypersensitivity, Delayed[mh] OR Allergy and Immunology[mh] OR Allergens / Immunology[mh] OR immediate hypersensitivit*[tiab] OR delayed hypersensitivit*[tiab] OR hypersensitivit*[tiab] OR IgE-mediated hypersensitivit*[tiab] OR type I hypersensitivit*[tiab] OR type IV hypersensitivit*[tiab] OR atopic sensitization[tiab] OR atop*[tiab] OR allergic sensitization[tiab] OR allergic disease*[tiab] OR allerg*[tiab]
6	Dermatitis, Atopic[mh] OR Eczema[mh] OR Angioedema[mh] OR Anaphylaxis[mh] OR Urticaria[mh] OR atopic dermatitis[tiab] OR dermatitis[tiab] OR atopic eczema[tiab] OR eczema[tiab] OR neurodermatiti*[tiab] OR besnier's prurigo[tiab] OR besniers prurigo[tiab] OR besnier prurigo[tiab] OR urticari*[tiab] OR anaphyla*[tiab] OR quincke edema[tiab] OR quinckes edema[tiab] OR quincke's edema[tiab] OR angioneurotic edema[tiab] OR angioedema[tiab] OR hives[tiab]
7	Food Hypersensitivity[mh] OR food hypersensitivit*[tiab] OR food allerg*[tiab] OR egg allerg*[tiab] OR egg hypersensitivit*[tiab] OR milk allerg*[tiab] OR milk hypersensitivit*[tiab] OR shellfish allerg*[tiab] OR shellfish hypersensitivit*[tiab] OR wheat allerg*[tiab] OR wheat hypersensitivit*[tiab] OR nut allerg*[tiab] OR nut hypersensitivit*[tiab] OR peanut allerg*[tiab] OR peanut hypersensitivit*[tiab] OR groundnut hypersensitivit*[tiab]
8	Pollen / Immunology[mh] OR Rhinitis, Allergic[mh] OR Rhinitis, Allergic, Seasonal[mh] OR Rhinitis, Allergic, Perennial[mh] OR Rhinitis[mh] OR Conjunctivitis, Allergic[mh] OR Conjunctivitis / Immunology[mh] OR Conjunctivitis / Epidemiology[mh] OR Conjunctivitis / Etiology[mh] OR allergic rhinoconjunctiviti*[tiab] OR rhinoconjunctiviti*[tiab] OR allergic rhiniti*[tiab] OR rhiniti*[tiab] OR seasonal allergic rhiniti*[tiab] OR perennial allergic rhiniti*[tiab] OR allergic conjunctiviti*[tiab] OR vernal keratoconjunctiviti*[tiab] OR vernal conjunctiviti*[tiab] OR giant papillary conjunctiviti*[tiab] OR hay fever[tiab] OR hayfever[tiab] OR pollinosis[tiab] OR pollenosis[tiab] OR nasal catarrh*[tiab]
9	1 OR 2 OR 3
10	4 OR 5 OR 6 OR 7 OR 8
11	9 AND 10

Full query

(Birth Order[mh] OR Parity[mh] OR birth order*[tiab] OR multiple birth*[tiab] OR birth rank*[tiab] OR parity[tiab] OR Family Characteristics[mh] OR Family Health[mh] OR family characteristic*[tiab] OR family size*[tiab] OR family structure*[tiab] OR family demograph*[tiab] OR family composition[tiab] OR household size*[tiab] OR household demograph*[tiab] OR household composition[tiab] OR Siblings[mh] OR Sibling Relations[mh] OR sibling*[tiab] OR sister*[tiab] OR brother*[tiab] OR sibship size*[tiab] OR sibship*[tiab]) AND (Asthma[mh] OR Asthma, Exercise-Induced[mh] OR Respiratory Hypersensitivity[mh] OR bronchial asthma*[tiab] OR exercise-induced asthma*[tiab] OR exercise-induced bronchospasm*[tiab] OR asthma*[tiab] OR respiratory hypersensitivit*[tiab] OR airway hyper responsiveness[tiab] OR airway hyper-responsiveness[tiab] OR respiratory hyper responsiveness[tiab] OR respiratory hyper-responsiveness[tiab] OR wheez*[tiab] OR Hypersensitivity[mh] OR Hypersensitivity, Immediate[mh] OR Hypersensitivity, Delayed[mh] OR Allergy and Immunology[mh] OR Allergens / Immunology[mh] OR immediate hypersensitivit*[tiab] OR delayed hypersensitivit*[tiab] OR hypersensitivit*[tiab] OR IgE-mediated hypersensitivit*[tiab] OR type I hypersensitivit*[tiab] OR type IV hypersensitivit*[tiab] OR atopic sensitization[tiab] OR atop*[tiab] OR allergic sensitization[tiab] OR allergic disease*[tiab] OR allerg*[tiab] OR Dermatitis, Atopic[mh] OR Eczema[mh] OR Angioedema[mh] OR Anaphylaxis[mh] OR Urticaria[mh] OR atopic dermatitis[tiab] OR dermatitis[tiab] OR atopic eczema[tiab] OR eczema[tiab] OR neurodermatiti*[tiab]OR besnier's prurigo[tiab] OR besniers prurigo[tiab] OR besnier prurigo[tiab] OR urticari*[tiab] OR anaphyla*[tiab] OR quincke edema[tiab] OR quinckes edema[tiab] OR quincke's edema[tiab] OR angioneurotic edema[tiab] OR angioedema[tiab] OR hives[tiab]

For peer review only

OR Food Hypersensitivity[mh] OR food hypersensitivit*[tiab] OR food allerg*[tiab] OR egg allerg*[tiab] OR egg hypersensitivit*[tiab] OR milk allerg*[tiab] OR milk hypersensitivit*[tiab] OR shellfish allerg*[tiab] OR shellfish hypersensitivit*[tiab] OR wheat allerg*[tiab] OR wheat hypersensitivit*[tiab] OR nut allerg*[tiab] OR nut hypersensitivit*[tiab] OR peanut allerg*[tiab] OR peanut hypersensitivit*[tiab] OR groundnut hypersensitivit*[tiab] OR Pollen / Immunology[mh] OR Rhinitis, Allergic[mh] OR Rhinitis, Allergic, Seasonal[mh] OR Rhinitis, Allergic, Perennial[mh] OR Rhinitis[mh] OR Conjunctivitis, Allergic[mh] OR Conjunctivitis / Immunology[mh] OR Conjunctivitis / Epidemiology[mh] OR Conjunctivitis / Etiology[mh] OR allergic rhinoconjunctiviti*[tiab] OR rhinoconjunctiviti*[tiab] OR allergic rhiniti*[tiab] OR rhiniti*[tiab] OR seasonal allergic rhiniti*[tiab] OR perennial allergic rhiniti*[tiab] OR allergic conjunctiviti*[tiab] OR vernal keratoconjunctivitis*[tiab] OR vernal conjunctiviti*[tiab] OR giant papillary conjunctiviti*[tiab] OR hay fever[tiab] OR hayfever[tiab] OR pollinosis[tiab] OR pollenosis[tiab] OR nasal catarrh*[tiab])

mh = MeSH; tiab = title, abstract

Scopus

#	**Search term(s)**
1	TITLE-ABS-KEY(”birth order*”) OR TITLE-ABS-KEY(”multiple birth*”) OR TITLE-ABS-KEY(”birth rank*”) OR TITLE-ABS-KEY(”parity”)
2	TITLE-ABS-KEY(”family characteristic*”) OR TITLE-ABS-KEY(”family size*”) OR TITLE-ABS-KEY(”family structure*”) OR TITLE-ABS-KEY(”family demograph*”) OR TITLE-ABS-KEY(”family composition”) OR TITLE-ABS-KEY(”household size*”) OR TITLE-ABS-KEY(”household demograph*”) OR TITLE-ABS-KEY(”household composition*”)
3	TITLE-ABS-KEY(”sibling relation*”) OR TITLE-ABS-KEY(”sibling*”) OR TITLE-ABS-KEY(”brother*”) OR TITLE-ABS-KEY(”sister*”) OR TITLE-ABS-KEY(”sibship size*”) OR TITLE-ABS-KEY(”sibship*”)
4	TITLE-ABS-KEY(”bronchial asthma*”) OR TITLE-ABS-KEY(”exercise-induced asthma*”) OR TITLE-ABS-KEY(”asthma*”) OR TITLE-ABS-KEY(”exercise-induced bronchospasm*”) OR TITLE-ABS-KEY(”respiratory hypersensitivit*”) OR TITLE-ABS-KEY(”airway hyper responsiveness”) OR TITLE-ABS-KEY(”airway hyper-responsiveness”) OR TITLE-ABS-KEY(”respiratory hyper responsiveness”) OR TITLE-ABS-KEY(”respiratory hyper-responsiveness”) OR TITLE-ABS-KEY(”wheez*”)
5	TITLE-ABS-KEY(”immediate hypersensitivit*”) OR TITLE-ABS-KEY(”delayed hypersensitivit*”) OR TITLE-ABS-KEY(”IgE-mediated hypersensitivit*”) OR TITLE-ABS-KEY(”type I hypersensitivit*”) OR TITLE-ABS-KEY(”type IV hypersensitivit*”) OR TITLE-ABS-KEY(”atopic sensitization”) OR TITLE-ABS-KEY(”atop*”) OR TITLE-ABS-KEY(”allergic sensitization”) OR TITLE-ABS-KEY(”allergic disease”) OR TITLE-ABS-KEY(”allerg*”)
6	TITLE-ABS-KEY(”atopic dermatitis”) OR TITLE-ABS-KEY(”dermatitis”) OR TITLE-ABS-KEY(”atopic eczema”) OR TITLE-ABS-KEY(”eczema”) OR TITLE-ABS-KEY(”neurodermatitis*”) OR TITLE-ABS-KEY(”besnier’s prurigo”) OR TITLE-ABS-KEY(”besniers prurigo”) OR TITLE-ABS-KEY(”besnier prurigo”) OR TITLE-ABS-KEY(”quincke’s edema”) OR TITLE-ABS-KEY(”quinckes edema”) OR TITLE-ABS-KEY(”quincke edema”) OR TITLE-ABS-KEY(”angioneurotic edema”) OR TITLE-ABS-KEY(”hives”) OR TITLE-ABS-KEY(”anaphyla*”) OR TITLE-ABS-KEY(”urticari*”)

For peer review only

7	TITLE-ABS-KEY(”food hypersensitivit*”) OR TITLE-ABS-KEY(”food allerg*”) OR TITLE-ABS-KEY(”egg allerg*”) OR TITLE-ABS-KEY(”egg hypersensitivit*”) OR TITLE-ABS-KEY(”milk allerg*”) OR TITLE-ABS-KEY(”milk hypersensitivit*”) OR TITLE-ABS-KEY(”shellfish allerg*”) OR TITLE-ABS-KEY(”shellfish hypersensitivit*”) OR TITLE-ABS-KEY(”wheat allerg*”) OR TITLE-ABS-KEY(”wheat hypersensitivit*”) OR TITLE-ABS-KEY(”nut allerg*”) OR TITLE-ABS-KEY(”nut hypersensitivit*”) OR TITLE-ABS-KEY(”peanut allerg*”) OR TITLE-ABS-KEY(”peanut hypersensitivit*”) OR TITLE-ABS-KEY(”groundnut allerg*”) OR TITLE-ABS-KEY(”groundnut hypersensitivit*”)
8	TITLE-ABS-KEY(”allergic rhinoconjunctiviti*”) OR TITLE-ABS-KEY(”rhinoconjunctiviti*”) OR TITLE-ABS-KEY(”seasonal allergic rhiniti*”) OR TITLE-ABS-KEY(”perennial allergic rhiniti*”) OR TITLE-ABS-KEY(”allergic rhiniti*”) OR TITLE-ABS-KEY(”rhiniti*”) OR TITLE-ABS-KEY(”allergic conjunctiviti*”) OR TITLE-ABS-KEY(”vernal keratoconjunctiviti*”) OR TITLE-ABS-KEY(”vernal conjunctiviti*”) OR TITLE-ABS-KEY(”giant papillary conjunctiviti*”) OR TITLE-ABS-KEY(”hay fever”) OR TITLE-ABS-KEY(”hayfever”) OR TITLE-ABS-KEY(”pollinosis”) OR TITLE-ABS-KEY(”pollenosis”) OR TITLE-ABS-KEY(”nasal catarrh*”)
9	1 OR 2 OR 3
10	4 OR 5 OR 6 OR 7 OR 8
11	9 AND 10

Full query

(TITLE-ABS-KEY(”birth order*”) OR TITLE-ABS-KEY(”multiple birth*”) OR TITLE-ABS-KEY(”birth rank*”) OR TITLE-ABS-KEY(”parity”) OR TITLE-ABS-KEY(”family characteristic*”) OR TITLE-ABS-KEY(”family size*”) OR TITLE-ABS-KEY(”family structure*”) OR TITLE-ABS-KEY(”family demograph*”) OR TITLE-ABS-KEY(”family composition”) OR TITLE-ABS-KEY(”household size*”) OR TITLE-ABS-KEY(”household demograph*”) OR TITLE-ABS-KEY(”household composition*”) OR TITLE-ABS-KEY(”sibling relation*”) OR TITLE-ABS-KEY(”sibling*”) OR TITLE-ABS-KEY(”brother*”) OR TITLE-ABS-KEY(”sister*”) OR TITLE-ABS-KEY(”sibship size*”) OR TITLE-ABS-KEY(”sibship*”)) AND (TITLE-ABS-KEY(”bronchial asthma*”) OR TITLE-ABS-KEY(”exercise-induced asthma*”) OR TITLE-ABS-KEY(”asthma*”) OR TITLE-ABS-KEY(”exercise-induced bronchospasm*”) OR TITLE-ABS-KEY(”respiratory hypersensitivit*”) OR TITLE-ABS-KEY(”airway hyper responsiveness”) OR TITLE-ABS-KEY(”airway hyper-responsiveness”) OR TITLE-ABS-KEY(”respiratory hyper responsiveness”) OR TITLE-ABS-KEY(”respiratory hyper-responsiveness”) OR TITLE-ABS-KEY(”wheez*”) OR TITLE-ABS-KEY(”immediate hypersensitivit*”) OR TITLE-ABS-KEY(”delayed hypersensitivit*”) OR TITLE-ABS-KEY(”IgE-mediated hypersensitivit*”) OR TITLE-ABS-KEY(”type I hypersensitivit*”) OR TITLE-ABS-KEY(”type IV hypersensitivit*”) OR TITLE-ABS-KEY(”atopic sensitization”) OR TITLE-ABS-KEY(”atop*”) OR TITLE-ABS-KEY(”allergic sensitization”) OR TITLE-ABS-KEY(”allergic disease”) OR TITLE-ABS-KEY(”allerg*”) OR TITLE-ABS-KEY(”atopic dermatitis”) OR TITLE-ABS-KEY(”dermatitis”) OR TITLE-ABS-KEY(”atopic eczema”) OR TITLE-ABS-KEY(”eczema”) OR TITLE-ABS-KEY(”neurodermatitis*”) OR TITLE-ABS-KEY(”besnier’s prurigo”) OR TITLE-ABS-KEY(”besniers prurigo”) OR TITLE-ABS-KEY(”besnier prurigo”) OR TITLE-ABS-KEY(”quincke’s edema”) OR TITLE-ABS-KEY(”quinckes edema”) OR TITLE-ABS-KEY(”quincke edema”) OR TITLE-ABS-KEY(”angioneurotic edema”) OR TITLE-ABS-KEY(”hives”) OR TITLE-ABS-KEY(”anaphyla*”) OR TITLE-ABS-KEY(”urticari*”) OR TITLE-ABS-KEY(”food hypersensitivit*”) OR TITLE-ABS-KEY(”food allerg*”) OR TITLE-ABS-KEY(”egg allerg*”) OR TITLE-ABS-KEY(”egg hypersensitivit*”) OR TITLE-ABS-KEY(”milk allerg*”) OR TITLE-ABS-KEY(”milk hypersensitivit*”) OR TITLE-ABS-KEY(”shellfish allerg*”) OR TITLE-ABS-KEY(”shellfish

For peer review only

hypersensitivit*") OR TITLE-ABS-KEY("wheat allerg*") OR TITLE-ABS-KEY("wheat hypersensitivit*") OR TITLE-ABS-KEY("nut allerg*") OR TITLE-ABS-KEY("nut hypersensitivit*") OR TITLE-ABS-KEY("peanut allerg*") OR TITLE-ABS-KEY("peanut hypersensitivit*") OR TITLE-ABS-KEY("groundnut allerg*") OR TITLE-ABS-KEY("groundnut hypersensitivit*") OR TITLE-ABS-KEY("allergic rhinoconjunctiviti*") OR TITLE-ABS-KEY("rhinoconjunctiviti*") OR TITLE-ABS-KEY("seasonal allergic rhiniti*") OR TITLE-ABS-KEY("perennial allergic rhiniti*") OR TITLE-ABS-KEY("allergic rhiniti*") OR TITLE-ABS-KEY("rhiniti*") OR TITLE-ABS-KEY("allergic conjunctiviti*") OR TITLE-ABS-KEY("vernal keratoconjunctiviti*") OR TITLE-ABS-KEY("vernal conjunctiviti*") OR TITLE-ABS-KEY("giant papillary conjunctiviti*") OR TITLE-ABS-KEY("hay fever") OR TITLE-ABS-KEY("hayfever") OR TITLE-ABS-KEY("pollinosis") OR TITLE-ABS-KEY("pollenosis") OR TITLE-ABS-KEY("nasal catarrh*"))

TITLE-ABS-KEY = title, abstract, keywords

Web of Science

#	**Search term(s)**
1	TS="birth order*" OR TS="multiple birth*" OR TS="birth rank*" OR TS="parity"
2	TS="family characteristic*" OR TS="family size*" OR TS="family structure*" OR TS="family demograph*" OR TS="family composition" OR TS="household size*" OR TS="household demograph*" OR TS="household composition"
3	TS="sibling*" OR TS="sister*" OR TS="brother*" OR TS="sibship size*" OR TS="sibship*"
4	TS="bronchial asthma*" OR TS="exercise-induced asthma*" OR TS="exercise-induced bronchospasm*" OR TS="asthma*" OR TS="respiratory hypersensitivit*" OR TS="respiratory hyper-responsiveness*" OR TS="airway hyper responsiveness*" OR TS="airway hyper-responsiveness*" OR TS=wheez*
5	TS="immediate hypersensitivit*" OR TS="delayed hypersensitivit*" OR TS="IgE-mediated hypersensitivit*" OR TS="type I hypersensitivit*" OR TS="type IV hypersensitivit*" OR TS="hypersensitivit*" OR TS="atopic sensitization" OR TS="atop*" OR TS="allergic sensitization" OR TS="allergic disease*" OR TS="allerg*"
6	TS="atopic dermatitis" OR TS="dermatitis" OR TS="atopic eczema" OR TS="eczema" OR TS="neurodermatiti*" OR TS="besnier's prurigo" OR TS="besniers prurigo" OR TS="besnier prurigo" OR TS="urticari*" OR TS="anaphyla*" OR TS="quincke's edema" OR TS="quinckes edema" OR TS="quincke edema" OR TS="angioneurotic edema" OR TS="angioedema" OR TS="hives"
7	TS="food hypersensitivit*" OR TS="food allerg*" OR TS="egg allerg*" OR TS="egg hypersensitivit*" OR TS="milk allerg*" OR TS="milk hypersensitivit*" OR TS="shellfish allerg*" OR TS="shellfish hypersensitivit*" OR TS="wheat allerg*" OR TS="wheat hypersensitivit*" OR TS="nut allerg*" OR TS="nut hypersensitivit*" OR TS="peanut allerg*" OR TS="peanut hypersensitivit*" OR TS="groundnut allerg*" OR TS="groundnut hypersensitivit*"
8	TS="pollen allerg*" OR TS="allergic rhinoconjunctiviti*" OR TS="rhinoconjunctiviti*" OR TS="seasonal allergic rhiniti*" OR TS="perennial allergic rhiniti*" OR TS="allergic rhiniti*" OR TS="rhiniti*" OR TS="allergic conjunctiviti*" OR TS="vernal keratoconjunctiviti*" OR

For peer review only

	TS=”vernal conjunctiviti*” OR TS=”giant papillary conjunctiviti*” OR TS=”hay fever” OR TS=”hayfever” OR TS=”pollinosis” OR TS=”pollenosis” OR TS=”nasal catarrh*”
9	1 OR 2 OR 3
10	4 OR 5 OR 6 OR 7 OR 8
11	9 AND 10

Full query

(TS="birth order*" OR TS="multiple birth*" OR TS="birth rank*" OR TS="parity" OR TS="family characteristic*" OR TS="family size*" OR TS="family structure*" OR TS="family demograph*" OR TS="family composition" OR TS="household size*" OR TS="household demograph*" OR TS="household composition" OR TS="sibling*" OR TS="sister*" OR TS="brother*" OR TS="sibship size*" OR TS="sibship*") AND (TS="bronchial asthma*" OR TS="exercise-induced asthma*" OR TS="exercise-induced bronchospasm*" OR TS="asthma*" OR TS="respiratory hypersensitivit*" OR TS="respiratory hyper-responsiveness*" OR TS="airway hyper responsiveness*" OR TS="airway hyper-responsiveness*" OR TS=wheez* OR TS="immediate hypersensitivit*" OR TS="delayed hypersensitivit*" OR TS="IgE-mediated hypersensitivit*" OR TS="type I hypersensitivit*" OR TS="type IV hypersensitivit*" OR TS="hypersensitivit*" OR TS="atopic sensitization" OR TS="atop*" OR TS="allergic sensitization" OR TS="allergic disease*" OR TS="allerg*" OR TS="atopic dermatitis" OR TS="dermatitis" OR TS="atopic eczema" OR TS="eczema" OR TS="neurodermatiti*" OR TS="besnier’s prurigo" OR TS="besniers prurigo" OR TS="besnier prurigo" OR TS="urticari*" OR TS="anaphyla*" OR TS="quincke’s edema" OR TS="quinckes edema" OR TS="quincke edema" OR TS="angioneurotic edema" OR TS="angioedema" OR TS="hives" OR TS="food hypersensitivit*" OR TS="food allerg*" OR TS="egg allerg*" OR TS="egg hypersensitivit*" OR TS="milk allerg*" OR TS="milk hypersensitivit*" OR TS="shellfish allerg*" OR TS="shellfish hypersensitivit*" OR TS="wheat allerg*" OR TS="wheat hypersensitivit*" OR TS="nut allerg*" OR TS="nut hypersensitivit*" OR TS="peanut allerg*" OR TS="peanut hypersensitivit*" OR TS="groundnut allerg*" OR TS="groundnut hypersensitivit*" OR TS="pollen allerg*" OR TS="allergic rhinoconjunctiviti*" OR TS="rhinoconjunctiviti*" OR TS="seasonal allergic rhiniti*" OR TS="perennial allergic rhiniti*" OR TS="allergic rhiniti*" OR TS="rhiniti*" OR TS="allergic conjunctiviti*" OR TS="vernal keratoconjunctiviti*" OR TS="vernal conjunctiviti*" OR TS="giant papillary conjunctiviti*" OR TS="hay fever" OR TS="hayfever" OR TS="pollinosis" OR TS="pollenosis" OR TS="nasal catarrh*")

TS = title, abstract, author keywords, Keywords Plus

For peer review only

Appendix to

Sibship Size, Birth Order and Risk of Asthma and Allergy: Protocol for a Systematic Review and Meta-Analysis

Daniil Lisik, Saliha Selin Özuygur Ermis, Emma Goksör, Göran Wennergren, Bright I Nwaru

Appendix 2: Data Extraction Form

Reviewer (initials)	
Date of data extraction (yyyy-mm-dd)	
**General information**	
Author (for first author: surname, given name(s))	
Title of article	
Year of publication	
Country of origin of study	
Contact information to author(s)	
**Study characteristics**	
Study design	
Study aims/objectives	
Exposure(s) (for each exposure: 1) method(s) of assessment; 2) objectivity of assessment (objective/subjective[1]); 3) validity (yes[2]/no); 4) reliability (yes[3]/no))	
Outcome(s) (for each outcome: 1) method(s) of assessment; 2) objectivity (objective/subjective[4]); 3) validity (yes[2]/no); 4) reliability (yes[3]/no))	
Follow-up (method; length)	Method: ;<br>Length:<br><br>Or: ☐ not applicable
Study was conducted during (year(s))	
**Participant selection**	
Inclusion criteria	
Exclusion criteria	
Source(s) of subjects	
**Population characteristics**	
Participants recruited (n; details)	n: ;<br>Details:

For peer review only

Participants eligible (n; % of "Participants recruited"; eligibility criteria)	n: ;<br>% of "Participants recruited": %;<br>Eligibility criteria:
Participants included (n; % of "Participants eligible")	n: ;<br>% of "Participants eligible": %<br><br>Or:
Participants completing follow-up (n; % of "Participants included")	n: ;<br>% of "Participants included": %<br><br>Or:
Participants lost (n; % of "Participants included"; details; how it was dealt with)	n: ;<br>% of "Participants included": %;<br>Details: ;<br>How it was dealt with:<br><br>Or:
Data lost (n; % of "Participants included"; details; how it was dealt with)	n: ;<br>% of "Participants included": %;<br>Details: ;<br>How it was dealt with:
Participants characteristics (for each group: 1) n_{total}; 2) age (mean (SD)); 3) gender distribution (n_{males} (% of n_{total})); 4) ethnicity; 5) country; 6) economic classification of country by the World Bank; 7) setting; 8) co-morbidity)	
**Results**	
Outcomes (for each outcome for each exposure, stratified by group[5] if applicable: 1) n (% of n_{group}); 2) effect size (measure of effect) 95% CI; 3) p-value)	
**Analysis**	
Statistical analysis method	
Confounders (what confounders were identified; how they were controlled for; % of confounders controlled for)	Confounders identified: ;<br>How they were controlled for:<br><br>% of confounders controlled for:
Free-text interpretation of findings/conclusion	
Generalizability (is it likely that individuals selected for this study to be representative of the target population?)	
**Miscellaneous**	

For peer review only

Other comments/notes	
**Quality assessment (based on the Effective Public Health Practice Project (EPHPP) quality assessment tool[6])**	
**(A) Selection bias**	
(Q1) Is it likely that individuals selected for this study to be representative of the target population?	
(Q2) How many of eligible individuals agreed to participate in the study? (%)	
Section rating	
**(B) Study design**	
Indicate the design of the study	
Was the study setting randomized? If "No", go to (C)	
If "Yes", was the randomization method described?	
If "Yes", was the method referred to above appropriate?	
Section rating<br>Rate longitudinal studies as "moderate", and cross-sectional studies as "weak"	
**(C) Confounders**	
(Q1) Did the groups have significant differences in relation to each other prior to the intervention?	
(Q2) If "Yes", indicate how many relevant confounders that were controlled for in any way (e.g. in study design through matching, stratification, or in analysis) (%)	
Section rating<br>Rate studies without a control group as weak	
**(D) Blinding**	
(Q1) Did the outcome assessor(s) know about the exposure status of the participants?	
(Q2) Did the participants of the study know about the research question?	
Section rating<br>Rate as "weak" if Q1 is 1 and Q2 is 3	
**(E) Data collection methods**	
(Q1) Were the tools used for data collection shown to be valid?	

For peer review only

(Q2) Were the tools used for data collection shown to be reliable?	
Section rating	
**(F) Withdrawals and drop-outs**	
(Q1) Did numbers and/or reasons for withdrawals and drop-outs per group get documented?	
(Q2) How many participants completed the study (if the value is different between groups, state the lowest)? (%)	
Section rating	
**Global rating**	
Did the two reviewers give different section ratings for A-F?	
If "Yes", what is the reason for the difference(s)?	
**Final rating of both reviewers**	

[1] Objective: medical records/official statistics. Subjective: self-report, observation.
[2] Yes: The assessment gives usable, meaningful information for the research question.
[3] Yes: Results from assessment type are consistent and stable.
[4] Objective: ICD code, verified diagnosis based on medical examination. Subjective: otherwise observed or self-reported symptoms/disease.
[5] Applicable if specific stratification has been made in the analysis, e.g. significant differences and/or calculations based on gender of participants.
[6] Modified version of EPHPP [1]. Original tool is available at: https://www.ephpp.ca/quality-assessment-tool-for-quantitative-studies/. The questions and answer alternatives are modified in phrasing for readability and the nature of relevant studies. Modifications of rating are clarified in green text above, based on the modifications of EPHPP done in a systematic review by Smith et al. in 2017 [2].

References

1. Armijo-Olivo S, Stiles CR, Hagen NA, Biondo PD, Cummings GG. Assessment of study quality for systematic reviews: a comparison of the Cochrane Collaboration Risk of Bias Tool and the Effective Public Health Practice Project Quality Assessment Tool: methodological research. J Eval Clin Pract. 2012;18(1):12-18.
2. Smith M, Hosking J, Woodward A, Witten K, MacMillan A, Field A, et al. Systematic literature review of built environment effects on physical activity and active transport – an update and new findings on health equity. International Journal of Behavioral Nutrition and Physical Activity. 2017;14(1):158.

For peer review only

Appendix to

Sibship Size, Birth Order and Risk of Asthma and Allergy: Protocol for a Systematic Review and Meta-Analysis

Daniil Lisik, Saliha Selin Özuygur Ermis, Emma Goksör, Göran Wennergren, Bright I Nwaru

Appendix 3: PRISMA-P Checklist

For peer review only

PRISMA-P (Preferred Reporting Items for Systematic review and Meta-Analysis Protocols) 2015 checklist: recommended items to address in a systematic review protocol*

Section and topic	Item No	Checklist item	Reported on page
**ADMINISTRATIVE INFORMATION**			
Title:			
Identification	1a	Identify the report as a protocol of a systematic review	**1**
Update	1b	If the protocol is for an update of a previous systematic review, identify as such	**N/A**
Registration	2	If registered, provide the name of the registry (such as PROSPERO) and registration number	**2, 4 (awaiting registration number)**
Authors:			
Contact	3a	Provide name, institutional affiliation, e-mail address of all protocol authors; provide physical mailing address of corresponding author	**1**
Contributions	3b	Describe contributions of protocol authors and identify the guarantor of the review	**9-10**
Amendments	4	If the protocol represents an amendment of a previously completed or published protocol, identify as such and list changes; otherwise, state plan for documenting important protocol amendments	**N/A**
Support:			
Sources	5a	Indicate sources of financial or other support for the review	**10**
Sponsor	5b	Provide name for the review funder and/or sponsor	**N/A**
Role of sponsor or funder	5c	Describe roles of funder(s), sponsor(s), and/or institution(s), if any, in developing the protocol	**N/A**
**INTRODUCTION**			
Rationale	6	Describe the rationale for the review in the context of what is already known	**2-3**
Objectives	7	Provide an explicit statement of the question(s) the review will address with reference to participants, interventions, comparators, and outcomes (PICO)	**3-4**

METHODS			
Eligibility criteria	8	Specify the study characteristics (such as PICO, study design, setting, time frame) and report characteristics (such as years considered, language, publication status) to be used as criteria for eligibility for the review	**4-5**
Information sources	9	Describe all intended information sources (such as electronic databases, contact with study authors, trial registers or other grey literature sources) with planned dates of coverage	**5-6**
Search strategy	10	Present draft of search strategy to be used for at least one electronic database, including planned limits, such that it could be repeated	**5-6, Appendix 1**
Study records:			
Data management	11a	Describe the mechanism(s) that will be used to manage records and data throughout the review	**6**
Selection process	11b	State the process that will be used for selecting studies (such as two independent reviewers) through each phase of the review (that is, screening, eligibility and inclusion in meta-analysis)	**6**
Data collection process	11c	Describe planned method of extracting data from reports (such as piloting forms, done independently, in duplicate), any processes for obtaining and confirming data from investigators	**6-7, Appendix 2**
Data items	12	List and define all variables for which data will be sought (such as PICO items, funding sources), any pre-planned data assumptions and simplifications	**7**
Outcomes and prioritization	13	List and define all outcomes for which data will be sought, including prioritization of main and additional outcomes, with rationale	**4-5**
Risk of bias in individual studies	14	Describe anticipated methods for assessing risk of bias of individual studies, including whether this will be done at the outcome or study level, or both; state how this information will be used in data synthesis	**7**
Data synthesis	15a	Describe criteria under which study data will be quantitatively synthesised	**7-8**
	15b	If data are appropriate for quantitative synthesis, describe planned summary measures, methods of handling data and methods of combining data from studies, including any planned exploration of consistency (such as I^2, Kendall's τ)	**7-8**
	15c	Describe any proposed additional analyses (such as sensitivity or subgroup analyses, meta-regression)	**8**
	15d	If quantitative synthesis is not appropriate, describe the type of summary planned	**7**
Meta-bias(es)	16	Specify any planned assessment of meta-bias(es) (such as publication bias across studies, selective reporting within studies)	**8**
Confidence in cumulative evidence	17	Describe how the strength of the body of evidence will be assessed (such as GRADE)	**8**

*** It is strongly recommended that this checklist be read in conjunction with the PRISMA-P Explanation and Elaboration (cite when available) for important clarification on the items.**

For peer review only

Amendments to a review protocol should be tracked and dated. The copyright for PRISMA-P (including checklist) is held by the PRISMA-P Group and is distributed under a Creative Commons Attribution Licence 4.0.

From: Shamseer L, Moher D, Clarke M, Ghersi D, Liberati A, Petticrew M, Shekelle P, Stewart L, PRISMA-P Group. Preferred reporting items for systematic review and meta-analysis protocols (PRISMA-P) 2015: elaboration and explanation. BMJ. 2015 Jan 2;349(jan02 1):g7647.

For peer review only

Sibship Size, Birth Order and Risk of Asthma and Allergy: Protocol for a Systematic Review and Meta-Analysis

Journal:	*BMJ Open*
Manuscript ID	bmjopen-2020-045795.R1
Article Type:	Protocol
Date Submitted by the Author:	30-Jul-2021
Complete List of Authors:	Lisik, Daniil; University of Gothenburg, Krefting Research Centre<br>Ioannidou, Athina; University of Gothenburg<br>Milani, Gregorio; Ospedale Maggiore Policlinico, Pediatric unit<br>Nyassi, Sungkutu; University of Gothenburg<br>Özuygur Ermis, Saliha Selin; Dokuz Eylül University, Department of Pulmonology<br>Spolidoro, Giulia C. I.; University of Milan, Department of Clinical Sciences and Community Health<br>Goksör, Emma; University of Gothenburg<br>Wennergren, Göran; University of Gothenburg Sahlgrenska Academy, Krefting Research Centre; University of Gothenburg Sahlgrenska Academy, Dept of Paediatrics<br>Nwaru, Bright; University of Gothenburg, Krefting Research Centre
**Primary Subject Heading**:	Immunology (including allergy)
Secondary Subject Heading:	Epidemiology
Keywords:	Asthma < THORACIC MEDICINE, Allergy < THORACIC MEDICINE, Eczema < DERMATOLOGY, IMMUNOLOGY, Paediatric infectious disease & immunisation < PAEDIATRICS, Paediatric dermatology < PAEDIATRICS

SCHOLARONE™ Manuscripts

I, the Submitting Author has the right to grant and does grant on behalf of all authors of the Work (as defined in the below author licence), an exclusive licence and/or a non-exclusive licence for contributions from authors who are: i) UK Crown employees; ii) where BMJ has agreed a CC-BY licence shall apply, and/or iii) in accordance with the terms applicable for US Federal Government officers or employees acting as part of their official duties; on a worldwide, perpetual, irrevocable, royalty-free basis to BMJ Publishing Group Ltd ("BMJ") its licensees and where the relevant Journal is co-owned by BMJ to the co-owners of the Journal, to publish the Work in this journal and any other BMJ products and to exploit all rights, as set out in our licence.

The Submitting Author accepts and understands that any supply made under these terms is made by BMJ to the Submitting Author unless you are acting as an employee on behalf of your employer or a postgraduate student of an affiliated institution which is paying any applicable article publishing charge ("APC") for Open Access articles. Where the Submitting Author wishes to make the Work available on an Open Access basis (and intends to pay the relevant APC), the terms of reuse of such Open Access shall be governed by a Creative Commons licence – details of these licences and which Creative Commons licence will apply to this Work are set out in our licence referred to above.

Other than as permitted in any relevant BMJ Author's Self Archiving Policies, I confirm this Work has not been accepted for publication elsewhere, is not being considered for publication elsewhere and does not duplicate material already published. I confirm all authors consent to publication of this Work and authorise the granting of this licence.

For peer review only

Sibship Size, Birth Order and Risk of Asthma and Allergy: Protocol for a Systematic Review and Meta-Analysis

Daniil Lisik[3], Athina Ioannidou[3], Gregorio Paolo Milani[4], Sungkutu Nyassi[3], Saliha Selin Özuygur Ermis[1], Giulia Spolidoro[4], Emma Goksör[2], Göran Wennergren[2], Bright I Nwaru[3]

Author affiliations

[1] Department of Pulmonology, Dokuz Eylul University, Izmir, Turkey

[2] Department of Pediatrics, University of Gothenburg, Queen Silvia Children's Hospital, Gothenburg, Sweden.

[3] Krefting Research Centre, Institute of Medicine, University of Gothenburg, Sweden

[4] Department of Clinical Sciences and Community Health, Università degli Studi di Milano Statale, Italy

Correspondence to

Daniil Lisik
Institute of Medicine
Sahlgrenska Academy
University of Gothenburg, Sweden
daniil.lisik@gmail.com

For peer review only

ABSTRACT

Introduction: The hygiene hypothesis suggests that reduced exposure to microbes might have contributed to the increase in prevalence and incidence of asthma and allergy observed during the second half of the last century. Following this proposal, several studies have investigated the role of sibship size and birth order in the development of asthma and allergic diseases, but the underlying evidence is conflicting. The objective of the present systematic review will be to identify, critically appraise and synthesize previous primary studies investigating the association of sibship size and birth order with the risk of asthma and allergic diseases.

Methods and analysis: The following databases will be searched: AMED, CABI, CINAHL, Embase, Google Scholar, OAIster, Open Access Theses and Dissertations, Open Grey,

For peer review only - http://bmjopen.bmj.com/site/about/guidelines.xhtml

ProQuest Dissertations & Theses Global, PsycINFO, PubMed, SciELO, Scopus, Web of Science, and WHO Global Index Medicus. Studies published up until 31st December 2020 will be eligible. There will be no restrictions by language and geographical location. Risk of bias in the included studies will be assessed using the Effective Public Health Practice Project (EPHPP) quality assessment tool. The produced evidence will be synthesized narratively, and studies that present comparable numerical data will be included in meta-analyses using random-effects model.

Ethics and dissemination: Only data from the published literature will be included in this systematic review. Therefore, no ethical approval is required. The final review paper will be published in a peer-reviewed journal.

PROSPERO registration number: CRD42020207905.

Strengths and limitations of this study

- This will be the first systematic review encompassing a comprehensive spectrum of the most common allergic and respiratory outcomes, in relation to sibship size and birth order.
- Inclusion of the leading databases, including search of the gray literature, enables a comprehensive identification of the relevant studies addressing the research question.
- The reproducibility of our work is enhanced through a priori outline of the review processes before the actual review starts.
- Self-reported diagnoses of the study outcomes are expected to make up a significant source of data from included studies, which gives the possibility of assessment bias.

INTRODUCTION

The incidence and prevalence of asthma, along with allergic diseases such as allergic rhinitis and atopic eczema, were observed to have increased during the second half of the last century, in particular in the developed world [1, 2]. More recent trends remain unclear, as both increase [3] and leveling off [1, 4, 5] have been suggested. Around 300 million people

For peer review only - http://bmjopen.bmj.com/site/about/guidelines.xhtml

have asthma globally [1]. For allergic diseases, evidence indicates that there is still a global increase in prevalence [5, 6]. Asthma and allergic diseases account for significant morbidity for individuals, as well as a substantial socio-economic burden on the society [2]. Asthma results in roughly 14 million missed school days each year in the United States alone, and the morbidity is even higher for adults [7]. Allergic rhinitis is also associated with significant loss in productivity [8]. Furthermore, the World Health Organization estimates that roughly 250,000 cases of death annually, worldwide, are due to asthma [7]. Identifying risk factors for asthma and allergy is therefore of great interest, in order to reduce the burden associated with these diseases.

Over the last five decades, numerous hypotheses have been proposed to explain the observed increase in the prevalence of these diseases, a substantial part of the studies focusing on the role of environmental factors. One of the main hypotheses is the hygiene hypothesis, which was first proposed by Strachan in 1989, and suggests that reduced microbial exposure during childhood increases the risk of developing asthma and allergy [9]. One of the first proposed underlying biological mechanisms to the hygiene hypothesis was the observed skewing of balance towards T helper 2 cells (which have been associated with allergic sensitization) in subjects lacking microbial stimuli, and conversely a T helper 1 cell domination in subjects exposed to greater quantities of microbes [10]. Further research has broadened the explanatory model with additional factors, such as T regulatory cells and T helper 17 cells [11], but the pathophysiology is yet to be fully understood [12]. Connected to the hygiene hypothesis is the proposed sibling effect, which suggests that the number of siblings and/or the birth order of a child in a family may play a role in the development of asthma and allergy, as a result of varying degrees of microbial exposure during childhood, depending on the number of siblings in total and/or the number of younger/older siblings [13].

While several studies have investigated the association of sibship (group of individuals sharing the same pair of parents) size and birth order (the sequence in which members of a sibship are born) with risk of asthma and allergic diseases, findings are conflicting [14]. Karmaus and Botezan have estimated the proportion of cases attributable to the sibling effect to be 34% for atopic dermatitis, 56% for allergic rhinitis, and 28% for asthma. Karmaus and Botezan have also argued that at least 30% of cases of asthma and allergy could be prevented

For peer review only - http://bmjopen.bmj.com/site/about/guidelines.xhtml

For peer review only

if the causal factors for these conditions were better understood [15], further indicating warranty for elucidating the sibling effect in relation to asthma and allergy. So far, there are no systematic reviews synthesizing evidence from previous studies on the topic. A systematic synthesis of previous studies investigating the association of sibship size and birth order with risk of asthma and allergy will provide a clearer appreciation of the strength, magnitude, and quality of the underlying evidence.

Aim

To identify, critically appraise and synthesize previous primary studies investigating the association of sibship size and birth order with risk of asthma and allergic diseases.

METHODS AND ANALYSES

This protocol is reported according to the recommendations of the Preferred Reporting Items for Systematic Reviews and Meta-Analysis Protocol (PRISMA-P) [16], which provides guidelines for a standardized, transparent and reproducible reporting of systematic review protocols. The PRISMA-P checklist is presented in Appendix 1. Updates to the protocol will be documented, and deviations from the protocol will be described in the final review paper. The protocol for this systematic review has been prospectively registered with the international prospective register of systematic reviews (PROSPERO, https://www.crd.york.ac.uk/prospero/display_record.php?RecordID=207905) with registration number CRD42020207905.

Study eligibility criteria

Study types and publication status

We will include observational epidemiological studies, including prospective and retrospective cohort studies, case-control studies, and cross-sectional studies. Randomized controlled studies, quasi-randomized controlled studies, controlled before-after studies, and controlled clinical trials will not be considered, as interventional studies are not relevant for this research question. Animal studies, reviews, case studies, case series, expert opinions will also be excluded. Studies of any publication status will be eligible, and data used from them if available.

For peer review only - http://bmjopen.bmj.com/site/about/guidelines.xhtml

For peer review only

Participants

Offspring of any age, gender, ethnic background and medical background, where the study context is that the participants are part of defined sibships. Studies with any amount of participants will be eligible.

Exposures

Sibship size, birth order (number of older siblings), and number of younger siblings in the studied sibships.

Outcome measures

Self-reported or objectively measured/diagnosed asthma and allergic disease in the sibships. For the purpose of encompassing all relevant literature on the topic, asthma and allergic disease will be defined broadly. Asthma will be defined as any type of asthma, including those based on symptom definition, such as wheezing, and those based on spirometry findings of variable expiratory airflow limitation [17]. Allergic disease will encompass any of the following: (a) allergic rhinitis/(rhino)conjunctivitis, food allergy, atopic eczema, urticaria, angioedema, anaphylaxis [18]; (b) indicators of hypersensitivity (and indirectly of allergic disease), which includes allergen-specific serum immunoglobulin E (IgE) test, skin prick test (SPT), provocation/challenge test. Conditions with primarily a genetic etiology, such as hereditary angioedema [19], will not be included in these definitions.

Search methods

The search queries were developed using the PEO model: population, exposure and outcome (PEO). PEO is a specific implementation of PICO, used as a framework to produce effective search queries from formulated research questions, especially befitting retrieval of interventional and observational studies [20]. Since the population (P) will be defined broadly, i.e., including both studies in children and adults, the actual search queries will be composed of two blocks; exposure (E) and outcome (O). A scoping search was performed in PubMed to identify previous studies on the topic and map relevant search terms. The search terms identified were: Medical Subject Headings (MeSH), and their corresponding alternatives in other databases; entry terms; free-text words and phrases. Subsequent scoping

For peer review only - http://bmjopen.bmj.com/site/about/guidelines.xhtml

searches were made in PubMed with boolean operator "NOT" between various MeSH and free-text terms, alternately, in order to identify more synonyms and related search terms. The developed search terms have been piloted and refined before they will be used to identify relevant studies. The search queries have been modified for each database to be searched in regards to, inter alia, support for controlled vocabulary and syntax. Peer Review of Electronic Search Strategies (PRESS) has been used to identify potential weaknesses in the search strategy. Details of the search strategy are presented in Appendix 2.

Studies will be retrieved from the following databases: AMED (via Ovid), CABI, CINAHL (via EBSCO), Embase (via Ovid), Google Scholar, PsycINFO (via ProQuest), PubMed, SciELO, Scopus, Web of Science, and WHO Global Index Medicus. In addition, unpublished articles and gray literature will be retrieved through searches of OAIster, Open Access Theses and Dissertations, Open Grey, and ProQuest Dissertations & Theses Global. Finally, studies will also be included from reference lists of the studies included in the review, as well as through contact with experts who have published in the field. All databases will be searched for articles published from inception of respective database up until 31st December 2020; an updated search will be performed at the completion of the review to ensure inclusion of studies published after the first search. There will be no language restrictions, and articles will be translated into English where possible. Articles that could not be translated will be reported in the final review paper. In Google Scholar, due to the fact that the amount of results is sometimes overwhelming, results will be retrieved from the first 300 hits [21]. Furthermore, the search query for Google Scholar has been significantly simplified, including only the most important terms in each block, due to an upper limit of 256 characters for search strings [22].

Data management

EndNote will be used for de-duplication, full-text retrieval, secondary screening, and for general management of retrieved studies. For primary screening, the articles will be imported to Rayyan QCRI.

Screening/selection process

For peer review only - http://bmjopen.bmj.com/site/about/guidelines.xhtml

The first stage of screening will be based on the title and/or abstract of the articles. Articles that are clearly not relevant to the research question or clearly meet any of the exclusion criteria will be excluded. Articles, where there is doubt about relevancy, will be included to the next step. In the second stage of screening, the full text of the articles will be retrieved and assessed for eligibility. The reason for each article not being included will be documented and presented in a PRISMA flow diagram in the final review paper [23]. The screening/selection will be independently performed by two reviewers. A third reviewer will arbitrate any disagreement.

Data extraction

A data extraction form (Appendix 3) has been developed to extract data from included studies in a standardized and reproducible fashion. The form will be piloted and revised before being used in the review. If a study does not present needed data, authors of the study will be contacted. Extracted data will be presented in table form. The extraction will be independently conducted by two reviewers. A third reviewer will arbitrate any disagreement.

Data items

The following data items will be summarized from each study: author of publication; country of origin of study; publication year; type of study design; sample size of study; source from where study participants were recruited; definition and assessment of sibship and birth order; duration of follow-up; confounding factors adjusted included in studies; study outcomes and their assessment; analysis methods; and main results.

Quality assessment

Quality and risk of bias in the individual, included studies will be assessed using the Effective Public Health Practice Project Quality Assessment Tool (EPHPP) [24]. The EPHPP contains six domains of assessment for each study, including study design, selection bias, confounding, blinding, study collection, withdrawals and dropouts. Based on the grading of each of the six domains, a global quality grading will be derived for each study. Detailed results will be presented in a separate table in the final review paper. Appraisal of quality and

For peer review only

For peer review only - http://bmjopen.bmj.com/site/about/guidelines.xhtml

risk of bias will be independently performed by two reviewers. A third reviewer will arbitrate any disagreement.

Data synthesis

Descriptive tables will be generated to present the key characteristics of the included studies. The produced evidence will be synthesized narratively. In addition, studies that present numerically comparable and reasonably homogenous (in terms of clinical and epidemiological settings of study participants) data will be synthesized quantitatively with meta-analyses in RevMan 5, to produce pooled effect size estimates. Random-effects model will be applied in the meta-analyses, because the included studies, solely based on published literature, are anticipated to not be similar in every aspect and thus do not estimate the same effect. This model is more conservative and provides a realistic scenario in the context of studies gathered solely from published literature [25]. Separate meta-analyses will be undertaken for each of the factors investigate (sibship size and birth order) in relation to each asthma and allergy outcome. The results of the meta-analyses will be presented in forest plots.

Risk ratio (RR) will be used as the outcome measure in the meta-analyses, because of its intuitive interpretative feature [26]. Data from studies presenting effect measures as odds ratio (OR), incidence rate ratio (IRR), or hazard ratio (HR) will be converted to estimates of RR before combining with other studies, using the following formulas:

(a) RR ≈ IRR;

(b) RR ≈ HR or OR (if outcome is < 15% by the end of follow-up);

(c) RR ≈ $\sqrt{OR}$ or $\frac{1 - 0.5^{\sqrt{HR}}}{1 - 0.5^{\sqrt{\frac{1}{HR}}}}$ (if outcome is ≥ 15% by the end of follow-up) [27]

Calculation of I^2 will quantify heterogeneity between the included studies [28]. Consideration will be taken, regarding that this statistic can be biased in meta-analyses with few studies [29]. Subgroup analysis will be performed to explore potential reasons for heterogeneity between studies with the following subgroup variables: (a) study design; (b) quality appraisal of studies; (c) classification of the study country into “high-income”, “upper-middle-income”, “lower-middle-income”, and “low-income” economy, as defined by the World Bank [30]; (d) time during which the study was conducted, grouped into <1990, 1990-1999, 2000-2009, and 2010-2020; (e) participant age, grouped arbitrarily into < 1 year, 1-6 years, 7-

For peer review only - http://bmjopen.bmj.com/site/about/guidelines.xhtml

14 years, ≥ 15 years; (f) gender, grouped into "male" and "female". Subgroup analysis will be performed if there will be at least 4 (arbitrarily chosen cutoff [31]) studies in at least 2 subgroups. In addition, if more than 10 included studies present comparable numerical data [32], meta-regression will be performed to explore the impact of explanatory variables (covariates) on the observed heterogeneity in estimates across studies.

To investigate whether the conclusions of the review are independent of arbitrary decisions, sensitivity-analysis will be performed by only including studies which (a) reach either "strong" or "moderate" global rating of quality in accordance to EPHPP; (b) have objectively verified diagnosis of asthma or allergic disease as outcome, with either ICD codes or verified medical examination as the basis for diagnosis. The sensitivity analysis will be reported in a summary table.

Publication bias

Publication bias will be assessed with Funnel plot, as well as Begg's rank test and Egger's regression test [33, 34], with $p < 0.05$ being defined as statistically significant. In case of (significant) publication bias, the trim-and-fill method will be implemented to analyze its influence on the review results [35].

Patient and public involvement

No patients or participants were involved in the development of this protocol or the design of this study.

DISCUSSION

The conclusions that will potentially be drawn from this systematic review, will be limited by the quality of the included studies. For this research question, the fact that all included studies will be observational limits the establishment of causality between sibship size, birth order, and risk of asthma and allergic diseases [36].

A strength of this study is the comprehensiveness of the search strategy, including 15 of the leading databases of formally published literature, as well as gray literature. There will be no

For peer review only - http://bmjopen.bmj.com/site/about/guidelines.xhtml

restrictions in terms of language or geographical location. All these enable comprehensive identification of relevant studies for this research question. Furthermore, this systematic review will encompass a comprehensive spectrum of the most common asthma and allergic outcomes in relation to sibship size and birth order, thereby contributing to a broad overview of the existing evidence on the topic.

Asthma and allergic diseases pose a significant burden on both individuals and society. Whilst the role of sibship size and birth order in the development of these diseases have been investigated in several studies, albeit with conflicting evidence, a systematic review of existing studies is essential in providing a clearer appreciation of the underlying evidence. This protocol presents the methodology to perform a comprehensive systematic review and meta-analysis of existing literature on the topic.

ETHICS AND DISSEMINATION

Ethical approval will not be required due to the study being a systematic review of already published primary studies available in the public domain. Furthermore, patient consent will not be needed since data will stay aggregated [37, 38].

CONTRIBUTIONS

BIN conceived the study idea. DL wrote the protocol, with review and suggestions for improvement from BIN, EG, GW, and SSÖE. All authors (AI, BIN, DL, EG, GPM, GS, GW, SN, and SSÖE) commented and approved the last version being submitted.

FUNDING

There was no specific funding for this paper.

COMPETING INTERESTS

None declared.

For peer review only - http://bmjopen.bmj.com/site/about/guidelines.xhtml

REFERENCES

1. Dharmage SC, Perret JL, Custovic A. Epidemiology of Asthma in Children and Adults. Front Pediatr. 2019;7:246.

2. Nunes C, Pereira AM, Morais-Almeida M. Asthma costs and social impact. Asthma Res Pract. 2017;3:1.

3. Lundbäck B, Backman H, Lötvall J, Rönmark E. Is asthma prevalence still increasing? Expert Rev Respir Med. 2016;10(1):39-51.

4. Martinez FD. Trends in asthma prevalence, admission rates, and asthma deaths. Respir Care. 2008;53(5):561-565; discussion 565-567.

5. Hicke-Roberts A, Åberg N, Wennergren G, Hesselmar B. Allergic rhinoconjunctivitis continued to increase in Swedish children up to 2007, but asthma and eczema levelled off from 1991. Acta Paediatr. 2017;106(1):75-80.

6. Doll RJ, Joseph NI, McGarry D, Jhaveri D, Sher T, Hostoffer R. Epidemiology of Allergic Diseases. Allergy and Asthma: The Basics to Best Practices. Cham: Springer International Publishing; 2019. p. 31-51.

7. Pawankar R. Allergic diseases and asthma: a global public health concern and a call to action. World Allergy Organ J. 2014;7(1):12.

8. Bousquet J, Khaltaev N, Cruz AA, Denburg J, Fokkens WJ, Togias A, et al. Allergic Rhinitis and its Impact on Asthma (ARIA) 2008 update (in collaboration with the World Health Organization, GA(2)LEN and AllerGen). Allergy. 2008;63 Suppl 86:8-160.

9. Strachan DP. Hay fever, hygiene, and household size. BMJ (Clinical research ed). 1989;299(6710):1259-1260.

10. Pfefferle PI, Teich R, Renz H. The Immunological Basis of the Hygiene Hypothesis. Springer Japan. p. 325-348.

11. Stiemsma LT, Reynolds LA, Turvey SE, Finlay BB. The hygiene hypothesis: current perspectives and future therapies. Immunotargets Ther. 2015;4:143-157.

12. Lambrecht BN, Hammad H. The immunology of the allergy epidemic and the hygiene hypothesis. Nature Immunology. 2017;18(10):1076-1083.

13. Bloomfield SF, Rook GA, Scott EA, Shanahan F, Stanwell-Smith R, Turner P. Time to abandon the hygiene hypothesis: new perspectives on allergic disease, the human microbiome, infectious disease prevention and the role of targeted hygiene. Perspect Public Health. 2016;136(4):213-224.

14. Goldberg S, Israeli E, Schwartz S, Shochat T, Izbicki G, Toker-Maimon O, et al. Asthma prevalence, family size, and birth order. Chest. 2007;131(6):1747-1752.

15. Karmaus W. Does a higher number of siblings protect against the development of allergy and asthma? A review. Journal of Epidemiology & Community Health. 2002;56(3):209-217.

16. Moher D, Shamseer L, Clarke M, Ghersi D, Liberati A, Petticrew M, et al. Preferred reporting items for systematic review and meta-analysis protocols (PRISMA-P) 2015 statement. Systematic Reviews. 2015;4(1):1.

17. Zeller TA. Respiratory Conditions Update: Asthma. FP Essent. 2016;448:11-19.

18. Pinart M, Albang R, Maier D, Duran-Tauleria E, Mena G, Gimeno-Santos E, et al. Systematic Review on the Definition of Allergic Diseases in Children: The MeDALL Study. International Archives of Allergy and Immunology. 2015;168(2):110-121.

19. Banerji A. Hereditary angioedema: classification, pathogenesis, and diagnosis. Allergy Asthma Proc. 2011;32(6):403-407.

20. Khan KS, Kunz R, Kleijnen J, Antes G. Five steps to conducting a systematic review. J R Soc Med. 2003;96(3):118-121.

21. Haddaway NR, Collins AM, Coughlin D, Kirk S. The Role of Google Scholar in Evidence Reviews and Its Applicability to Grey Literature Searching. PLOS ONE. 2015;10(9):e0138237.

For peer review only - http://bmjopen.bmj.com/site/about/guidelines.xhtml

22. Gusenbauer M, Haddaway NR. Which academic search systems are suitable for systematic reviews or meta-analyses? Evaluating retrieval qualities of Google Scholar, PubMed, and 26 other resources. Research Synthesis Methods. 2020;11(2):181-217.
23. Liberati A, Altman DG, Tetzlaff J, Mulrow C, Gøtzsche PC, Ioannidis JP, et al. The PRISMA statement for reporting systematic reviews and meta-analyses of studies that evaluate healthcare interventions: explanation and elaboration. Bmj. 2009;339:b2700.
24. Armijo-Olivo S, Stiles CR, Hagen NA, Biondo PD, Cummings GG. Assessment of study quality for systematic reviews: a comparison of the Cochrane Collaboration Risk of Bias Tool and the Effective Public Health Practice Project Quality Assessment Tool: methodological research. J Eval Clin Pract. 2012;18(1):12-18.
25. Borenstein M, Hedges LV, Higgins JP, Rothstein HR. A basic introduction to fixed-effect and random-effects models for meta-analysis. Res Synth Methods. 2010;1(2):97-111.
26. Davies HT, Crombie IK, Tavakoli M. When can odds ratios mislead? Bmj. 1998;316(7136):989-991.
27. VanderWeele TJ, Ding P. Sensitivity Analysis in Observational Research: Introducing the E-Value. Ann Intern Med. 2017;167(4):268-274.
28. Higgins JP, Thompson SG, Deeks JJ, Altman DG. Measuring inconsistency in meta-analyses. Bmj. 2003;327(7414):557-560.
29. von Hippel PT. The heterogeneity statistic I(2) can be biased in small meta-analyses. BMC Med Res Methodol. 2015;15:35.
30. World Bank. World Bank country and lending groups. Washington (DC): World Bank; 2020 [cited 2020 Sept 6]. Available from: https://datahelpdesk.worldbank.org/knowledgebase/articles/906519.
31. Fu R, Gartlehner G, Grant M, Shamliyan T, Sedrakyan A, Wilt TJ, et al. AHRQ Methods for Effective Health Care Conducting Quantitative Synthesis When Comparing Medical Interventions: AHRQ and the Effective Health Care Program. Methods Guide for Effectiveness and Comparative Effectiveness Reviews. Rockville (MD): Agency for Healthcare Research and Quality (US); 2008.
32. Schmid CH, Stark PC, Berlin JA, Landais P, Lau J. Meta-regression detected associations between heterogeneous treatment effects and study-level, but not patient-level, factors. Journal of Clinical Epidemiology. 2004;57(7):683-697.
33. Egger M, Davey Smith G, Schneider M, Minder C. Bias in meta-analysis detected by a simple, graphical test. Bmj. 1997;315(7109):629-634.
34. Lin L, Chu H. Quantifying publication bias in meta-analysis. Biometrics. 2018;74(3):785-794.
35. Shi L, Lin L. The trim-and-fill method for publication bias: practical guidelines and recommendations based on a large database of meta-analyses. Medicine (Baltimore). 2019;98(23):e15987.
36. Petrisor B, Bhandari M. The hierarchy of evidence: Levels and grades of recommendation. Indian J Orthop. 2007;41(1):11-15.
37. Wormald R, Evans J. What Makes Systematic Reviews Systematic and Why are They the Highest Level of Evidence? Ophthalmic Epidemiology. 2018;25(1):27-30.
38. Suri H. Ethical Considerations of Conducting Systematic Reviews in Educational Research. In: Zawacki-Richter O, Kerres M, Bedenlier S, Bond M, Buntins K, editors. Systematic Reviews in Educational Research: Methodology, Perspectives and Application. Wiesbaden: Springer Fachmedien Wiesbaden; 2020. p. 41-54.

For peer review only

Appendix to

Sibship Size, Birth Order and Risk of Asthma and Allergy: Protocol for a Systematic Review and Meta-Analysis

Daniil Lisik, Athina Ioannidou, Gregorio Paolo Milani, Sungkutu Nyassi, Saliha Selin Özuygur Ermis, Giulia Spolidoro, Emma Goksör, Göran Wennergren, Bright I Nwaru

Appendix 1: PRISMA-P Checklist

PRISMA-P (Preferred Reporting Items for Systematic review and Meta-Analysis Protocols) 2015 checklist: recommended items to address in a systematic review protocol*

Section and topic	Item No	Checklist item	Reported on page
**ADMINISTRATIVE INFORMATION**			
Title:			
Identification	1a	Identify the report as a protocol of a systematic review	**1**
Update	1b	If the protocol is for an update of a previous systematic review, identify as such	**N/A**
Registration	2	If registered, provide the name of the registry (such as PROSPERO) and registration number	**2, 4 (PROSPERO, registration number CRD42020207905)**
Authors:			
Contact	3a	Provide name, institutional affiliation, e-mail address of all protocol authors; provide physical mailing address of corresponding author	**1**
Contributions	3b	Describe contributions of protocol authors and identify the guarantor of the review	**10**
Amendments	4	If the protocol represents an amendment of a previously completed or published protocol, identify as such and list changes; otherwise, state plan for documenting important protocol amendments	**N/A**
Support:			
Sources	5a	Indicate sources of financial or other support for the review	**10**
Sponsor	5b	Provide name for the review funder and/or sponsor	**N/A**
Role of sponsor or funder	5c	Describe roles of funder(s), sponsor(s), and/or institution(s), if any, in developing the protocol	**N/A**
**INTRODUCTION**			

For peer review only

Rationale	6	Describe the rationale for the review in the context of what is already known	**2-4**
Objectives	7	Provide an explicit statement of the question(s) the review will address with reference to participants, interventions, comparators, and outcomes (PICO)	**3-4**
**METHODS**			
Eligibility criteria	8	Specify the study characteristics (such as PICO, study design, setting, time frame) and report characteristics (such as years considered, language, publication status) to be used as criteria for eligibility for the review	**4-5**
Information sources	9	Describe all intended information sources (such as electronic databases, contact with study authors, trial registers or other grey literature sources) with planned dates of coverage	**6**
Search strategy	10	Present draft of search strategy to be used for at least one electronic database, including planned limits, such that it could be repeated	**5-6, Appendix 1**
Study records:			
Data management	11a	Describe the mechanism(s) that will be used to manage records and data throughout the review	**6**
Selection process	11b	State the process that will be used for selecting studies (such as two independent reviewers) through each phase of the review (that is, screening, eligibility and inclusion in meta-analysis)	**6-7**
Data collection process	11c	Describe planned method of extracting data from reports (such as piloting forms, done independently, in duplicate), any processes for obtaining and confirming data from investigators	**7, Appendix 2**
Data items	12	List and define all variables for which data will be sought (such as PICO items, funding sources), any pre-planned data assumptions and simplifications	**7**
Outcomes and prioritization	13	List and define all outcomes for which data will be sought, including prioritization of main and additional outcomes, with rationale	**5**
Risk of bias in individual studies	14	Describe anticipated methods for assessing risk of bias of individual studies, including whether this will be done at the outcome or study level, or both; state how this information will be used in data synthesis	**9**
Data synthesis	15a	Describe criteria under which study data will be quantitatively synthesised	**8**
	15b	If data are appropriate for quantitative synthesis, describe planned summary measures, methods of handling data and methods of combining data from studies, including any planned exploration of consistency (such as I^2, Kendall's τ)	**8**
	15c	Describe any proposed additional analyses (such as sensitivity or subgroup analyses, meta-regression)	**8-9**
	15d	If quantitative synthesis is not appropriate, describe the type of summary planned	**8**

For peer review only

Meta-bias(es)	16	Specify any planned assessment of meta-bias(es) (such as publication bias across studies, selective reporting within studies)	**9**
Confidence in cumulative evidence	17	Describe how the strength of the body of evidence will be assessed (such as GRADE)	**9**

*** It is strongly recommended that this checklist be read in conjunction with the PRISMA-P Explanation and Elaboration (cite when available) for important clarification on the items. Amendments to a review protocol should be tracked and dated. The copyright for PRISMA-P (including checklist) is held by the PRISMA-P Group and is distributed under a Creative Commons Attribution Licence 4.0.**

From: Shamseer L, Moher D, Clarke M, Ghersi D, Liberati A, Petticrew M, Shekelle P, Stewart L, PRISMA-P Group. Preferred reporting items for systematic review and meta-analysis protocols (PRISMA-P) 2015: elaboration and explanation. BMJ. 2015 Jan 2;349(jan02 1):g7647.

For peer review only

Appendix to

Sibship Size, Birth Order and Risk of Asthma and Allergy: Protocol for a Systematic Review and Meta-Analysis

Daniil Lisik, Athina Ioannidou, Gregorio Paolo Milani, Sungkutu Nyassi, Saliha Selin Özuygur Ermis, Giulia Spolidoro, Emma Goksör, Göran Wennergren, Bright I Nwaru

Appendix 2: Search Strategies

For peer review only

Colorization

Red: controlled vocabulary/thesaurus

Blue: free-text

Green: referral to search query component (table row; #)

AMED

#	Search term(s)
1	(birth order* or birth rank* or multiple birth* or parity).mp.
2	exp Family Characteristics/ or (family characteristic* or family size* or family structure* or family demograph* or family composition or household size* or household demograph* or household composition).mp.
3	exp Sibling Relations/ or (sibling* or sister* or brother* or sibship size* or sibship*).mp.
4	or/1-3
5	exp Asthma/ or (bronchial asthma* or exercise-induced asthma* or exercise-induced bronchospasm* or asthma* or respiratory hypersensitivit* or airway hyper responsiveness or airway hyper-responsiveness or respiratory hyper responsiveness or respiratory hyper-responsiveness or wheez*).mp.
6	exp Hypersensitivity/ or exp Hypersensitivity Immediate/ or exp Hypersensitivity Delayed/ or (immediate hypersensitivit* or delayed hypersensitivit* or IgE-mediated hypersensitivit* or type I hypersensitivit* or type IV hypersensitivit* or atopic sensitization or atop* or allergic sensitization or allerg*).mp.
7	exp Dermatitis/ or exp Anaphylaxis/ or (atopic dermatitis or dermatitis or neurodermatiti* or besniers prurigo or besnier prurigo or atopic eczema or eczema or urticari* or anaphyla* or quinckes edema or quincke edema or angioneurotic edema or angioedema or hives).mp.
8	exp Food Hypersensitivity/ or (food hypersensitivit* or food allerg* or egg hypersensitivit* or egg allerg* or milk hypersensitivit* or milk allerg* or shellfish hypersensitivit* or shellfish allerg* or wheat hypersensitivit* or wheat allerg* or nut hypersensitivit* or nut allerg* or peanut hypersensitivit* or peanut allerg* or groundnut hypersensitivit* or groundnut allerg*).mp.

9	exp Rhinitis/ or exp Conjunctivitis/ or (allergic rhinoconjunctiviti* or rhinoconjunctiviti* or allergic rhiniti* or seasonal allergic rhiniti* or perennial allergic rhiniti* or rhiniti* or allergic conjunctiviti* or vernal keratoconjunctiviti* or vernal conjunctiviti* or giant papillary conjunctiviti* or hay fever or hayfever or pollinosis or nasal catarrh*).mp.
10	or/5-9
11	4 and 10
Full query<br>((birth order* or birth rank* or multiple birth* or parity).mp. or exp Family Characteristics/ or (family characteristic* or family size* or family structure* or family demograph* or family composition or household size* or household demograph* or household composition).mp. or exp Sibling Relations/ or (sibling* or sister* or brother* or sibship size* or sibship*).mp.) and (exp Asthma/ or (bronchial asthma* or exercise-induced asthma* or exercise-induced bronchospasm* or asthma* or respiratory hypersensitivit* or airway hyper responsiveness or airway hyper-responsiveness or respiratory hyper responsiveness or respiratory hyper-responsiveness or wheez*).mp. or exp Hypersensitivity/ or exp Hypersensitivity Immediate/ or exp Hypersensitivity Delayed/ or (immediate hypersensitivit* or delayed hypersensitivit* or IgE-mediated hypersensitivit* or type I hypersensitivit* or type IV hypersensitivit* or atopic sensitization or atop* or allergic sensitization or allerg*).mp. or exp Dermatitis/ or exp Anaphylaxis/ or (atopic dermatitis or dermatitis or neurodermatiti* or besniers prurigo or besnier prurigo or atopic eczema or eczema or urticari* or anaphyla* or quinckes edema or quincke edema or angioneurotic edema or angioedema or hives).mp. or exp Food Hypersensitivity/ or (food hypersensitivit* or food allerg* or egg hypersensitivit* or egg allerg* or milk hypersensitivit* or milk allerg* or shellfish hypersensitivit* or shellfish allerg* or wheat hypersensitivit* or wheat allerg* or nut hypersensitivit* or nut allerg* or peanut hypersensitivit* or peanut allerg* or groundnut hypersensitivit* or groundnut allerg*).mp. or exp Rhinitis/ or exp Conjunctivitis/ or (allergic rhinoconjunctiviti* or rhinoconjunctiviti* or allergic rhiniti* or seasonal allergic rhiniti* or perennial allergic rhiniti* or rhiniti* or allergic conjunctiviti* or vernal keratoconjunctiviti* or vernal conjunctiviti* or giant papillary conjunctiviti* or hay fever or hayfever or pollinosis or nasal catarrh*).mp.)	

exp = include all narrower subject headings; mp= abstract, heading words, title

CABI; OAIster; Open Access Theses and Dissertations; Open Grey; ProQuest Dissertations & Theses Global; SciELO; WHO Global Index Medicus

#	Search term(s)
1	"birth order" OR "multiple births" OR "birth rank" OR "parity"
2	"family characteristics" OR "family size" OR "family structure" OR "family demography" OR "family composition" OR "household size" OR "household demography" OR "household composition"
3	"siblings" OR "sibling relations" OR "sister" OR "brother" OR "sibship"
4	"exercise-induced bronchospasm" OR "asthma" OR "airway hyper-responsiveness" OR "respiratory hyper-responsiveness" OR "wheeze" OR "wheezing"
5	”hypersensitivity" OR "atopic sensitization" OR "atopy" OR "allergic sensitization" OR "allergic disease" OR "allergic condition" OR "allergy" OR "allergies"

For peer review only - http://bmjopen.bmj.com/site/about/guidelines.xhtml

6	"dermatitis" OR "eczema" OR "neurodermatitis" OR "besnier's prurigo" OR "urticaria" OR "anaphylaxis" OR "anaphylactic shock" OR "quincke's edema" OR "angionuerotic edema" OR "angioedema" OR "hives"
8	"rhinoconjunctivitis" OR "rhinitis" OR "allergic conjunctivitis" OR "vernal keratoconjunctivitis" OR "vernal conjunctivitis" OR "giant papillary conjunctivitis" OR "hay fever" OR "pollinosis" OR "pollenosis" OR "nasal catarrh"
9	1 OR 2 OR 3
10	4 OR 5 OR 6 OR 7 OR 8
11	9 AND 10

Full query

("birth order" OR "multiple births" OR "birth rank" OR "parity" OR "family characteristics" OR "family size" OR "family structure" OR "family demography" OR "family composition" OR "household size" OR "household demography" OR "household composition" OR "siblings" OR "sibling relations" OR "sister" OR "brother" OR "sibship") AND ("exercise-induced bronchospasm" OR "asthma" OR "airway hyper-responsiveness" OR "respiratory hyper-responsiveness" OR "wheeze" OR "wheezing" OR "hypersensitivity" OR "atopic sensitization" OR "atopy" OR "allergic sensitization" OR "allergic disease" OR "allergic condition" OR "allergy" OR "allergies" OR "dermatitis" OR "eczema" OR "neurodermatitis" OR "besnier's prurigo" OR "urticaria" OR "anaphylaxis" OR "anaphylactic shock" OR "quincke's edema" OR "angionuerotic edema" OR "angioedema" OR "hives" OR "rhinoconjunctivitis" OR "rhinitis" OR "allergic conjunctivitis" OR "vernal keratoconjunctivitis" OR "vernal conjunctivitis" OR "giant papillary conjunctivitis" OR "hay fever" OR "pollinosis" OR "pollenosis" OR "nasal catarrh")

CINAHL

#	Search term(s)
1	(MH 'Birth Order+') OR (MH 'Parity+') OR 'birth order*' OR 'birth rank*' OR 'multiple birth*' OR 'parity'
2	(MH 'Family Characteristics+') OR (MH 'Family Health+') OR 'family charactersitic*' OR 'family size*' OR 'family structure*' OR 'family demograph*' OR 'family composition' OR 'household size*' OR 'household demograph*' OR 'household composition'
3	(MH 'Siblings+') OR (MH 'Sibling Relations+') OR 'sibling*' OR 'sister*' OR 'brother*' OR 'sibship size*' OR 'sibship*'
4	(MH 'Asthma+') OR (MH 'Respiratory Hypersensitivity+') OR 'bronchial asthma*' OR 'exercise-induced asthma' OR 'asthma*' OR 'exercise-induced bronchospasm' OR 'respiratory hypersensitivit*' OR 'airway hyper responsiveness' OR 'airway hyper-responsiveness' OR 'respiratory hyper responsiveness' OR 'respiratory hyper-responsiveness' OR 'wheez*'
5	(MH 'Hypersensitivity+') OR (MH 'Hypersensitivity, Immediate+') OR (MH 'Hypersensitivity, Delayed+') OR (MH 'Allergy and Immunology+') OR 'immedate hypersensitivit*' OR 'delayed hypersensitivit*' OR 'IgE-mediated hypersensitivit*' OR 'type I hypersensitivit*' OR 'type IV hypersensitivit*' OR 'hypersensitivit*' OR 'atopic sensitiziation' OR 'atop*' OR 'allergic sensitization' OR 'allergic disease* OR 'allerg*'

For peer review only

6	(MH ’Dermatitis, Atopic+’) OR (MH ’Eczema+’) OR (MH ‘Angioedema+’) OR (MH ‘Anaphylaxis+’) OR (MH ’Urticaria+’) OR ’atopic dermatitis’ OR ’dermatitis’ OR ’atopic eczema’ OR ’eczema’ OR ’nerudoarmatiti*’ OR “besnier’s prurigo” OR ‘besniers prurigo’ OR ‘besnier prurigo’ OR ’urticari*’ OR ’hives’ OR ’anaphyla*’ OR ”quincke’s edema” OR ’quinckes edema’ OR ’quincke edema’ OR ’angioneurotic edema’ OR ’angioedema’
7	(MH ’Food Hypersensitivity+’) OR ’food hypersensitivit*’ OR ’food allerg*’ OR ’egg hypersensitivit*’ OR ’egg allerg*’ OR ’milk hypersensitivit*’ OR ’milk allerg*’ OR ’shellfish hypersensitivit*’ OR ’shellfish allerg*’ OR ’wheat hypersensitivit*’ OR ’wheat allerg*’ OR ’nut hypersensitivit*’ OR ’nut allerg*’ OR ’peanut hypersensitivit*’ OR ’peanut allerg*’ OR ’groundnut hypersensitivit*’ OR ’groundnut allerg*’
8	(MH ’Rhinitis, Allergic+’) OR (MH ’Rhinitis, Allergic, Seasonal+’) OR (MH ’Rhinitis, Allergic, Perennial+’) OR (MH ’Rhinitis+’) OR (MH ’Conjunctivitis, Allergic+’) OR (MH ’Conjunctivitis+’) OR ’allergic rhinoconjunctiviti*’ OR ’rhinoconjunctiviti*’ OR ’allergic rhiniti*’ OR ’seasonal allergic rhiniti*’ OR ’perennial allergic rhiniti*’ OR ’rhiniti*’ OR ’allergic conjunctiviti*’ OR ’vernal keratoconjunctiviti*’ OR ’vernal conjunctiviti*’ OR ’giant papillary conjunctiviti*’ OR ’hay fever’ OR ’hayfever’ OR ’pollinosis’ OR ’pollenosis’ OR ’nasal catarrh’
9	1 OR 2 OR 3
10	4 OR 5 OR 6 OR 7 OR 8
11	9 AND 10

Full query

((MH "Birth Order+") OR (MH "Parity+") OR "birth order*" OR "birth rank*" OR "multiple birth*" OR "parity" OR (MH "Family Characteristics+") OR (MH "Family Health+") OR "family charactersitic*" OR "family size*" OR "family structure*" OR "family demograph*" OR "family composition" OR "household size*" OR "household demograph*" OR "household composition" OR (MH "Siblings+") OR (MH "Sibling Relations+") OR "sibling*" OR "sister*" OR "brother*" OR "sibship size*" OR "sibship*") AND ((MH "Asthma+") OR (MH "Respiratory Hypersensitivity+") OR "bronchial asthma*" OR "exercise-induced asthma" OR "asthma*" OR "exercise-induced bronchospasm" OR "respiratory hypersensitivit*" OR ‘airway hyper responsiveness" OR "airway hyper-responsiveness" OR "respiratory hyper responsiveness" OR "respiratory hyper-responsiveness" OR "wheez*" OR (MH "Hypersensitivity+") OR (MH "Hypersensitivity, Immediate+") OR (MH "Hypersensitivity, Delayed+") OR (MH "Allergy and Immunology+") OR "immedate hypersensitivit*" OR "delayed hypersensitivit*" OR "IgE-mediated hypersensitivit*" OR "type I hypersensitivit*" OR "type IV hypersensitivit*" OR "hypersensitivit*" OR "atopic sensitiziation" OR "atop*" OR "allergic sensitization" OR "allergic disease* OR "allerg*" OR (MH "Dermatitis, Atopic+") OR (MH "Eczema+") OR (MH ‘Angioedema+") OR (MH ‘Anaphylaxis+") OR (MH "Urticaria+") OR "atopic dermatitis" OR "dermatitis" OR "atopic eczema" OR "eczema" OR "nerudoarmatiti*" OR "besnier's prurigo" OR "besniers prurigo" OR "besnier prurigo" OR "urticari*" OR "hives" OR "anaphyla*" OR "quincke's edema" OR "quinckes edema" OR "quincke edema" OR "angioneurotic edema" OR "angioedema" OR (MH "Food Hypersensitivity+") OR "food hypersensitivit*" OR "food allerg*" OR "egg hypersensitivit*" OR "egg allerg*" OR "milk hypersensitivit*" OR "milk allerg*" OR "shellfish hypersensitivit*" OR "shellfish allerg*" OR "wheat hypersensitivit*" OR "wheat allerg*" OR "nut hypersensitivit*" OR "nut allerg*" OR "peanut hypersensitivit*" OR "peanut allerg*" OR "groundnut hypersensitivit*" OR "groundnut allerg*" OR (MH "Rhinitis, Allergic+") OR (MH "Rhinitis, Allergic, Seasonal+") OR (MH "Rhinitis, Allergic, Perennial+") OR (MH "Rhinitis+") OR (MH "Conjunctivitis, Allergic+") OR (MH "Conjunctivitis+") OR "allergic rhinoconjunctiviti*" OR "rhinoconjunctiviti*" OR

For peer review only

"allergic rhiniti*" OR "seasonal allergic rhiniti*" OR "perennial allergic rhiniti*" OR "rhiniti*" OR "allergic conjunctiviti*" OR "vernal keratoconjunctiviti*" OR "vernal conjunctiviti*" OR "giant papillary conjunctiviti*" OR "hay fever" OR "hayfever" OR "pollinosis" OR "pollenosis" OR "nasal catarrh"))

MH = subject heading

Embase

#	Search term(s)
1	sibship.mp.
2	birth order.mp. or exp birth order/
3	birth rank.mp.
4	multiple birth.mp.
5	parity.mp. or exp parity/
6	family characteristic.mp. or exp family size/
7	family structure.mp.
8	family demograph.mp.
9	family demograph*.mp.
10	family composition.mp.
11	household size.mp.
12	household demograph*.mp.
13	household composition.mp.
14	exp sibling relation/ or sibling.mp. or sibling/
15	exp sister/ or sister.mp.
16	siblings.mp.
17	sisters.mp.
18	brother.mp. or exp brother/
19	brothers.mp.
20	or/1-19
21	exp Asthma/ or (bronchial asthma* or exercise-induced asthma* or exercise-induced bronchospasm* or asthma* or respiratory hypersensitivit* or airway hyper responsiveness or airway hyper-responsiveness or respiratory hyper responsiveness or respiratory hyper-responsiveness or wheez*).mp.
22	exp Hypersensitivity/ or exp Hypersensitivity Immediate/ or exp Hypersensitivity Delayed/ or (immediate hypersensitivit* or delayed hypersensitivit* or IgE-mediated hypersensitivit* or type I hypersensitivit* or type IV hypersensitivit* or atopic sensitization or atop* or allergic sensitization or allerg*).mp.
23	exp Dermatitis/ or exp Anaphylaxis/ or (atopic dermatitis or dermatitis or neurodermatiti* or besnier prurigo or besniers prurigo or besnier prurigo or atopic eczema or eczema or urticari* or anaphyla* or quincke edema or quinckes edema or quincke edema or angioneurotic edema or angioedema or hives).mp.

For peer review only

24	exp Food Hypersensitivity/ or (food hypersensitivit* or food allerg* or egg hypersensitivit* or egg allerg* or milk hypersensitivit* or milk allerg* or shellfish hypersensitivit* or shellfish allerg* or wheat hypersensitivit* or wheat allerg* or nut hypersensitivit* or nut allerg* or peanut hypersensitivit* or peanut allerg* or groundnut hypersensitivit* or groundnut allerg*).mp.
25	exp Rhinitis/ or exp Conjunctivitis/ or (allergic rhinoconjunctiviti* or rhinoconjunctiviti* or allergi rhiniti* or seasonal allergic rhiniti* or perennial allergic rhiniti* or rhiniti* or allergic conjunctiviti* or vernal keratoconjunctiviti* or vernal conjunctiviti* or giant papillary conjunctiviti* or hay fever or hayfever or pollinosis or pollinosis or nasal catarrh*).mp.
26	or/21-25
27	20 and 26

Google Scholar

#	**Search term(s)**
1	"family size" OR "family structure" OR "household size"
2	"sibling" OR "sibship"
3	"asthma" OR "wheezing"
4	"atopy" OR "allergy"
5	"eczema" OR "urticaria" OR "angioedema" OR "anaphylaxis"
6	"rhinitis" OR "allergic conjunctivitis" OR "hay fever"
7	1 OR 2
8	3 OR 4 OR 5 OR 6
9	7 AND 8
Full query<br>("family size" OR "family structure" OR "household size" OR "sibling" OR "sibship") AND ("asthma" OR "wheezing" OR "atopy" OR "allergy" OR "anaphylaxis" OR "eczema" OR "urticaria" OR "angioedema" OR "rhinitis" OR "allergic conjunctivitis" OR "hay fever")	

PsycINFO

#	**Search term(s)**
1	SU.EXACT.EXPLODE("Birth Order") OR TI,AB(”birth order*”) OR TI,AB(”multiple birth*”) OR TI,AB(”birth rank*”) OR TI,AB(”parity”)
2	SU.EXACT.EXPLODE("Family Structure") OR SU.EXACT.EXPLODE("Family Size") OR TI,AB(”family characteristic*”) OR TI,AB(”family size*”) OR TI,AB(”family structure*”) OR TI,AB(”family demograph*”) OR TI,AB(”family composition”) OR TI,AB(”household size*”) OR TI,AB(”household demograph*”) OR TI,AB(”household composition”)
3	SU.EXACT.EXPLODE("Siblings") OR SU.EXACT.EXPLODE("Sibling Relations") OR TI,AB(”sibling*”’) OR TI,AB(”sister*”) OR TI,AB(”brother*”) OR TI,AB(”sibship size*”) OR TI,AB(”sibship size*”) OR TI,AB(”sibship*”)

4	SU.EXACT.EXPLODE("Asthma") OR TI,AB(”bronchial asthma*”) OR TI,AB(”exercise-induced asthma*”) OR TI,AB(”asthma*”) OR TI,AB(”exercise-induced bronchospasm*”) OR TI,AB(”respiratory hypersensitivit*”) OR TI,AB(”airway hyper responsiveness”) OR TI,AB(”airway hyper-responsiveness”) OR TI,AB(”respiratory hyper responsiveness”) OR TI,AB(”respiratory hyper-responsiveness”) OR TI,AB(”wheez*”)
5	SU.EXACT.EXPLODE("Allergic Disorders") OR TI,AB(”immediate hypersensitivit*”) OR TI,AB(”delayed hypersensitivit*”) OR TI,AB(”hypersensitivit*”) OR TI,AB(”IgE-mediated hypersensitivit*”) OR TI,AB(”type I hypersensitivit*”) OR TI,AB(”type IV hypersensitivit*”) OR TI,AB(”atopic sensitization”) OR TI,AB(”atop*”) OR TI,AB(”allergic sensitization”) OR TI,AB(”allergic disease*”) OR TI,AB(”allerg*”)
6	SU.EXACT.EXPLODE("Allergic Skin Disorders") OR SU.EXACT.EXPLODE("Neurodermatitis") OR SU.EXACT.EXPLODE("Dermatitis") OR SU.EXACT.EXPLODE("Eczema") OR SU.EXACT.EXPLODE("Anaphylactic Shock") OR TI,AB(”atopic dermatitis”) OR TI,AB(”dermatitis”) OR TI,AB(”atopic eczema”) OR TI,AB(”eczema”) OR TI,AB(”neurodermatiti*”) OR TI,AB(”besnier’s prurigo”) OR TI,AB(”besniers prurigo”) OR TI,AB(”besnier prurigo”) OR TI,AB(”urticari*”) OR TI,AB(”hives”) OR TI,AB(”anaphyla*”) OR TI,AB(”quincke’s edema”) OR TI,AB(”quinckes edema”) OR TI,AB(”quincke edema”) OR TI,AB(”angioneurotic edema”) OR TI,AB(”angioedema”)
7	SU.EXACT.EXPLODE("Food Allergies") OR TI,AB(”food hypersensitivit*”) OR TI,AB(”food allerg*”) OR TI,AB(”egg hypersensitivit*”) OR TI,AB(”egg allerg*”) OR TI,AB(”milk hypersensitivit*”) OR TI,AB(”milk allerg*”) OR TI,AB(”shellfish hypersensitivit*”) OR TI,AB(”shellfish allerg*”) OR TI,AB(”wheat hypersensitivit*”) OR TI,AB(”wheat allerg*”) OR TI,AB(”nut hypersensitivit*”) OR TI,AB(”nut allerg*”) OR TI,AB(”peanut hypersensitivit*”) OR TI,AB(”peanut allerg*”) OR TI,AB(”groundnut hypersensitivit*”) OR TI,AB(”groundnut allerg*”)
8	TI,AB(”allergic rhinoconjunctiviti*”) OR TI,AB(”rhinoconjunctiviti*”) OR TI,AB(”allergic rhiniti*”) OR TI,AB(”rhiniti*”) OR TI,AB(”seasonal allergic rhiniti*”) OR TI,AB(”perennial allergic rhiniti*”) OR TI,AB(”allergic conjunctiviti*”) OR TI,AB(”vernal keratoconjunctiviti*”) OR TI,AB(”vernal conjunctiviti*”) OR TI,AB(”giant papillary conjunctiviti*”) OR TI,AB(”hay fever”) OR TI,AB(”hayfever”) OR TI,AB(“pollinosis”) OR TI,AB(”pollenosis”) OR TI,AB(”nasal catarrh*”)
9	1 OR 2 OR 3
10	4 OR 5 OR 6 OR 7 OR 8
11	9 AND 10

Full query

(SU.EXACT.EXPLODE("Birth Order") OR TI,AB("birth order*") OR TI,AB("multiple birth*") OR TI,AB("birth rank*") OR TI,AB("parity") OR SU.EXACT.EXPLODE("Family Structure") OR SU.EXACT.EXPLODE("Family Size") OR TI,AB("family characteristic*") OR TI,AB("family size*") OR TI,AB("family structure*") OR TI,AB("family demograph*") OR TI,AB("family composition") OR TI,AB("household size*") OR TI,AB("household demograph*") OR TI,AB("household composition") OR SU.EXACT.EXPLODE("Siblings") OR SU.EXACT.EXPLODE("Sibling Relations") OR TI,AB("sibling*") OR TI,AB("sister*") OR TI,AB("brother*") OR

TI,AB("sibship size*") OR TI,AB("sibship size*") OR TI,AB("sibship*")) AND (SU.EXACT.EXPLODE("Asthma") OR TI,AB("bronchial asthma*") OR TI,AB("exercise-induced asthma*") OR TI,AB("asthma*") OR TI,AB("exercise-induced bronchospasm*") OR TI,AB("respiratory hypersensitivit*") OR TI,AB("airway hyper responsiveness") OR TI,AB("airway hyper-responsiveness") OR TI,AB("respiratory hyper responsiveness") OR TI,AB("respiratory hyper-responsiveness") OR TI,AB("wheez*") OR SU.EXACT.EXPLODE("Allergic Disorders") OR TI,AB("immediate hypersensitivit*") OR TI,AB("delayed hypersensitivit*") OR TI,AB("hypersensitivit*") OR TI,AB("IgE-mediated hypersensitivit*") OR TI,AB("type I hypersensitivit*") OR TI,AB("type IV hypersensitivit") OR TI,AB("atopic sensitization") OR TI,AB("atop*") OR TI,AB("allergic sensitization") OR TI,AB("allergic disease*") OR TI,AB("allerg*") OR SU.EXACT.EXPLODE("Allergic Skin Disorders") OR SU.EXACT.EXPLODE("Neurodermatitis") OR SU.EXACT.EXPLODE("Dermatitis") OR SU.EXACT.EXPLODE("Eczema") OR SU.EXACT.EXPLODE("Anaphylactic Shock") OR TI,AB("atopic dermatitis") OR TI,AB("dermatitis") OR TI,AB("atopic eczema") OR TI,AB("eczema") OR TI,AB("neurodermatiti*") OR TI,AB("besnier's prurigo") OR TI,AB("besniers prurigo") OR TI,AB("besnier prurigo") OR TI,AB("urticari*") OR TI,AB("hives") OR TI,AB("anaphyla*") OR TI,AB("quincke's edema") OR TI,AB("quinckes edema") OR TI,AB("quincke edema") OR TI,AB("angioneurotic edema") OR TI,AB("angioedema") OR SU.EXACT.EXPLODE("Food Allergies") OR TI,AB("food hypersensitivit*") OR TI,AB("food allerg*") OR TI,AB("egg hypersensitivit*") OR TI,AB("egg allerg*") OR TI,AB("milk hypersensitivit*") OR TI,AB("milk allerg*") OR TI,AB("shellfish hypersensitivit*") OR TI,AB("shellfish allerg*") OR TI,AB("wheat hypersensitivit*") OR TI,AB("wheat allerg*") OR TI,AB("nut hypersensitivit*") OR TI,AB("nut allerg*") OR TI,AB("peanut hypersensitivit*") OR TI,AB("peanut allerg*") OR TI,AB("groundnut hypersensitivit*") OR TI,AB("groundnut allerg*") OR TI,AB("allergic rhinoconjunctiviti*") OR TI,AB("rhinoconjunctiviti*") OR TI,AB("allergic rhiniti*") OR TI,AB("rhiniti*") OR TI,AB("seasonal allergic rhiniti*") OR TI,AB("perennial allergic rhiniti*") OR TI,AB("allergic conjunctiviti*") OR TI,AB("vernal keratoconjunctiviti*") OR TI,AB("vernal conjunctiviti*") OR TI,AB("giant papillary conjunctiviti*") OR TI,AB("hay fever") OR TI,AB("hayfever") OR TI,AB("pollinosis") OR TI,AB("pollenosis") OR TI,AB("nasal catarrh*"))

SU = all subjects and indexing; TI,AB = title, abstract

PubMed

#	**Search term(s)**
1	Birth Order[mh] OR Parity[mh] OR birth order*[tiab] OR multiple birth*[tiab] OR birth rank*[tiab] OR parity[tiab]
2	Family Characteristics[mh] OR Family Health[mh] OR family characteristic*[tiab] OR family size*[tiab] OR family structure*[tiab] OR family demograph*[tiab] OR family composition[tiab] OR household size*[tiab] OR household demograph*[tiab] OR household composition[tiab]
3	Siblings[mh] OR Sibling Relations[mh] OR sibling*[tiab] OR sister*[tiab] OR brother*[tiab] OR sibship size*[tiab] OR sibship*[tiab]
4	Asthma[mh] OR Asthma, Exercise-Induced[mh] OR Respiratory Hypersensitivity[mh] OR bronchial asthma*[tiab] OR exercise-induced asthma*[tiab] OR exercise-induced bronchospasm*[tiab] OR asthma*[tiab] OR respiratory hypersensitivit*[tiab] OR airway hyper responsiveness[tiab] OR airway hyper-responsiveness[tiab] OR respiratory hyper responsiveness[tiab] OR respiratory hyper-responsiveness[tiab] OR wheez*[tiab]

5	Hypersensitivity[mh] OR Hypersensitivity, Immediate[mh] OR Hypersensitivity, Delayed[mh] OR Allergy and Immunology[mh] OR Allergens / Immunology[mh] OR immediate hypersensitivit*[tiab] OR delayed hypersensitivit*[tiab] OR hypersensitivit*[tiab] OR IgE-mediated hypersensitivit*[tiab] OR type I hypersensitivit*[tiab] OR type IV hypersensitivit*[tiab] OR atopic sensitization[tiab] OR atop*[tiab] OR allergic sensitization[tiab] OR allergic disease*[tiab] OR allerg*[tiab]
6	Dermatitis, Atopic[mh] OR Eczema[mh] OR Angioedema[mh] OR Anaphylaxis[mh] OR Urticaria[mh] OR atopic dermatitis[tiab] OR dermatitis[tiab] OR atopic eczema[tiab] OR eczema[tiab] OR neurodermatiti*[tiab] OR besnier's prurigo[tiab] OR besniers prurigo[tiab] OR besnier prurigo[tiab] OR urticari*[tiab] OR anaphyla*[tiab] OR quincke edema[tiab] OR quinckes edema[tiab] OR quincke's edema[tiab] OR angioneurotic edema[tiab] OR angioedema[tiab] OR hives[tiab]
7	Food Hypersensitivity[mh] OR food hypersensitivit*[tiab] OR food allerg*[tiab] OR egg allerg*[tiab] OR egg hypersensitivit*[tiab] OR milk allerg*[tiab] OR milk hypersensitivit*[tiab] OR shellfish allerg*[tiab] OR shellfish hypersensitivit*[tiab] OR wheat allerg*[tiab] OR wheat hypersensitivit*[tiab] OR nut allerg*[tiab] OR nut hypersensitivit*[tiab] OR peanut allerg*[tiab] OR peanut hypersensitivit*[tiab] OR groundnut hypersensitivit*[tiab]
8	Pollen / Immunology[mh] OR Rhinitis, Allergic[mh] OR Rhinitis, Allergic, Seasonal[mh] OR Rhinitis, Allergic, Perennial[mh] OR Rhinitis[mh] OR Conjunctivitis, Allergic[mh] OR Conjunctivitis / Immunology[mh] OR Conjunctivitis / Epidemiology[mh] OR Conjunctivitis / Etiology[mh] OR allergic rhinoconjunctiviti*[tiab] OR rhinoconjunctiviti*[tiab] OR allergic rhiniti*[tiab] OR rhiniti*[tiab] OR seasonal allergic rhiniti*[tiab] OR perennial allergic rhiniti*[tiab] OR allergic conjunctiviti*[tiab] OR vernal keratoconjunctiviti*[tiab] OR vernal conjunctiviti*[tiab] OR giant papillary conjunctiviti*[tiab] OR hay fever[tiab] OR hayfever[tiab] OR pollinosis[tiab] OR pollenosis[tiab] OR nasal catarrh*[tiab]
9	1 OR 2 OR 3
10	4 OR 5 OR 6 OR 7 OR 8
11	9 AND 10

Full query

(Birth Order[mh] OR Parity[mh] OR birth order*[tiab] OR multiple birth*[tiab] OR birth rank*[tiab] OR parity[tiab] OR Family Characteristics[mh] OR Family Health[mh] OR family characteristic*[tiab] OR family size*[tiab] OR family structure*[tiab] OR family demograph*[tiab] OR family composition[tiab] OR household size*[tiab] OR household demograph*[tiab] OR household composition[tiab] OR Siblings[mh] OR Sibling Relations[mh] OR sibling*[tiab] OR sister*[tiab] OR brother*[tiab] OR sibship size*[tiab] OR sibship*[tiab]) AND (Asthma[mh] OR Asthma, Exercise-Induced[mh] OR Respiratory Hypersensitivity[mh] OR bronchial asthma*[tiab] OR exercise-induced asthma*[tiab] OR exercise-induced bronchospasm*[tiab] OR asthma*[tiab] OR respiratory hypersensitivit*[tiab] OR airway hyper responsiveness[tiab] OR airway hyper-responsiveness[tiab] OR respiratory hyper responsiveness[tiab] OR respiratory hyper-responsiveness[tiab] OR wheez*[tiab] OR Hypersensitivity[mh] OR Hypersensitivity, Immediate[mh] OR Hypersensitivity, Delayed[mh] OR Allergy and Immunology[mh] OR Allergens / Immunology[mh] OR immediate hypersensitivit*[tiab] OR delayed hypersensitivit*[tiab] OR hypersensitivit*[tiab] OR IgE-mediated hypersensitivit*[tiab] OR type I hypersensitivit*[tiab] OR type IV hypersensitivit*[tiab] OR atopic sensitization[tiab] OR atop*[tiab] OR allergic sensitization[tiab] OR allergic disease*[tiab] OR allerg*[tiab] OR Dermatitis, Atopic[mh] OR Eczema[mh] OR Angioedema[mh] OR Anaphylaxis[mh] OR Urticaria[mh] OR atopic dermatitis[tiab] OR dermatitis[tiab] OR atopic eczema[tiab] OR eczema[tiab] OR neurodermatiti*[tiab]OR besnier's prurigo[tiab] OR besniers prurigo[tiab] OR besnier prurigo[tiab] OR urticari*[tiab] OR anaphyla*[tiab] OR quincke edema[tiab] OR quinckes edema[tiab] OR quincke's edema[tiab] OR angioneurotic edema[tiab] OR angioedema[tiab] OR hives[tiab]

For peer review only

OR Food Hypersensitivity[mh] OR food hypersensitivit*[tiab] OR food allerg*[tiab] OR egg allerg*[tiab] OR egg hypersensitivit*[tiab] OR milk allerg*[tiab] OR milk hypersensitivit*[tiab] OR shellfish allerg*[tiab] OR shellfish hypersensitivit*[tiab] OR wheat allerg*[tiab] OR wheat hypersensitivit*[tiab] OR nut allerg*[tiab] OR nut hypersensitivit*[tiab] OR peanut allerg*[tiab] OR peanut hypersensitivit*[tiab] OR groundnut hypersensitivit*[tiab] OR Pollen / Immunology[mh] OR Rhinitis, Allergic[mh] OR Rhinitis, Allergic, Seasonal[mh] OR Rhinitis, Allergic, Perennial[mh] OR Rhinitis[mh] OR Conjunctivitis, Allergic[mh] OR Conjunctivitis / Immunology[mh] OR Conjunctivitis / Epidemiology[mh] OR Conjunctivitis / Etiology[mh] OR allergic rhinoconjunctiviti*[tiab] OR rhinoconjunctiviti*[tiab] OR allergic rhiniti*[tiab] OR rhiniti*[tiab] OR seasonal allergic rhiniti*[tiab] OR perennial allergic rhiniti*[tiab] OR allergic conjunctiviti*[tiab] OR vernal keratoconjunctivitis*[tiab] OR vernal conjunctiviti*[tiab] OR giant papillary conjunctiviti*[tiab] OR hay fever[tiab] OR hayfever[tiab] OR pollinosis[tiab] OR pollenosis[tiab] OR nasal catarrh*[tiab])

mh = MeSH; tiab = title, abstract

Scopus

#	**Search term(s)**
1	TITLE-ABS-KEY("birth order*") OR TITLE-ABS-KEY("multiple birth*") OR TITLE-ABS-KEY("birth rank*") OR TITLE-ABS-KEY("parity")
2	TITLE-ABS-KEY("family characteristic*") OR TITLE-ABS-KEY("family size*") OR TITLE-ABS-KEY("family structure*") OR TITLE-ABS-KEY("family demograph*") OR TITLE-ABS-KEY("family composition") OR TITLE-ABS-KEY("household size*") OR TITLE-ABS-KEY("household demograph*") OR TITLE-ABS-KEY("household composition*")
3	TITLE-ABS-KEY("sibling relation*") OR TITLE-ABS-KEY("sibling*") OR TITLE-ABS-KEY("brother*") OR TITLE-ABS-KEY("sister*") OR TITLE-ABS-KEY("sibship size*") OR TITLE-ABS-KEY("sibship*")
4	TITLE-ABS-KEY("bronchial asthma*") OR TITLE-ABS-KEY("exercise-induced asthma*") OR TITLE-ABS-KEY("asthma*") OR TITLE-ABS-KEY("exercise-induced bronchospasm*") OR TITLE-ABS-KEY("respiratory hypersensitivit*") OR TITLE-ABS-KEY("airway hyper responsiveness") OR TITLE-ABS-KEY("airway hyper-responsiveness") OR TITLE-ABS-KEY("respiratory hyper responsiveness") OR TITLE-ABS-KEY("respiratory hyper-responsiveness") OR TITLE-ABS-KEY("wheez*")
5	TITLE-ABS-KEY("immediate hypersensitivit*") OR TITLE-ABS-KEY("delayed hypersensitivit*") OR TITLE-ABS-KEY("IgE-mediated hypersensitivit*") OR TITLE-ABS-KEY("type I hypersensitivit*") OR TITLE-ABS-KEY("type IV hypersensitivit*") OR TITLE-ABS-KEY("atopic sensitization") OR TITLE-ABS-KEY("atop*") OR TITLE-ABS-KEY("allergic sensitization") OR TITLE-ABS-KEY("allergic disease") OR TITLE-ABS-KEY("allerg*")
6	TITLE-ABS-KEY("atopic dermatitis") OR TITLE-ABS-KEY("dermatitis") OR TITLE-ABS-KEY("atopic eczema") OR TITLE-ABS-KEY("eczema") OR TITLE-ABS-KEY("neurodermatitis*") OR TITLE-ABS-KEY("besnier's prurigo") OR TITLE-ABS-KEY("besniers prurigo") OR TITLE-ABS-KEY("besnier prurigo") OR TITLE-ABS-KEY("quincke's edema") OR TITLE-ABS-KEY("quinckes edema") OR TITLE-ABS-KEY("quincke edema") OR TITLE-ABS-KEY("angioneurotic edema") OR TITLE-ABS-KEY("hives") OR TITLE-ABS-KEY("anaphyla*") OR TITLE-ABS-KEY("urticari*")

For peer review only

7	TITLE-ABS-KEY(”food hypersensitivit*”) OR TITLE-ABS-KEY(”food allerg*”) OR TITLE-ABS-KEY(”egg allerg*”) OR TITLE-ABS-KEY(”egg hypersensitivit*”) OR TITLE-ABS-KEY(”milk allerg*”) OR TITLE-ABS-KEY(”milk hypersensitivit*”) OR TITLE-ABS-KEY(”shellfish allerg*”) OR TITLE-ABS-KEY(”shellfish hypersensitivit*”) OR TITLE-ABS-KEY(”wheat allerg*”) OR TITLE-ABS-KEY(”wheat hypersensitivit*”) OR TITLE-ABS-KEY(”nut allerg*”) OR TITLE-ABS-KEY(”nut hypersensitivit*”) OR TITLE-ABS-KEY(”peanut allerg*”) OR TITLE-ABS-KEY(”peanut hypersensitivit*”) OR TITLE-ABS-KEY(”groundnut allerg*”) OR TITLE-ABS-KEY(”groundnut hypersensitivit*”)
8	TITLE-ABS-KEY(”allergic rhinoconjunctiviti*”) OR TITLE-ABS-KEY(”rhinoconjunctiviti*”) OR TITLE-ABS-KEY(”seasonal allergic rhiniti*”) OR TITLE-ABS-KEY(”perennial allergic rhiniti*”) OR TITLE-ABS-KEY(”allergic rhiniti*”) OR TITLE-ABS-KEY(”rhiniti*”) OR TITLE-ABS-KEY(”allergic conjunctiviti*”) OR TITLE-ABS-KEY(”vernal keratoconjunctiviti*”) OR TITLE-ABS-KEY(”vernal conjunctiviti*”) OR TITLE-ABS-KEY(”giant papillary conjunctiviti*”) OR TITLE-ABS-KEY(”hay fever”) OR TITLE-ABS-KEY(”hayfever”) OR TITLE-ABS-KEY(”pollinosis”) OR TITLE-ABS-KEY(”pollenosis”) OR TITLE-ABS-KEY(”nasal catarrh*”)
9	1 OR 2 OR 3
10	4 OR 5 OR 6 OR 7 OR 8
11	9 AND 10

Full query

(TITLE-ABS-KEY(”birth order*”) OR TITLE-ABS-KEY(”multiple birth*”) OR TITLE-ABS-KEY(”birth rank*”) OR TITLE-ABS-KEY(”parity”) OR TITLE-ABS-KEY(”family characteristic*”) OR TITLE-ABS-KEY(”family size*”) OR TITLE-ABS-KEY(”family structure*”) OR TITLE-ABS-KEY(”family demograph*”) OR TITLE-ABS-KEY(”family composition”) OR TITLE-ABS-KEY(”household size*”) OR TITLE-ABS-KEY(”household demograph*”) OR TITLE-ABS-KEY(”household composition*”) OR TITLE-ABS-KEY(”sibling relation*”) OR TITLE-ABS-KEY(”sibling*”) OR TITLE-ABS-KEY(”brother*”) OR TITLE-ABS-KEY(”sister*”) OR TITLE-ABS-KEY(”sibship size*”) OR TITLE-ABS-KEY(”sibship*”)) AND (TITLE-ABS-KEY(”bronchial asthma*”) OR TITLE-ABS-KEY(”exercise-induced asthma*”) OR TITLE-ABS-KEY(”asthma*”) OR TITLE-ABS-KEY(”exercise-induced bronchospasm*”) OR TITLE-ABS-KEY(”respiratory hypersensitivit*”) OR TITLE-ABS-KEY(”airway hyper responsiveness”) OR TITLE-ABS-KEY(”airway hyper-responsiveness”) OR TITLE-ABS-KEY(”respiratory hyper responsiveness”) OR TITLE-ABS-KEY(”respiratory hyper-responsiveness”) OR TITLE-ABS-KEY(”wheez*”) OR TITLE-ABS-KEY(”immediate hypersensitivit*”) OR TITLE-ABS-KEY(”delayed hypersensitivit*”) OR TITLE-ABS-KEY(”IgE-mediated hypersensitivit*”) OR TITLE-ABS-KEY(”type I hypersensitivit*”) OR TITLE-ABS-KEY(”type IV hypersensitivit*”) OR TITLE-ABS-KEY(”atopic sensitization”) OR TITLE-ABS-KEY(”atop*”) OR TITLE-ABS-KEY(”allergic sensitization”) OR TITLE-ABS-KEY(”allergic disease”) OR TITLE-ABS-KEY(”allerg*”) OR TITLE-ABS-KEY(”atopic dermatitis”) OR TITLE-ABS-KEY(”dermatitis”) OR TITLE-ABS-KEY(”atopic eczema”) OR TITLE-ABS-KEY(”eczema”) OR TITLE-ABS-KEY(”neurodermatitis*”) OR TITLE-ABS-KEY(”besnier’s prurigo”) OR TITLE-ABS-KEY(”besniers prurigo”) OR TITLE-ABS-KEY(”besnier prurigo”) OR TITLE-ABS-KEY(”quincke’s edema”) OR TITLE-ABS-KEY(”quinckes edema”) OR TITLE-ABS-KEY(”quincke edema”) OR TITLE-ABS-KEY(”angioneurotic edema”) OR TITLE-ABS-KEY(”hives”) OR TITLE-ABS-KEY(”anaphyla*”) OR TITLE-ABS-KEY(”urticari*”) OR TITLE-ABS-KEY(”food hypersensitivit*”) OR TITLE-ABS-KEY(”food allerg*”) OR TITLE-ABS-KEY(”egg allerg*”) OR TITLE-ABS-KEY(”egg hypersensitivit*”) OR TITLE-ABS-KEY(”milk allerg*”) OR TITLE-ABS-KEY(”milk hypersensitivit*”) OR TITLE-ABS-KEY(”shellfish allerg*”) OR TITLE-ABS-KEY(”shellfish

For peer review only

hypersensitivit*") OR TITLE-ABS-KEY("wheat allerg*") OR TITLE-ABS-KEY("wheat hypersensitivit*") OR TITLE-ABS-KEY("nut allerg*") OR TITLE-ABS-KEY("nut hypersensitivit*") OR TITLE-ABS-KEY("peanut allerg*") OR TITLE-ABS-KEY("peanut hypersensitivit*") OR TITLE-ABS-KEY("groundnut allerg*") OR TITLE-ABS-KEY("groundnut hypersensitivit*") OR TITLE-ABS-KEY("allergic rhinoconjunctiviti*") OR TITLE-ABS-KEY("rhinoconjunctiviti*") OR TITLE-ABS-KEY("seasonal allergic rhiniti*") OR TITLE-ABS-KEY("perennial allergic rhiniti*") OR TITLE-ABS-KEY("allergic rhiniti*") OR TITLE-ABS-KEY("rhiniti*") OR TITLE-ABS-KEY("allergic conjunctiviti*") OR TITLE-ABS-KEY("vernal keratoconjunctiviti*") OR TITLE-ABS-KEY("vernal conjunctiviti*") OR TITLE-ABS-KEY("giant papillary conjunctiviti*") OR TITLE-ABS-KEY("hay fever") OR TITLE-ABS-KEY("hayfever") OR TITLE-ABS-KEY("pollinosis") OR TITLE-ABS-KEY("pollenosis") OR TITLE-ABS-KEY("nasal catarrh*"))

TITLE-ABS-KEY = title, abstract, keywords

Web of Science

#	**Search term(s)**
1	TS="birth order*" OR TS="multiple birth*" OR TS="birth rank*" OR TS="parity"
2	TS="family characteristic*" OR TS="family size*" OR TS="family structure*" OR TS="family demograph*" OR TS="family composition" OR TS="household size*" OR TS="household demograph*" OR TS="household composition"
3	TS="sibling*" OR TS="sister*" OR TS="brother*" OR TS="sibship size*" OR TS="sibship*"
4	TS="bronchial asthma*" OR TS="exercise-induced asthma*" OR TS="exercise-induced bronchospasm*" OR TS="asthma*" OR TS="respiratory hypersensitivit*" OR TS="respiratory hyper-responsiveness*" OR TS="airway hyper responsiveness*" OR TS="airway hyper-responsiveness*" OR TS=wheez*
5	TS="immediate hypersensitivit*" OR TS="delayed hypersensitivit*" OR TS="IgE-mediated hypersensitivit*" OR TS="type I hypersensitivit*" OR TS="type IV hypersensitivit*" OR TS="hypersensitivit*" OR TS="atopic sensitization" OR TS="atop*" OR TS="allergic sensitization" OR TS="allergic disease*" OR TS="allerg*"
6	TS="atopic dermatitis" OR TS="dermatitis" OR TS="atopic eczema" OR TS="eczema" OR TS="neurodermatiti*" OR TS="besnier's prurigo" OR TS="besniers prurigo" OR TS="besnier prurigo" OR TS="urticari*" OR TS="anaphyla*" OR TS="quincke's edema" OR TS="quinckes edema" OR TS="quincke edema" OR TS="angioneurotic edema" OR TS="angioedema" OR TS="hives"
7	TS="food hypersensitivit*" OR TS="food allerg*" OR TS="egg allerg*" OR TS="egg hypersensitivit*" OR TS="milk allerg*" OR TS="milk hypersensitivit*" OR TS="shellfish allerg*" OR TS="shellfish hypersensitivit*" OR TS="wheat allerg*" OR TS="wheat hypersensitivit*" OR TS="nut allerg*" OR TS="nut hypersensitivit*" OR TS="peanut allerg*" OR TS="peanut hypersensitivit*" OR TS="groundnut allerg*" OR TS="groundnut hypersensitivit*"
8	TS="pollen allerg*" OR TS="allergic rhinoconjunctiviti*" OR TS="rhinoconjunctiviti*" OR TS="seasonal allergic rhiniti*" OR TS="perennial allergic rhiniti*" OR TS="allergic rhiniti*" OR TS="rhiniti*" OR TS="allergic conjunctiviti*" OR TS="vernal keratoconjunctiviti*" OR

For peer review only

	TS=”vernal conjunctiviti*” OR TS=”giant papillary conjunctiviti*” OR TS=”hay fever” OR TS=”hayfever” OR TS=”pollinosis” OR TS=”pollenosis” OR TS=”nasal catarrh*”
9	1 OR 2 OR 3
10	4 OR 5 OR 6 OR 7 OR 8
11	9 AND 10

Full query

(TS="birth order*" OR TS="multiple birth*" OR TS="birth rank*" OR TS="parity" OR TS="family characteristic*" OR TS="family size*" OR TS="family structure*" OR TS="family demograph*" OR TS="family composition" OR TS="household size*" OR TS="household demograph*" OR TS="household composition" OR TS="sibling*" OR TS="sister*" OR TS="brother*" OR TS="sibship size*" OR TS="sibship*") AND (TS="bronchial asthma*" OR TS="exercise-induced asthma*" OR TS="exercise-induced bronchospasm*" OR TS="asthma*" OR TS="respiratory hypersensitivit*" OR TS="respiratory hyper-responsiveness*" OR TS="airway hyper responsiveness*" OR TS="airway hyper-responsiveness*" OR TS=wheez* OR TS="immediate hypersensitivit*" OR TS="delayed hypersensitivit*" OR TS="IgE-mediated hypersensitivit*" OR TS="type I hypersensitivit*" OR TS="type IV hypersensitivit*" OR TS="hypersensitivit*" OR TS="atopic sensitization" OR TS="atop*" OR TS="allergic sensitization" OR TS="allergic disease*" OR TS="allerg*" OR TS="atopic dermatitis" OR TS="dermatitis" OR TS="atopic eczema" OR TS="eczema" OR TS="neurodermatiti*" OR TS="besnier’s prurigo" OR TS="besniers prurigo" OR TS="besnier prurigo" OR TS="urticari*" OR TS="anaphyla*" OR TS="quincke’s edema" OR TS="quinckes edema" OR TS="quincke edema" OR TS="angioneurotic edema" OR TS="angioedema" OR TS="hives" OR TS="food hypersensitivit*" OR TS="food allerg*" OR TS="egg allerg*" OR TS="egg hypersensitivit*" OR TS="milk allerg*" OR TS="milk hypersensitivit*" OR TS="shellfish allerg*" OR TS="shellfish hypersensitivit*" OR TS="wheat allerg*" OR TS="wheat hypersensitivit*" OR TS="nut allerg*" OR TS="nut hypersensitivit*" OR TS="peanut allerg*" OR TS="peanut hypersensitivit*" OR TS="groundnut allerg*" OR TS="groundnut hypersensitivit*" OR TS="pollen allerg*" OR TS="allergic rhinoconjunctiviti*" OR TS="rhinoconjunctiviti*" OR TS="seasonal allergic rhiniti*" OR TS="perennial allergic rhiniti*" OR TS="allergic rhiniti*" OR TS="rhiniti*" OR TS="allergic conjunctiviti*" OR TS="vernal keratoconjunctiviti*" OR TS="vernal conjunctiviti*" OR TS="giant papillary conjunctiviti*" OR TS="hay fever" OR TS="hayfever" OR TS="pollinosis" OR TS="pollenosis" OR TS="nasal catarrh*")

TS = title, abstract, author keywords, Keywords Plus

For peer review only

Appendix to

Sibship Size, Birth Order and Risk of Asthma and Allergy: Protocol for a Systematic Review and Meta-Analysis

Daniil Lisik, Athina Ioannidou, Gregorio Paolo Milani, Sungkutu Nyassi, Saliha Selin Özuygur Ermis, Giulia Spolidoro, Emma Goksör, Göran Wennergren, Bright I Nwaru

Appendix 3: Data Extraction Form

Reviewer (initials)	
Date of data extraction (yyyy-mm-dd)	
**General information**	
Author (for first author: surname, given name(s))	
Title of article	
Year of publication	
Country of origin of study	
Contact information to author(s)	
**Study characteristics**	
Study design	
Study aims/objectives	
Exposure(s) (for each exposure: 1) method(s) of assessment; 2) objectivity of assessment (objective/subjective[1]); 3) validity (yes[2]/no); 4) reliability (yes[3]/no))	
Outcome(s) (for each outcome: 1) method(s) of assessment; 2) objectivity (objective/subjective[4]); 3) validity (yes[2]/no); 4) reliability (yes[3]/no))	
Follow-up (method; length)	Method:<br>Length:<br><br>Or: ☐ not applicable
Study was conducted during (year(s))	
**Participant selection**	
Inclusion criteria	
Exclusion criteria	
Source(s) of subjects	
**Population characteristics**	
Participants recruited (n; details)	n:<br>Details:

For peer review only

Participants eligible (n; % of "Participants recruited"; eligibility criteria)	n:<br>% of "Participants recruited":<br>Eligibility criteria:
Participants included (n; % of "Participants eligible")	n:<br>% of "Participants eligible":<br><br>Or:
Participants completing follow-up (n; % of "Participants included")	n:<br>% of "Participants included":<br><br>Or:
Participants lost (n; % of "Participants included"; details; how it was dealt with)	n:<br>% of "Participants included":<br>Details:<br>How it was dealt with:<br><br>Or:
Data lost (n; % of "Participants included"; details; how it was dealt with)	n:<br>% of "Participants included":<br>Details:<br>How it was dealt with:
Participants characteristics (for each group: 1) n_{total}; 2) age (mean (SD)); 3) gender distribution (n_{males} (% of n_{total})); 4) ethnicity; 5) country; 6) economic classification of country by the World Bank; 7) setting; 8) co-morbidity)	
**Results**	
Outcomes (for each outcome for each exposure, stratified by group[5] if applicable: 1) n (% of n_{group}); 2) effect size (measure of effect) 95% CI; 3) p-value)	
**Analysis**	
Statistical analysis method	
Confounders (what confounders were identified; how they were controlled for; % of confounders controlled for)	Confounders identified:<br>How they were controlled for:<br><br>% of confounders controlled for:
Free-text interpretation of findings/conclusion	
Generalizability (is it likely that individuals selected for this study to be representative of the target population?)	
**Miscellaneous**	
Other comments/notes	

For peer review only

**Quality assessment (based on the Effective Public Health Practice Project (EPHPP) quality assessment tool[6])**	
**(A) Selection bias**	
(Q1) Is it likely that individuals selected for this study to be representative of the target population?	
(Q2) How many of eligible individuals agreed to participate in the study? (%)	
Section rating	
**(B) Study design**	
Indicate the design of the study	
Was the study setting randomized? If "No", go to (C)	
If "Yes", was the randomization method described?	
If "Yes", was the method referred to above appropriate?	
Section rating<br>Rate longitudinal studies as "moderate", and cross-sectional studies as "weak"	
**(C) Confounders**	
(Q1) Did the groups have significant differences in relation to each other prior to the intervention?	
(Q2) If "Yes", indicate how many relevant confounders that were controlled for in any way (e.g. in study design through matching, stratification, or in analysis) (%)	
Section rating<br>Rate studies without a control group as weak	
**(D) Blinding**	
(Q1) Did the outcome assessor(s) know about the exposure status of the participants?	
(Q2) Did the participants of the study know about the research question?	
Section rating<br>Rate as "weak" if Q1 is 1 and Q2 is 3	
**(E) Data collection methods**	
(Q1) Were the tools used for data collection shown to be valid?	
(Q2) Were the tools used for data collection shown to be reliable?	

For peer review only

Section rating	
**(F) Withdrawals and drop-outs**	
(Q1) Did numbers and/or reasons for withdrawals and drop-outs per group get documented?	
(Q2) How many participants completed the study (if the value is different between groups, state the lowest)? (%)	
Section rating	
**Global rating**	
Did the two reviewers give different section ratings for A-F?	
If "Yes", what is the reason for the difference(s)?	
**Final rating of both reviewers**	

[1] Objective: medical records/official statistics. Subjective: self-report, observation.
[2] Yes: The assessment gives usable, meaningful information for the research question.
[3] Yes: Results from assessment type are consistent and stable.
[4] Objective: ICD code, verified diagnosis based on medical examination. Subjective: otherwise observed or self-reported symptoms/disease.
[5] Applicable if specific stratification has been made in the analysis, e.g. significant differences and/or calculations based on gender of participants.
[6] Modified version of EPHPP [1]. Original tool is available at: https://www.ephpp.ca/quality-assessment-tool-for-quantitative-studies/. The questions and answer alternatives are modified in phrasing for readability and the nature of relevant studies. Modifications of rating are clarified in green text above, based on the modifications of EPHPP done in a systematic review by Smith et al. in 2017 [2].

References

1. Armijo-Olivo S, Stiles CR, Hagen NA, Biondo PD, Cummings GG. Assessment of study quality for systematic reviews: a comparison of the Cochrane Collaboration Risk of Bias Tool and the Effective Public Health Practice Project Quality Assessment Tool: methodological research. J Eval Clin Pract. 2012;18(1):12-18.
2. Smith M, Hosking J, Woodward A, Witten K, MacMillan A, Field A, et al. Systematic literature review of built environment effects on physical activity and active transport – an update and new findings on health equity. International Journal of Behavioral Nutrition and Physical Activity. 2017;14(1):158.

For peer review only - http://bmjopen.bmj.com/site/about/guidelines.xhtml
